# An Amino-Acid-Derived Metal–Organic Framework with Large Pores for Unspecific Enantioseparation

**DOI:** 10.1021/jacs.5c22595

**Published:** 2026-03-12

**Authors:** Xiaoyu Ma, Mengya Wang, Wenxuan Li, Jie Qi, Siyu Tu, Lei Zhang, Kun-Yu Wang, Yanming Fu, Zongsu Han, Xiang Wu, Hong-Cai Zhou, Chengfeng Zhu

**Affiliations:** † Anhui Province Key Laboratory of Advanced Catalytic Materials and Reaction Engineering, School of Chemistry and Chemical Engineering, 558979 Hefei University of Technology, Hefei 230009, P. R. China; ⊥ Department of Chemistry, 14736 Texas A&M University, College Station, Texas 77843-3255, United States; § Department of Materials Science and Engineering, 14736 Texas A&M University, College Station, Texas 77843-3003, United States; ‡ Department of Chemistry, 6740 Princeton University, Princeton, New Jersey 08544, United States

## Abstract

The selective separation of enantiomers is critical in pharmaceutical production, while conventional chiral sorbents always suffer from the trade-off between selectivity and the substrate scope. Herein, inspired by a natural unspecific peroxygenase with large protein channels, we developed a homochiral metal–organic framework (MOF) constructed from flexible phenylalanine-derived ligands and zinc ions. This MOF features a giant chiral cavity with a size of 2.2 × 3.1 nm, decorated with 42 chiral phenylalanine residues, which serves as a solid sorbent for the highly enantioselective adsorption and separation of diverse chiral compounds, including aromatic epoxides, β-nitroalcohols, mandelate derivatives, secondary alcohols, indolin-3-ones, α-methylbenzylamine, and limonene. Most importantly, benefiting from its large pores, the MOF demonstrates versatile utility in resolving the enantiomers of extraordinarily bulky substrates and structurally complex chiral pharmaceuticals, which can be further processed into a polymer matrix for membrane separation, enabling an integrated, chromatography-free route from batch-scale adsorption and separation. This material can be readily recovered and reused without an apparent loss of performance. Adsorption experiments and theoretical calculations reveal that the chiral recognition and separation originate from the distinct binding affinity of enantiomers within the MOF’s chiral pore environment, presenting a scalable platform for process-intensified chiral separations.

## Graphical abstract

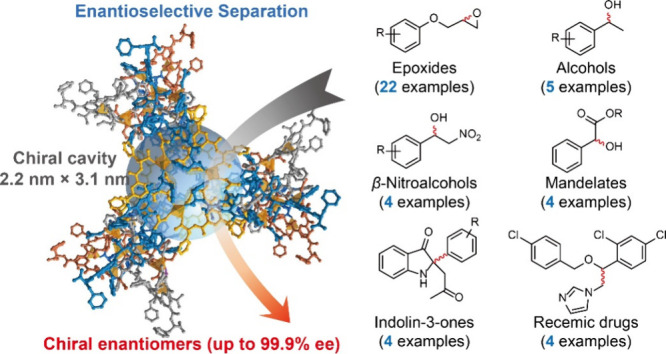

## Introduction

There is a growing demand for enantioselective separation in the pharmaceutical and agrochemical industries, as the enantiomers of a chiral molecule often exhibit remarkable differences in bioactivity.^1^ While asymmetric synthesis and crystallization are often pursued, preparative chromatography remains central for many campaigns, with practical considerations around solvent consumption, stationary-phase cost, and throughput guiding choices. These realities motivate complementary, chromatography-free, solid-phase routes that operate in stirred tanks with straightforward solid–liquid handling.^2^ Yet, chiral sorbents always suffer from the trade-off between selectivity and substrate scope because the construction of chiral sites for selective recognition is prone to blocking the transport channel of guest molecules. It is also challenging to synthesize chiral sorbents with large open pores. Enzymes are versatile biological macromolecules composed of amino acids, assembled through noncovalent interactions.^3^ The densely functional groups and chiral sites decorated within enzymes’ pocket and channel achieve their superb enantioselectivity in molecular recognition and separation.^4^ However, most enzymes feature high specificity in applications, which means that the substrate scope is usually limited. One exceptional case is the unspecific peroxygenase (UPO) secreted by fungi, such as MroUPO secreted from *M. rotula*, which enables catalyzing the oxidation of a broad range of substrates, benefiting from its large protein channel around 2 nm stabilized by multiple noncovalent interactions.^5^ Herein, inspired by the design principle of the natural enzyme UPO, we intend to utilize multiple molecular interactions to construct large pores to promote mass transfer in MOFs, without sacrificing the enantioselectivity in separation by introducing dense chiral centers. Such enzyme-inspired materials will provide prospects for the efficient production of diverse optically pure enantiomers.

Metal–organic frameworks (MOFs), assembled from metal nodes and organic linkers, provide an ideal platform for designing materials for diverse separations, catalysis, and other fields, owing to their unique advantages including modular synthesis, well-defined crystallographic structures, high porosity, and chemical tunability.^6^ In particular, the judicious incorporation of chirality and functionality into the pore of MOFs can create a unique microenvironment for recognizing and separating enantiomers.^7^ In the last decades, a significant number of homochiral microporous MOFs, based on enantiopure amino acids, BINOL, Schiff base, biphenyl, and so on, have been constructed and applied in the resolution of enantiomers via cocrystallization, chromatography, and membrane separation, and the separated substrate scope covers racemic alcohols, amines, sulfoxides, and amino acids.^8^ It is recognized that highly enantioselective recognition inherently relies on synergistic noncovalent interactions between racemic substrates and functional sites within chiral MOFs, including π–π stacking, hydrogen bonding, and electrostatic interactions.^9^ Besides, large pore structures in chiral MOFs further facilitate matching the molecular sizes of diverse substrates, even enabling the accommodation of complex chiral pharmaceuticals of which the ingress is often inaccessible for chiral adsorbents with limited pore sizes.^10^ Notably, many chiral MOFs, especially those with large pores exceeding 2 nm, have demonstrated promising applications in enantioselective catalysis.^11^ Yet, their performance in enantioselective separation remains largely underexplored, and chiral MOFs that simultaneously exhibit high enantioselectivity and broad substrate versatility are still scarce.^12^ Consequently, the design and development of new chiral MOFs integrating dense recognition sites and well-defined large pores for practical enantioseparation remains an urgent priority.

Using amino acids to construct enzyme-mimetic chiral MOFs is an effective and low-cost strategy for introducing abundant chiral sites to recognize and separate enantiomers.^13^ For instance, the chiral MOF Cu­(GHG), built from a tripeptide ligand, demonstrates excellent performance in the separation of racemic methamphetamine and ephedrine, leveraging its multiple functional sites for enhanced selectivity.^14^ Yet, it remains a challenge to construct chiral MOFs with pores over 2 nm to achieve a broad substrate scope due to the uncertain coordination mode, high flexibility, and low symmetry of amino-acid-derived ligands, which can easily lead to close packing to afford nonporous structures.^15^ To address this issue, we present herein a mesoporous chiral MOF featuring a large inner cavity of 2.2 × 3.1 nm in size, achieved by selecting a phenylalanine-derived carboxylic acid ligand with a *C*_3_ symmetric structure and well-balanced rigidity-flexibility. The peptide moieties and phenyl rings in the ligand introduce diverse supramolecular interactions that stabilize the large pores. Impressively, one single cavity is decorated with 42 amino acid residues on the interior wall. The MOF can be utilized as a versatile chiral solid sorbent to separate diverse racemic molecules, including aromatic epoxides, β-nitroalcohols, mandelate derivatives, secondary alcohols, indolin-3-ones, α-methylbenzylamine, limonene, and commercial drugs, achieving enantiomeric excess (ee) values of up to 99.9%.

## Results and Discussion

### Synthesis and Characterization

The *C*_3_ symmetric enantiopure ligand, H_3_ **L**, was readily prepared from (*S*)- or (*R*)-phenylalanine in three steps in about 80% overall yield. Heating the mixture of H_3_ **L** and Zn­(NO_3_)_2_·6H_2_O in a mixed solvent of *N*,*N-*dimethylformamide (DMF), ethanol (EtOH), and water (H_2_O) at 65 °C for 3 days produced colorless block crystals of [Zn**L**·DMF] (**1**) with a yield of ca. 65% based on H_3_ **L**. The as-synthesized product of **1** was stable in common organic solvents such as dichloromethane, chloroform, acetone, acetonitrile, methanol, and ethanol. The chemical structure of **1** was characterized by a variety of techniques, including single-crystal/powder X-ray diffraction (XRD), infrared spectroscopy (IR), UV–vis spectroscopy, thermogravimetric analysis (TGA), X-ray photoelectron spectroscopy (XPS), and elemental analysis (EA).

Single-crystal X-ray diffraction (SC-XRD) unambiguously revealed that **1** is an infinite three-dimensional (3D) metal–organic framework that crystallizes in a chiral cubic *I*2_1_3 space group with a unit cell of *a* = *b* = *c* = 38.246(6) Å and *V* = 55945(9) Å^3^ (Figure 1). The asymmetric unit cell of **1** contains one fully deprotonated **L** ligand, one divalent zinc ion, and one coordinated DMF molecule. In the structure of **1**, the chiral ligand **L** exhibits a folded conformation after its coordination with zinc ions due to the flexibility of the amide group, where three phenylalanine residues are perpendicular to the ligand’s central benzene ring (Figure 1a). The central zinc ion adopts a distorted tetrahedral geometry by coordinating to three monodentate carboxylate groups from three different **L** ligands and one oxygen atom from the coordinated DMF molecule, with the Zn–O bond lengths varying from 1.91(1) to 2.03(7) Å. The three adjacent zinc ions (mutual Zn–Zn distance of 8.82 Å) featuring *C*_3_ symmetry are held together through three bridging ligands and intermolecular C–H···π interactions (2.77 and 2.83 Å) among three adjacent phenylalanine residues, forming a Zn_3_ **L**_3_ building unit (Figure 1a). The resulting Zn_3_ **L**_3_ motifs can act as a planar three-connected linker, each would bind three surrounding ones at a dihedral angle of 70.5° with each other through the Zn–O coordination bonds and noncovalent interactions, further leading to a 3D chiral coordination network with **tht** topology (Figure 1b). Moreover, two pairs of intermolecular N–H···O (2.05 Å and 2.22 Å) hydrogen bonding between the neighboring amide groups from the adjacent **L** ligands stabilize the flexible framework structure as well.^3b^ Along the [111] direction, the framework of **1** possesses a chiral triangular channel with the largest side of about 1.98 nm (Figure 1b and Figure S1). Taking a closer look at the crystal structure, we note that 14 Zn_3_ **L**_3_ units are connected to form an oblate-lantern-typed cage of approximately 2.2 nm × 3.1 nm in width and height (Figure 1c), with 42 phenylalanine residues located on the faces. Meanwhile, these resulting giant cages are passed through with a *C*_3_ axis and interconnected with each other through their three irregular triangular apertures, each with a side length of approximately 3.3 nm on their face, generating open chiral channels for mass transmission (Figure S2). The corresponding solvent-accessible free volume for such porous structure **1** was estimated to be about 65.1% using PLATON software. Additionally, the inner surfaces of the cages and channels are periodically decorated with dense phenylalanine residue, similar to the chiral microstructure of enzymes, providing potential functional sites for recognizing and distinguishing the enantiomers of chiral molecules. To our knowledge, chiral metal–organic frameworks assembled from flexible amino-acid ligands and featuring large internal pores are still relatively rare.^16^

**Figure 1:**
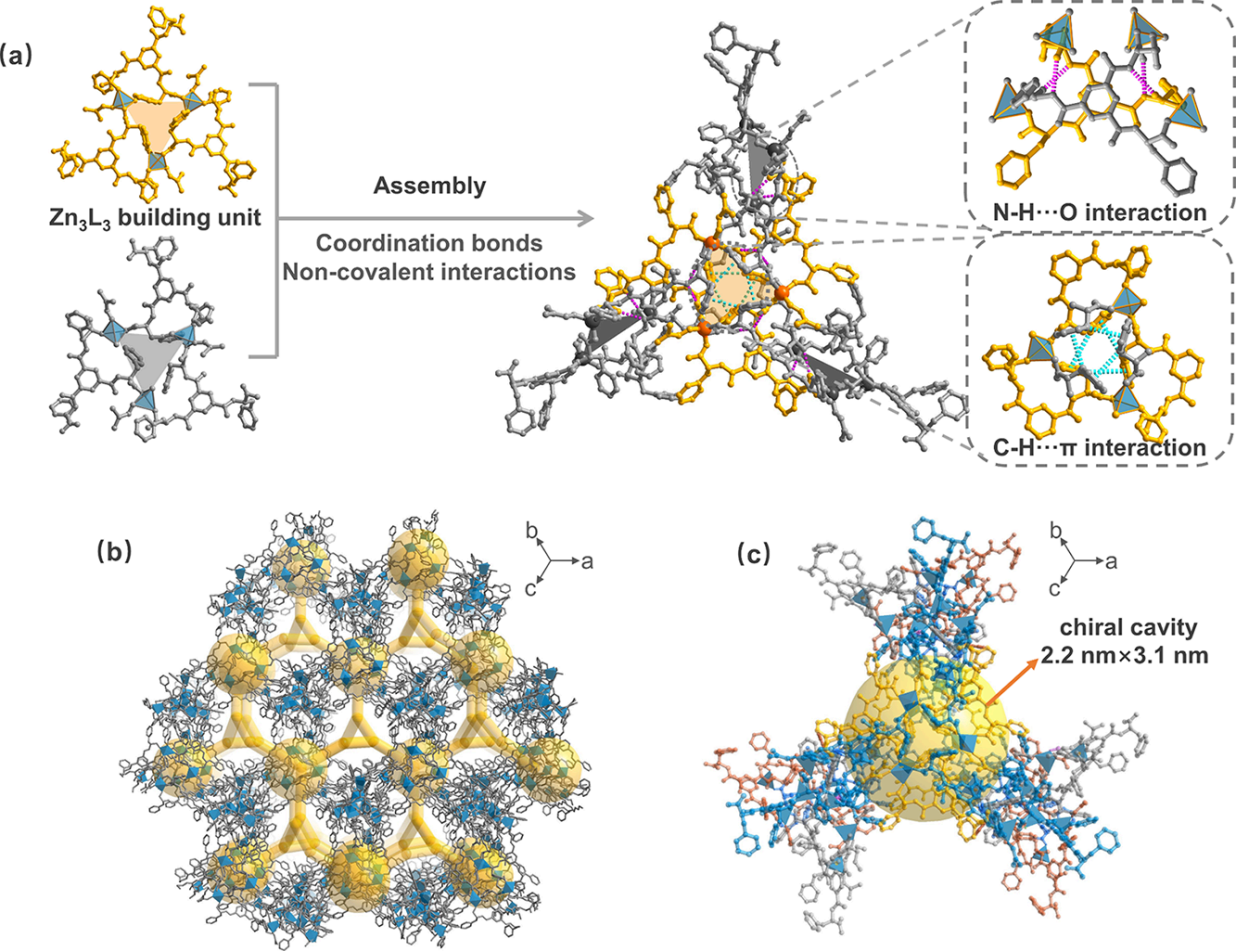
(a) Illustration of the folded conformation of the flexible ligand within the Zn_3_ **L**_3_ building unit and the noncovalent interactions (N–H···O and C–H···π) involved in the assembly process. (b) The three-dimensional architecture with interconnected pores, which are represented in yellow rods. (c) The large chiral inner cavity surrounded by amino acid residues, wherein the yellow sphere represents the cavity of 2.2 nm × 3.1 nm.

The powder X-ray diffraction (PXRD) pattern of pristine crystals of **1** is consistent with the one simulated from its single-crystal structure, demonstrating the phase purity of its bulk samples (Figure S3). The IR spectrum shows the loss of a characteristic peak for the carboxyl stretch ν_C=O_ at 1725 cm^–1^ originating from the free H_3_ **L** ligand after the complexation with zinc ions, indicating the formation of a coordination bond of Zn–O (Figure S4). The UV–vis spectrum reveals that the coordination network of **1** exhibits similar absorption bands with the bridging H_3_ **L** ligand at approximately 260 and 350 nm, which can be assigned to the π–π* and n−π* transitions of the chiral ligand, respectively (Figure S5). TGA analysis indicates that the guest DMF, EtOH, and water molecules in **1** can be lost when the temperature reaches up to 180 °C, and then, the framework would be decomposed around 300 °C under a nitrogen atmosphere (Figure S6). Nitrogen sorption measurements at 77 K reveal only surface adsorption for **1**, probably attributed to the structural collapse of the flexible framework upon removal of solvent molecules, which serve as structural supports under high-temperature and vacuum conditions.^17^ Nevertheless, to probe the accessibility of the large pores in **1** for chiral molecules under liquid-phase conditions, we conducted dye adsorption experiments, which is an alternative, well-established method for evaluating the porosity of MOFs with large pores or channels.^17^ Furthermore, chiral sorption in **1** takes place via dynamic exchange with solvents within nanopores, differing fundamentally from gas adsorption.^14^ By soaking the solvent-exchanged crystals in 1 mM ethanol solutions of methylene blue (MB), crystal violet (CV), and rhodamine B (RhB), they have different molecular sizes. The originally colorless crystals turned distinctly blue, purple, and red over time, indicating successful dye encapsulation. UV–vis monitored adsorption experiments indicated that **1** can uptake about 83%, 72%, and 69% of the total dye molecules for MB, CV, and RhB, respectively, at the adsorption equilibrium (Figure S7). However, almost no adsorption was observed for Evans blue (EB), whose size exceeded the pore size of **1**. Such size-selective uptake confirms that adsorption occurs specifically within the large pores, demonstrating the presence of accessible pores in the solvent system.

### Enantioselective Adsorption and Separation

Considering the presence of large chiral internal cavities and interconnected open channels in the framework of **1** for accommodating a range of analytes as well as the densely and uniformly distributed phenylalanine residues on the wall of the framework, which may be beneficial for bonding guest molecules, prompts us to investigate its potential applications in the enantioselective separation of racemic compounds. Herein, we selected epoxides as the chiral substrates to study the enantioselectivity of **1** because such compounds are one class of important synthons in synthetic and pharmaceutical chemistry due to their epoxy rings readily reacting with various nucleophiles and producing a range of multipurpose chiral organic functionalities. Initial enantiosorption studies identified the solvent-exchanged **1** as an excellent sorbent for epoxides, and a variety of solvents were screened for selectivity with styrene oxide (SO) as a model substrate. Upon solvent screening, the solvent-exchanged crystals of (*S*)-**1** were immersed in racemic SO, in six different solvents, at room temperature for 6 h; then, the sorbent was filtered and washed with fresh solvent several times to extract the encapsulated chiral enantiomers within it. After which, the enantiopurity of the desorbed SO molecules was analyzed by chiral high-performance liquid chromatography (HPLC). The results indicate that acetone was the most suitable solvent for the enantiosorption, affording the (*S*)-enantiomer of SO with approximately 99.0% ee following extraction with dichloromethane (Table 1, Figure S9). It is presumed that the appropriate solubility and polarity of such solvents facilitate the mass transfer of chiral substrates within this MOF.^18^ When (*R*)-**1** was employed as the sorbent, the (*R*)-enantiomer of SO with 99.3% ee can be obtained, indicating that the chirality of the host sorbent controls the inclusion of the racemic epoxide.

**Table 1:**
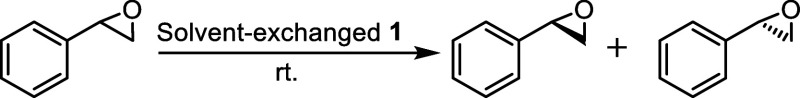
Enantiosorption of **1** to Racemic Styrene Oxide

entry	solvent	adsorbent	ee (%)
1	THF	(*S*)-**1**	52.5
2	CH_3_CN	(*S*)-**1**	72.2
3	CH_2_Cl_2_	(*S*)-**1**	82.1
4	CHCl_3_	(*S*)-**1**	75.5
5	EtOH	(*S*)-**1**	84.3
6	acetone	(*S*)-**1**	99.0
7	acetone	(*R*)-**1**	99.3

The enantiosorption performance of **1** toward the two enantiomers of epoxides was further investigated through three sets of quantitative enantiosorption experiments. At room temperature, 50 mg of (*S*)-**1** crystals was immersed in 1.5 mL of an acetone solution of styrene oxide (SO), glycidyl phenyl ether (GPE), and glycidyl triphenylmethyl ether (GTE) at the same concentration of 1.0 mM. Then, three chiral epoxides with different structures and sizes in the supernatant were analyzed by chiral HPLC in terms of peak area and ee value with time. As shown in Figure 2a, the total peak areas of two enantiomers for the SO molecule gradually decreased from ∼20467 to ∼10778 after the sorption of 6 h, suggesting that ∼47% of the total SO molecules can be encapsulated by (*S*)-**1**. In addition, the SO molecules in the supernatant yielded an ee value of ∼62% with the (*R*)-enantiomer in excess, further indicating that the (*S*)-enantiomer is adsorbed preferentially by (*S*)-**1** (HPLC spectra are provided in the Supporting Information, Figure S10). Similar adsorption behaviors occurred when the SO was replaced by GPE and GTE under identical conditions. (*S*)-**1** can selectively adsorb ∼43% of GPE enantiomers and ∼32% of GTE enantiomers, respectively, affording the corresponding GPE and GTE molecules with ee values of ∼58% and ∼38% (Figure 2b,c). Although the adsorption amounts and ee values of the substrates would get smaller with the increase in size of the epoxides, it was found that the enantioselective adsorption behaviors of (*S*)-**1** toward the two isomers of epoxides have become more apparent. Notably, nearly only one enantiomer was encapsulated by (*S*)-**1** during the whole adsorption procedure for the larger substrate GTE molecules. In addition, the enantiosorption performance of (*S*)-**1** was further studied through the adsorption of enantiopure GTE isomers at 25 and 35 °C. It was found that (*S*)-**1** possesses a remarkable adsorption amount for the (*S*)-enantiomers of GTE, which is significantly greater than that for the (*R*)-enantiomers, further demonstrating the highly enantioselective nature of (*S*)-**1** to epoxides (Figure 2d,e). We presumed that the excellent enantioselectivity of (*S*)-**1** in recognizing racemates originates from its amphipathic chiral microenvironment, which is composed of hydrophobic benzyl groups and hydrophilic peptide bonds. These dense chiral functional moieties enable the formation of asymmetric multiple supramolecular interactions (e.g., hydrogen bonding, π–π stacking, C–H···π interactions) with chiral epoxide molecules, thereby achieving highly selective discrimination of different enantiomers of epoxide.^9c,19^ The crystallographic structures of the host–guest complexes could not be resolved, primarily due to the weak diffraction signal of the single crystals of (S)-**1** containing chiral epoxide molecules. Consequently, to understand the nature of enantioselectivity of the porous chiral host framework toward adsorbate molecules, theoretical calculations were conducted using the grand canonical Monte Carlo (GCMC) method. There was a moderate binding energy difference for (*R*)- and (*S*)-enantiomers (Figure S11). For instance, the (*S*)- and (*R*)-GPE feature an ∼8 kJ/mol difference in adsorption energy, which may be originated from nonbonding interactions such as hydrogen bonding.

**Figure 2:**
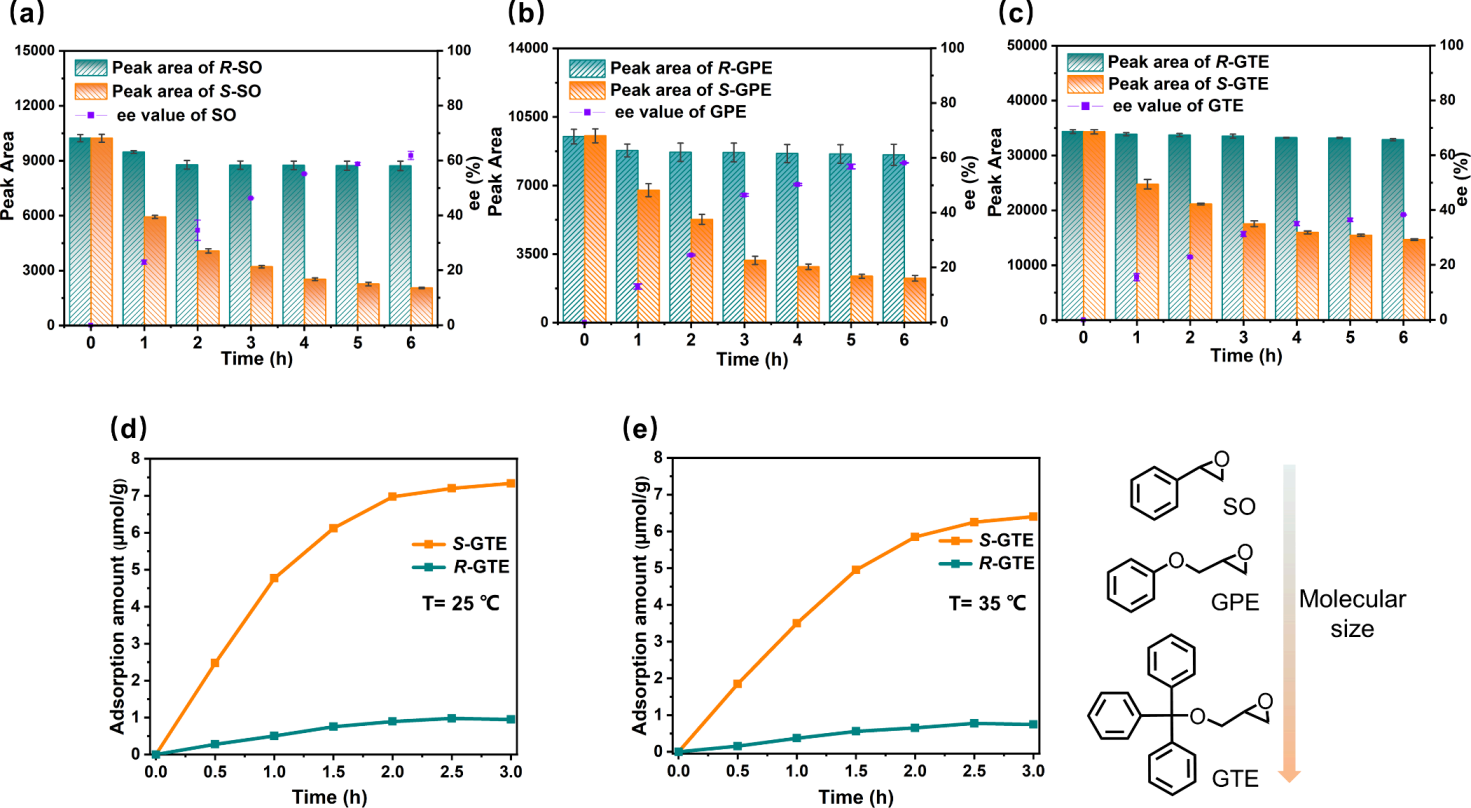
(a–c) Enantiosorption profiles of racemic epoxides SO (a), GPE (b), and GTE (c) using (*S*)-**1** as the chiral sorbent, depicted by enantiomeric peak areas and ee values as a function of contact time. (d, e) Adsorption of enantiopure (*S*)-GTE and (*R*)-GTE isomers by (*S*)-**1** at 25 °C (d) and 35 °C (e). The chemical structures of SO, GPE, and GTE are illustrated on the right.

Given the remarkable enantioselective adsorption and separation ability of this porous framework for the epoxide enantiomers, we next examined the substrate scope of (*S*)-**1** in the enantioseparation of epoxides under the established method. First, a variety of racemic SO analogues with 4-Me, 4-MeO, 4-F, 4-Cl, and 4-Br substituents on the aromatic ring can be smoothly separated by (*S*)-**1**, giving rise to very high enantioselectivities with ee values ranging from 98.9% to 99.9%, regardless of the electronic nature of the substituent (Figure 3, **1a**–**1f**). Second, glycidyl phenyl ether (GPE), a class of phenoxy epoxides, and its analogues with electron-rich or -deficient groups on the aromatic ring, even including bulkier substrates 1-naphthylmethyl glycidyl ether, 2-naphthylmethyl glycidyl ether, and benzyl glycidyl ether, could be resolved by this chiral solid sorbent as well, affording ee values ranging from 97.8 to 99.9% (Figure 3, **1g**–**1r**). When the strong electron-withdrawing nitro groups, such as 4-NO_2_ and 2-NO_2_, were introduced on the aromatic ring of GPE, the substrates **1s** and **1t** were resolved with comparable enantioselectivity (96.7 and 95% ee, Figure 3, **1s**–**1t**) to the parent epoxide. Finally, the racemic epoxides with significant steric hindrance, such as 4-pentylphenyl glycidyl ether and triphenylmethyl glycidyl ether, were also applied in the enantioseparation, yielding ee values of 92.8% and 94.7%, respectively (Figure 3, **1u**–**1v**). The broad substrate scope and excellent enantioselectivity indicate that (*S*)-**1** is one of the best examples of a chiral sorbent for separating racemic epoxides so far.^20^

**Figure 3:**
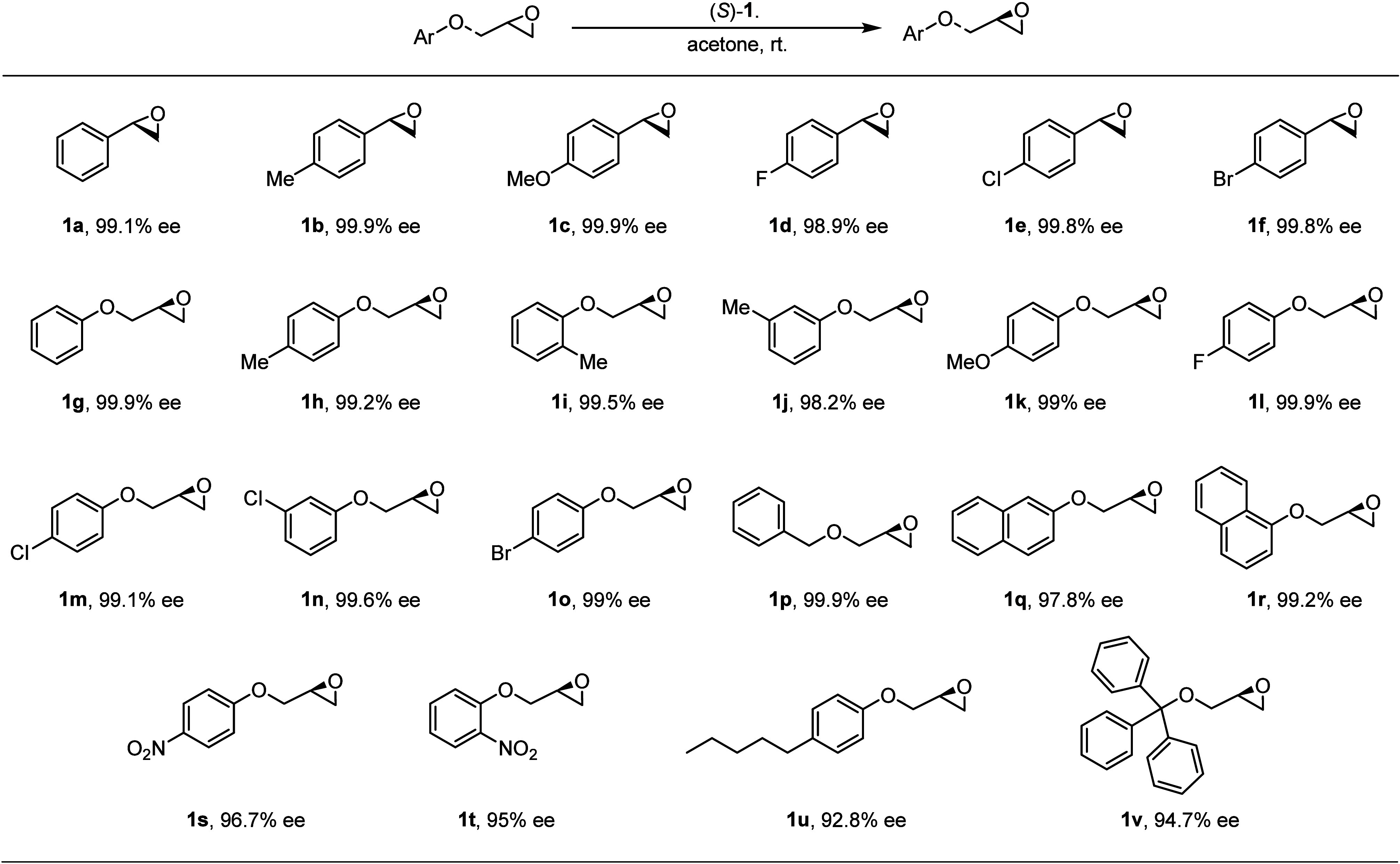
Substrate scope and ee values of epoxides were resolved by chiral solid sorbent (*S*)-**1**.

The recyclability and reusability of (*S*)-**1** were evaluated by using the same batch of crystals for the consecutive five-cycle separation of racemic SO. It was found that the chiral sorbent (*S*)-**1** could be easily recovered by filtration and reused for the subsequent runs of enantioseparation after full washing, desorption, and solvent exchange. The ee value of desorbed SO for each cycle could be kept at ca. 99% without the deterioration of enantioselectivity (Figure S13). In addition, the PXRD measurement of the recovered (*S*)-**1** crystals indicated that the crystallinity is retained after the consecutive enantioseparation reactions (Figure S3). The above results suggest excellent recyclability of (*S*)-**1** under the established separation conditions, demonstrating that (*S*)-**1** is a promising chiral solid sorbent for practical enantioseparation.

Encouraged by the excellent enantioselectivity and recycling ability of (*S*)-**1** in the resolution of epoxides, we again attempted to investigate whether it has the capacity to resolve other chiral molecules with diverse structures, functions, and sizes. First, 2-nitro-1-phenylethanol (NPE) and its derivatives were selected as analytes because their enantiopure enantiomers, featuring an easily functionalized β-OH group and a readily reducible −NO_2_ group, are versatile synthetic intermediates for chiral β-blockers, agrochemicals, and other compounds.^21^ Gratifyingly, under the optimized enantioseparation conditions, (*S*)-**1** could easily separate racemic NPE molecules and their derivatives into enantiopure isomers, with ee values ranging from 99.3% to 99.9% (Figure 4, **2a**–**2d**). Next, enantiopure methyl mandelate (MM) and 1-phenylethanol (1-PE), both featuring an α-OH functional group, are valuable chiral building blocks for diverse applications in pharmaceuticals, as well as in organic synthesis and analytical chemistry.^22^ It was found that MM, PE, and their corresponding analogues can be readily resolved using chiral sorbent (*S*)-**1**, affording optically pure products with ee values of 99.0–99.9%. (Figure 4, **3a**–**3d**, **4a**–**4d**). Particularly, the racemic secondary alcohol **4e**, with a molecular size of about ca. 2.02 × 1.4 × 0.7 nm, is successfully separated over (*S*)-**1** with 99.1% ee. To the best of our knowledge, this is the largest chiral alcohol example that has been resolved by a chiral MOF sorbent, indicating its promising separation of large chiral drug molecules. In addition to its capacity for resolving the aforementioned oxygen-containing racemic substrates, including epoxides, β-nitroalcohols, mandelate derivatives, and secondary alcohols, (*S*)-**1** also exhibits remarkable resolution capability toward racemic indolin-3-ones, which are oxygen- and nitrogen-containing compounds with a quaternary carbon atom. Such compounds are essential components of several biologically active species, including isatisine A, austamide, and aristotelone.^23^ All examined indolin-3-ones can be resolved with ee values of up to 99.9% (Figure 4, **5a**–**5d**). Notably, such chiral products with stereogenic quaternary carbon centers are generally obtained *via* asymmetric synthesis. This work not only provides an alternative approach for preparing enantiopure indolin-3-one isomers but also represents the first example of enantioseparation of indolin-3-ones using a chiral MOF-based sorbent. In particular, high ee values were achieved for α-phenylethylamine (99.3%) and limonene (99.9%) as well, further confirming the broad applicability of this enantioselective separation process across diverse molecules. Finally, the applicability of (*S*)-**1** for separating chiral drugs was evaluated. Chiral naproxen, omeprazole, tropicamide, and econazole with distinct functionalities, pharmacological activities, and molecular sizes were successfully resolved into optically pure drugs using (*S*)-**1**, demonstrating its remarkable utility (Figure 4, **6a**–**6d**). Collectively, the exceptional versatility and superb enantioselectivity of (*S*)-**1** clearly showed that it represents one of the best chiral MOF-based sorbents for the adsorption separation of racemates reported to date. It is assumed that the dense recognition sites derived from amphipathic phenylalanine residues, along with sizable chiral cavities within the (*S*)-**1** framework, enable highly efficient mass transport and bioanalogous interactions between chiral species and the framework, thereby resulting in excellent chiral separation performance.

**Figure 4:**
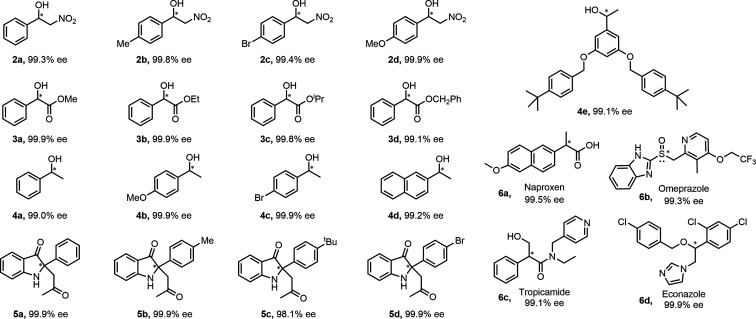
Substrate scope and ee values of racemic molecules and drugs resolved by chiral solid sorbent (*S*)-**1**.

The selective separation of enantiomers using mixed matrix membranes (MMM) is a promising procedure for obtaining enantiopure products due to its advantages in continuous operation for practical applications.^8d,24^ Herein, we prepared a (*S*)-**1**-based MMM using polyvinylidene fluoride (PVDF) as the polymeric matrix through a solution casting method.^25^ Chiral separation performance of (*S*)-**1**-based MMM with a 20 wt % MOF loading was examined by using a homemade diffusion cell, in which the 2.0 mM racemic SO was added to the feed chamber and pure acetone was added to the permeate chamber. It was found that the ee value of SO molecules collected from the permeate side at 10 min reaches up to 99.1%. When the analyte is converted into 1-PE, enantiopure 1-PE molecules with an ee value of up to 97.1% can be obtained after 30 min (Figure 5). However, the PVDF membrane alone cannot separate the enantiomers. Thus, the as-prepared MMM inherited the enantioselectivity of the chiral porous material (*S*)-**1**, further demonstrating its promising practical application in the resolution of racemic chiral molecules. Therefore, the enzyme-mimicking framework, assembled from amino acids, represents a new generation of chiral solid sorbents capable of chiral separation of a variety of racemic molecules with high enantioselectivity and efficiency.

**Figure 5:**
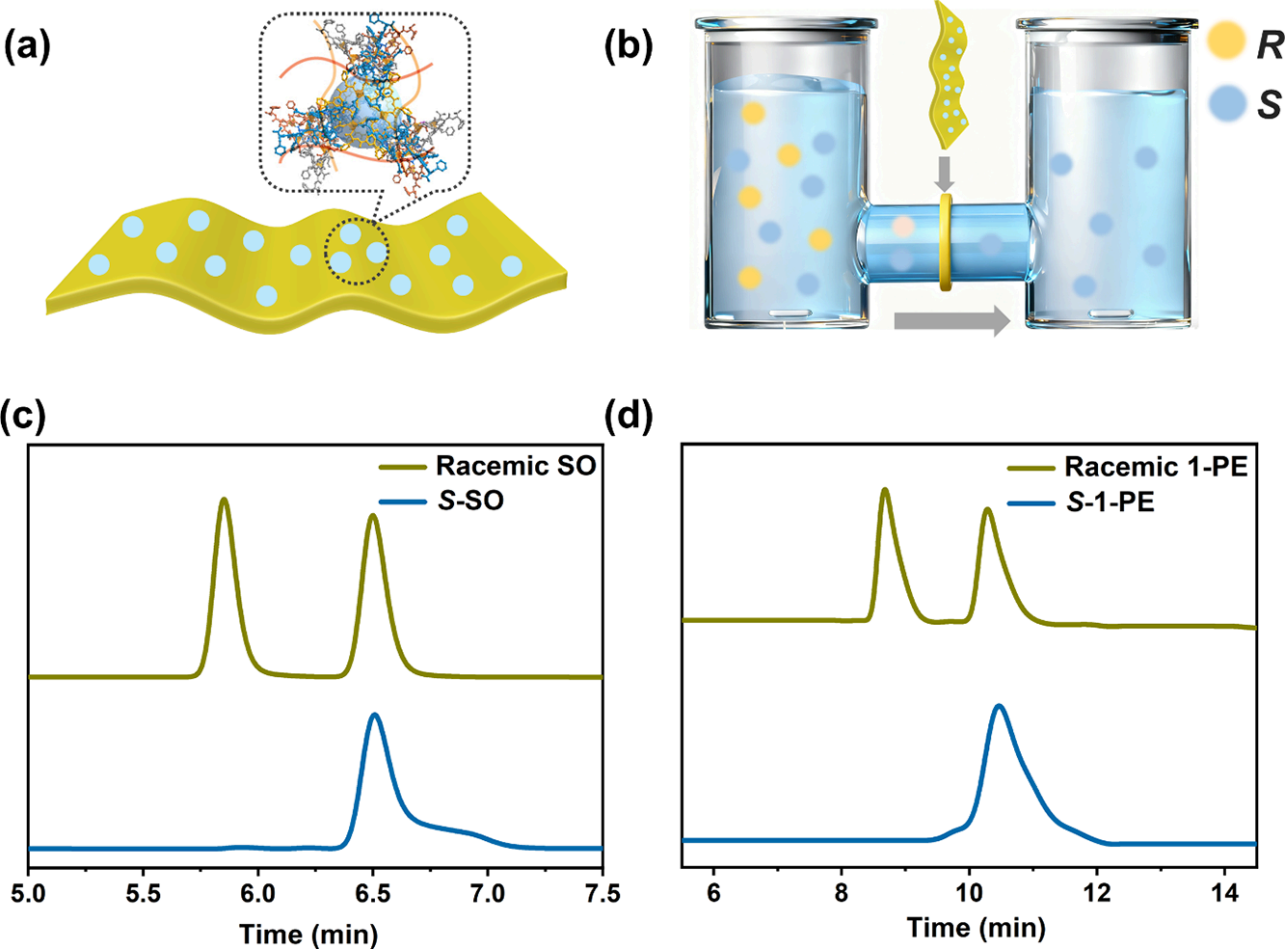
Scheme of (a) (*S*)-**1**/PVDF mixed matrix membranes ((*S*)-**1**-based MMM) and (b) homemade membrane separation setup. Liquid chromatograms of styrene oxide (SO) (c) and 1-phenylethanol (1-PE) (d) separated by (*S*)-**1**-based MMM.

## Conclusions

In conclusion, we have presented the assembly of an enzyme-inspired homochiral MOF featuring an inner cavity over 3 nm, decorated densely with chiral sites from the highly flexible amino-acid-derived ligand. The MOF demonstrated excellent enantioselectivity (up to 99.9% ee) and efficiency to separate a variety of racemic molecules with different functionalities and sizes, including aromatic epoxides, β-nitroalcohols, secondary alcohols, mandelate derivatives, indolin-3-ones, α-methylbenzylamine, limonene, and commercial drugs. Among all of the examples, the largest molecule features a size of over 2.0 nm. This work presents one prototypic approach to constructing large chiral pores, providing design principles for future development of enzyme-mimicking materials for chiral separation, sensing, and catalysis.

## Supplementary Material



## Notes

Experimental procedures and characterization data (PDF)
