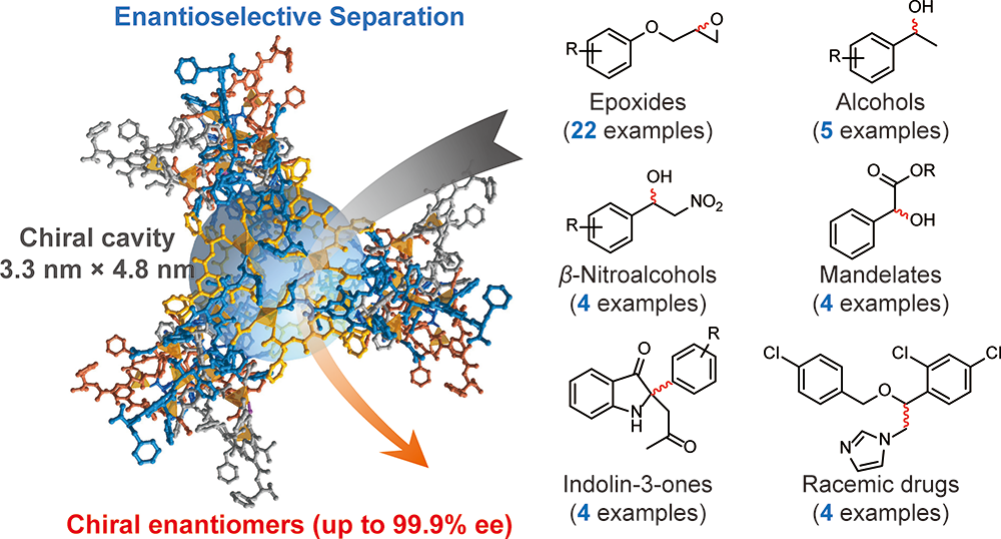# Correction to “An Amino-Acid-Derived Metal–Organic Framework with Large Pores for Unspecific Enantioseparation”

**DOI:** 10.1021/jacs.6c08555

**Published:** 2026-06-02

**Authors:** Xiaoyu Ma, Mengya Wang, Wenxuan Li, Jie Qi, Siyu Tu, Lei Zhang, Kun-Yu Wang, Yanming Fu, Zongsu Han, Xiang Wu, Hong-Cai Zhou, Chengfeng Zhu

In the Results and Discussion, in the second paragraph under “Synthesis and Characterization” (page 12026), an error appeared in the text: in “...further leading to a 3D chiral coordination network with **tht** topology (Figure 1b).” the topology symbol was incorrectly written as **tht** instead of **sin**.

In addition, in the lower right corner of the TOC graphic, the word “racemic” was mistakenly written as “recemic”. The corrected graphic is shown below: