# Supplementary material for: An Amino-Acid-Derived Metal–Organic Framework with Large Pores for Unspecific Enantioseparation
Source: J Am Chem Soc. 2026 Mar 12;148(11):12024–32. doi: 10.1021/jacs.5c22595 (PMC13022874; doi:10.1021/jacs.5c22595)
Supplement: Supplementary file 1 [file ja5c22595_si_001.pdf]

An Amino-Acid-Derived Metal-Organic Framework with Large Pores for Unspecific Enantioseparation

Xiaoyu Ma,[†#] *Mengya Wang,*[†#] *Wenxuan Li,*[‡#] *Jie Qi,*[†] *Siyu Tu,*[†] *Lei Zhang,*[†] *Kun-Yu Wang,* *[⊥] *Yanming Fu,*[†] *Zongsu Han,*[⊥] *Xiang Wu,* * *Hong-Cai Zhou**[⊥, §] *and Chengfeng Zhu**[†]

[†]Anhui Province Key Laboratory of Advanced Catalytic Materials and Reaction Engineering, School of Chemistry and Chemical Engineering, Hefei University of Technology, Hefei, 230009, P. R. China.

[⊥]Department of Chemistry, Texas A&M University, College Station, Texas 77843-3255, United States.

§Department of Materials Science and Engineering, Texas A&M University, College Station, Texas 77843-3003, United States

[‡]Department of Chemistry, Princeton University, Princeton, New Jersey, 08544, United States

Table of Content

1. Materials and General Procedures. 1
2. Synthesis of chiral ligand (*S*)-H_3L and chiral MOF (*S*)-1 3
3. Table S1. Crystal data and structure refinement for (*S*)-1. 4
4. Table S2. Selected bond lengths [Å] and angles [°] for (*S*)-1. 4
5. Figures S1-S2. X-ray crystallographic structure of (*S*)-1. 5
6. Figure S3. PXRD patterns of (*S*)-1. 5
7. Figure S4. IR spectra of H_3L and (*S*)-1. 6
8. Figure S5. UV-vis spectrum of H_3L and (*S*)-1. 6
9. Figure S 6. TGA curves of (*S*)-1. 6
10. Figure S7. Dynamic adsorption of dye molecules by (*S*)-1 monitored by UV-Vis. 7
11. Figure S 8. XPS data of (*S*)-1 7
12. Figure S9. HPLC results from the solvent screen (in Table 1). 8
13. Figure S10. HPLC analysis of dynamic enantiosorption by (S)-1. 12
14. Figure S11. Binding energy difference for (R)- and (S)- enantiomers of epoxides resolved by (*S*)-1. 45
15. Figure S12. HPLC spectra of epoxides (in Figure 3). 45
16. Figure S13. HPLC spectra for the cycling enantioseparation. 67
17. Figure S14. HPLC spectra of *β*-nitroalcohols (in Figure 4). 70
18. Figure S15. HPLC spectra of mandelate derivatives (in Figure 4). 74
19. Figure S16. HPLC spectra of secondary alcohols (in Figure 4). 78
20. Figure S17. HPLC spectra of indolin-3-ones (in Figure 4). 83
21. Figure S18. HPLC spectra of *α*-phenylethylamine and limonene. 87
22. Figure S19. HPLC spectra of chiral drugs (in Figure 4). 89
21. References. 93

1. Materials and General Procedures.

All chemicals, including the racemic substrates, are commercially available and used as received without further purification. Infrared spectra (KBr particles) were recorded on a Nicolet Magna 750 FTIR spectrometer over the range of 400-4000 cm^{-1}. Thermogravimetric analysis (TGA) is performed on the TGA-50 thermogravimetric analyzer under a nitrogen atmosphere with a heating rate of 5 °C per minute. UV-Vis absorption measurements for the dye uptake study were acquired using an Agilent Cary UV-Vis spectrophotometer. The simulated PXRD pattern was generated from the single-crystal structure of (*S*)-**1** using Mercury software. The experimental pattern was collected at room temperature on a DMAX2500 diffractometer with Cu Kα radiation. Analytical high-performance liquid chromatography (HPLC) was performed using an Agilent Technologies Model 1260 Infinity II with a UV detector and ChiralCel OD/IA/OJ-H/AS-H/AD-H/AD-3 analytical column.

X-ray Crystallography. Single-crystal X-ray diffraction (SC-XRD) data for compound (S)-**1** were collected at 100 K on BL17B beamline of Shanghai synchrotron radiation facility, using a wavelength of 0.65251 Å. Multiple datasets were acquired, from which the one with the highest resolution (approximately 1.1 Å) was selected for processing through indexing, integration, and scaling with the APEX4 program. Data reduction, including indexing, integration, and scaling, was performed using the **APEX4** software suite. The structure of (S)-**1** was solved by the direct methods using SHELXS-2018 and refined with SHELXL-2018 within *OLEX 2-1.2* software package[1]. All the hydrogen atoms attached to the chiral ligand were placed in calculated positions and refined using a riding model. Due to the weak diffraction and flexible structures, significant disorder was observed in the structure of (S)-**1**, especially within the flexible phenylalanine residues (Phe) and peptide bonds. To obtain a chemically reasonable structural model, a combination of restraints and constraints was applied during the structure refinement. Specifically, FLAT constraints were used to maintain the planarity of the benzene rings in the three Phe moieties; DELU and SIMU restraints were employed to reduce the unrealistic thermal motion anisotropy of disordered atom in the framework; DFIX restraints preserved rational bond lengths of flexible alkyl chains and peptide bonds; and RIGU constraints restricted excessive geometric distortions of the coordination sphere. Collectively, these treatments effectively mitigated the uncertainties induced by weak diffraction and ligand flexibility, ensuring that the bond lengths and bond angles, including Zn-O bonds: 1.910~2.031 Å and O-Zn-O angles: 100.6-118.3°, fall within the typical ranges reported for related coordination compounds. Besides, the restraints and constraints were kept mild, thus avoiding artificial bias and ensuring the reliability of the final structural parameters. Additionally, the scattering contributions from highly disordered guest molecules within the structure of (*S*)-**1** were subtracted using the SQUEEZE routine in the PLATON software package[2]. The product of (*S*)-**1** was formulated as [Zn**L**·DMF] according to the single-crystal structure. Crystal data and details of the data collection are given in the Table as follows, CCDC number 2500352 contains the supplementary crystallographic data for this paper. These data can be obtained free of charge via www.ccdc.cam.ac.uk/data_request/cif. Elemental analysis for (*S*)-**1**: Calcd. Anal. (%): C, 59.51; H,

4.74; N, 7.12. Found (%): C, 57.152; H, 5.527; N, 6.491. The deviations between these calculated values and experimental measurements are largely attributed to the inherent difficulty in accurately quantifying guest molecules encapsulated within its pores.

Dye adsorption procedure: Prior to dye adsorption, the chiral crystals of **1** were activated via Soxhlet extraction using methanol and acetone to exchange the DMF molecules within its pores. Subsequently, the solvent-exchanged crystals of **1** were soaked in 1.0 mM ethanol solutions of methylene blue (MB, with a size of 1.5×0.8×0.4 nm), crystal violet (CV, with a size of 1.4×1.5×0.6 nm), rhodamine B (RhB, with a size of 1.8×1.3×0.8 nm), and Evans blue (EB with a size of 2.9×1.3×0.9 nm), respectively. In the MB, CV, and RhB solutions, the initially colorless crystals of **1** gradually turn to blue, purple, and red over time, corresponding to the color of the respective dyes (Fig. S7), whereas almost no color change was observed for the crystals in the EB solution. The adsorption experiment, monitored by UV-Vis, indicated that **1** can uptake about 83%, 72%, and 69% of the total dye molecules for MB, CV, and RhB, respectively, at the adsorption equilibrium of twelve hours (Fig. S7). On the contrary, **1** exhibits almost no adsorption of Evans blue (EB with a size of 2.9×1.3×0.9 nm) whose molecule size exceeds its pore aperture.

Enantioseparation procedure: Before enantioseparation, the as-synthesized crystals of (S)-**1** were first activated by sequential solvent exchange with methanol and acetone via a Soxhlet extractor at 100 °C for 12 hours. Then the solvent-exchanged crystals were immersed in an acetone solution of indicated racemic analyte for 6 h at room temperature. Subsequently, the chiral solid adsorbent (*S*)-**1** was collected by filtration, rinsed with fresh solvent for serval times to remove off unadsorbed and weakly bound chiral substrate residues, and the encapsulated enantiomers were extracted by soaking the crystals in dichloromethane. The enantiomeric purity of the extracted chiral guests was determined by HPLC (Fig. S9, 12-19 for HPLC spectra). During the cyclic enantioseparation of styrene oxide (SO), the same batch of (*S*)-**1** crystals can be easily recovered by filtration, and repeatedly reused for five consecutive runs. The ee value of desorbed SO remained at ca. 99% for each run, without the deterioration of enantioselectivity (Fig. S13). Notably, substrate residues on chiral MOFs can compromise enantioselective separation performance to a certain extent. To eliminate this potential adverse effect, the recovered (*S*)-**1** crystals were subjected to thorough washing, desorption and solvent-exchange, which ensures the complete removal of the weakly bound substrate residues on the material.

Dynamic enantiosorption procedure: The dynamic enantiosorption was studied by immersing the same batch of (*S*)-**1** crystals (50 mg) in 1.5 mL acetone solutions containing the respective substrates (SO, GPE, and GTE, all at 1.0 mM). Then, the enantioselective recognition and adsorption performance was assessed by tracking the time-dependent changes in ee values and peak areas of the supernatant using HPLC. Each dynamic enantiosorption experiment was conducted in triplicate, confirming the reproducibility of the enantioselective behavior. (see Fig. S10 for HPLC spectra). The adsorption capacity of (*S*)-**1** for optically pure GTE isomers was determined at 25 °C and 35 °C. Following immersion of the crystals in (*S*)-GTE and (*R*)-GTE isomer solutions of known concentration, respectively. The adsorption amount was determined by

monitoring the change in solution concentration using UV-Vis spectroscopy. The adsorption capacity of (*S*)-**1** to enantiopure GTE isomers is shown in Fig. 3.

Fabrication of (*S*)-1-based MMM: According to the reported protocol, the (*S*)-**1**-based MMM was prepared by the solution casting method. PVDF (100 mg) and grinded (*S*)-**1** (20 mg) were placed in a vial containing 2 mL of *N*, *N*'-dimethylacetamide (DMAc). The resulting mixture was stirred for 2 h and sonicated for 2 h to give the casting solution. After that, the casting solution was poured onto a flat glass surface and cast into a membrane using a casting blade. The membrane was dried at 80 °C for 24 h. Finally, the membrane was cooled in air and peeled from the glass plate to obtain a (*S*)-**1**-based MMM.

2. Synthesis of chiral ligand (*S*)-H_3L and chiral MOF (*S*)-1.

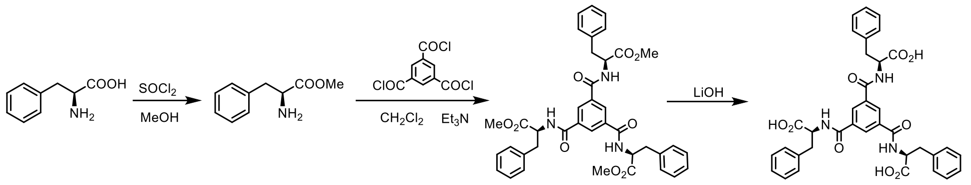

Synthesis of (*S*)-H_3**L**: The chiral ligand (*S*)-H_3**L** was synthesized from optical pure *L*-phenylalanine according to the above reaction routine. *L*-phenylalanine (5 g, 30.0 mmol) was dissolved in methanol (40 mL), followed by the dropwise addition of thionyl chloride (5 mL). The reaction mixture was stirred at room temperature for 24 h, then treated with 2 M NaOH to adjust the pH to approximately 9. The resulting mixture was extracted with ethyl acetate (3 × 30 mL), then the combined organic extracts were dried over Na_2SO_4 and concentrated under reduced pressure, affording the crude product of *L*-phenylalanine methyl ester (5.12 g, 94.4%). The obtained *L*-phenylalanine methyl ester (5.12 g, 28.6 mmol) was dissolved in dichloromethane (40 mL), followed by the dropwise addition of 1,3,5-benzenetricarbonyl trichloride (2.30 g, 8.67 mmol) and Et_3N (12.0 mL), respectively. The resulting reaction mixture was stirred at room temperature for an additional 4 h. After that, the mixture was washed with water (3×20 mL), 2M HCl (3×20 mL), and a saturated $NaHCO_3$ solution (3×20 mL), respectively. Subsequently, the organic phase was dried over Na_2SO_4 and concentrated under reduced pressure, affording methylated chiral ligand, **L**-Me_3 (ca. 5.97 g), with a yield of ca. 93.7%. The hydrolysis of **L**-Me_3 (5.97 g) was carried out in a mixed alkaline solution of $LiOH{\cdot}H_2O$ (0.97 g) in THF/H_2O (24 mL/6 mL) at 100 °C for 8 h. The resulting mixture was acidified with 2 M HCl and filleted. The crude product was purified by flash chromatography on silica gel to give the chiral ligand (*S*)-H_3**L** (4.73 g) in a yield of ca. 89.5%. (*R*)-H_3**L** was synthesized using the identical procedure, but employing *D*-phenylalanine as the chiral source.

Synthesis of (*S*)-**1:** A mixture of $Zn(NO_3)_2{\cdot}6H_2O$ (11.9 mg, 0.04 mmol) and (*S*)-H_3**L** (26 mg, 0.04 mmol) (1:1 molar ratio) in 10 mL mixture solution of DMF/EtOH/H_2O (1:1:1 volume ratio) was heated at 65 °C for 72 hours, colorless hexagonal crystals, (*S*)-**1**, can be obtained with a good yield of ca. 65% based on (*S*)-H_3**L**. (*R*)-**1** was prepared using an identical procedure, but employing (*R*)-H_3**L** as the chiral ligand.

3. Table S1. Crystal data and structure refinement for (*S*)-1.

Identification code	(*S*)-**1**
Empirical formula	$C_{39}H_{37}N_4O_{10}Zn$
Formula weight	787.09
Temperature (K)	100
Wavelength (Å)	0.65251
Crystal system, space group	Cubic, $I2_13$
Unit cell dimensions	$a = b = c$ = 38.246(6) Å $\alpha = \beta = \gamma$ = 90 °
Volume	55944(3) Å^3
Z, Calculated density	24, 0.561 mg/m^3
Absorption coefficient	0.23 mm^{-1}
F(000)	9816
θ range for data collection (º)	1.383 to 16.511
Limiting indices	-33<=h<=33, -33<=k<=33, -33<=l<=33
Reflections collected Independent reflections	125195 / 6464 [R(int) = 0.0936]
Completeness to theta Refinement method	99.6 %
Refinement method	Full-matrix least-squares on F^2
Goodness-of-fit on F^2	1.036
Final R indices [I>2sigma(I)]	R1 = 0.0543, wR2 = 0.1419
R indices (all data)	R1 = 0.0631, wR2 = 0.1522

4. Table S2. Selected bond lengths [Å] and angles [º] for (*S*)-1.

Bond Lengths	
Zn(1)-O(1)#1	1.964(6)
Zn(1)-O(10)	2.031(7)
Zn(1)-O(4)#2	1.979(7)
Zn(1)-O(6)	1.910(1)
**Bond Angles**	
O(1)#1-Zn(1)-O(10)	118.3(3)
O(1)#1-Zn(1)-O(4)#2	120.6(2)
O(4)#2-Zn(1)-O(10)	100.9(3)
O(6)-Zn(1)-O(1)#1	100.6(4)
O(6)-Zn(1)-O(10)	105.5(5)
O(6)-Zn(1)-O(4)#2	110.1(3)

Symmetry transformations used to generate equivalent atoms:
#1 y, z, x #2 -x+1, -y+1/2, z

5. Figures S1-S2. X-ray crystallographic structure of (*S*)-1.

Fig. S1. The chiral triangular channel in (S)-**1** (space-filling model) along the [111] direction.

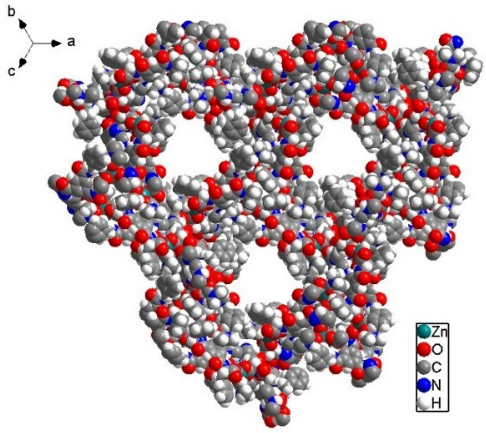

Fig. S2. The irregular triangular aperture of the chiral inner cavity.

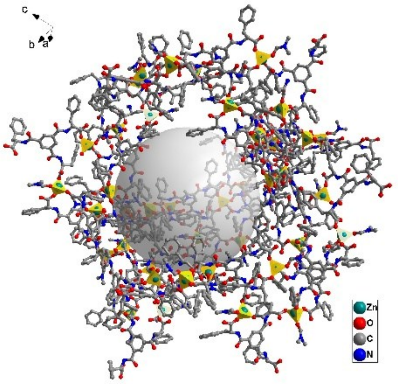

6. Figure S3. PXRD patterns of (*S*)-1.

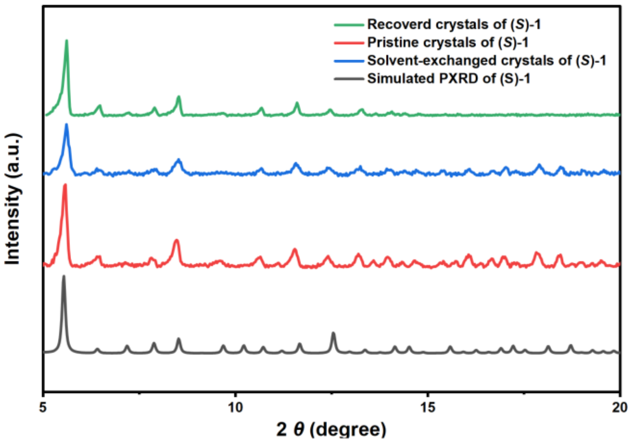

7. Figure S4. IR spectra of H_3L and (*S*)-1.

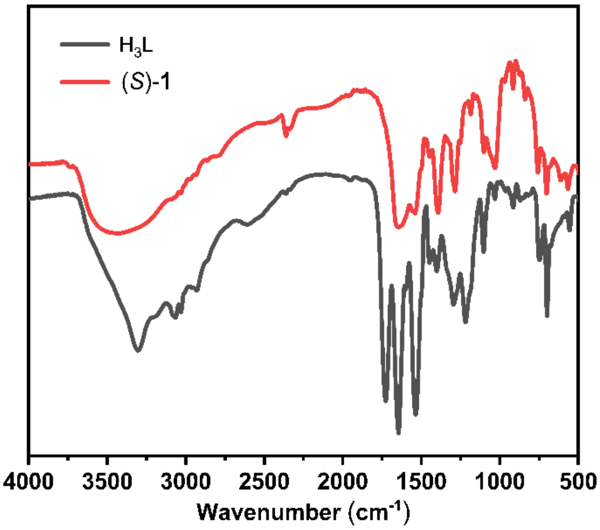

8. Figure S5. UV-Vis spectrum of H_3L and (*S*)-1.

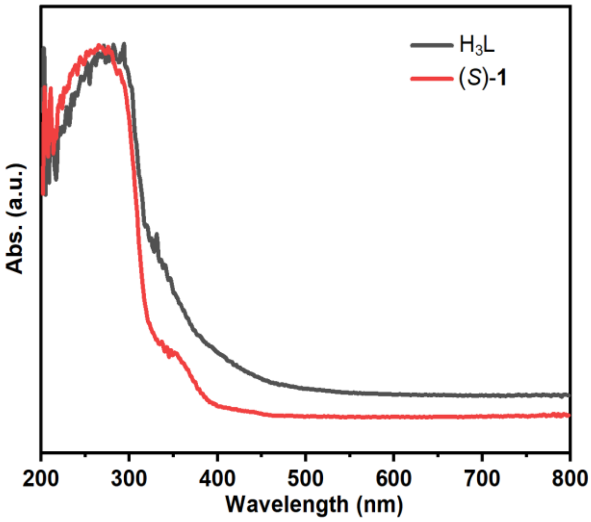

9. Figure S6. TGA curves of (*S*)-1.

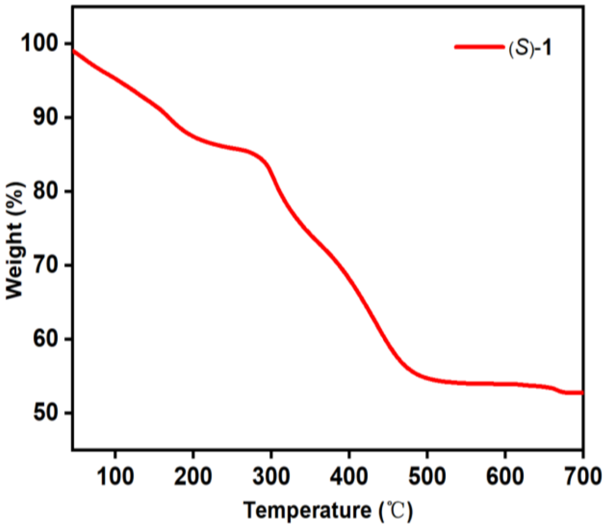

10. Figure S7. Dynamic adsorption of dye molecules by (*S*)-1 monitored by UV-Vis.

The 100 mg solvent-exchanged crystals of (*S*)-**1** were immersed in methanol solution of 1.0 mM MB, CV and RhB, respectively. Then the dye content in solution was monitored by UV-Vis at different time periods.

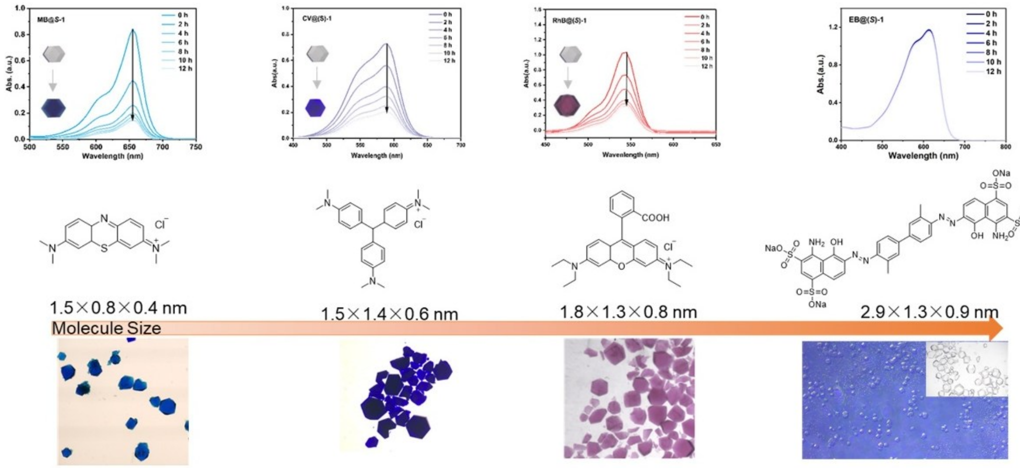

11. Figure S8. XPS data of (*S*)-1.

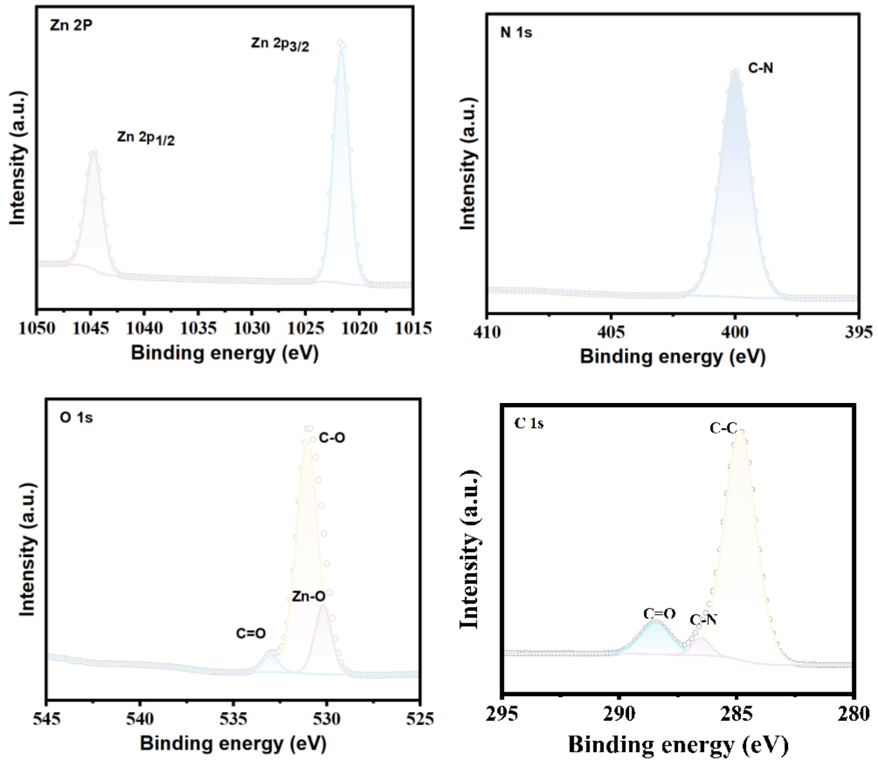

12. Figure S9. HPLC results from the solvent screen (in Table 1).

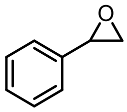

Chiral HPLC analysis: Daicel Chiralce IC: hexane/*i*-PrOH = 95/5, flow rate = 1 mL/min, 220 nm.

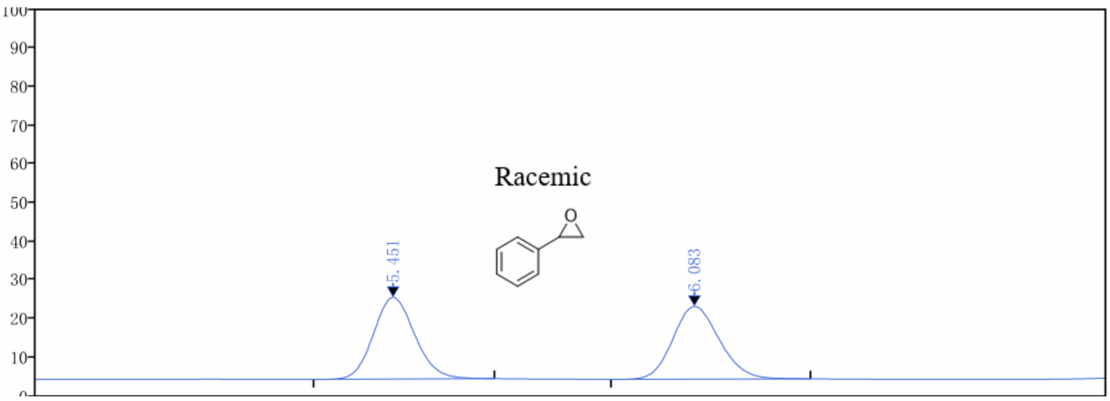

Serial Number	Type	Retention Time [min]	Peak Area	Area %
1	MM	5.451	1597.55	50.02
2	MM	6.083	1596.26	49.98
The Total			3193.81	

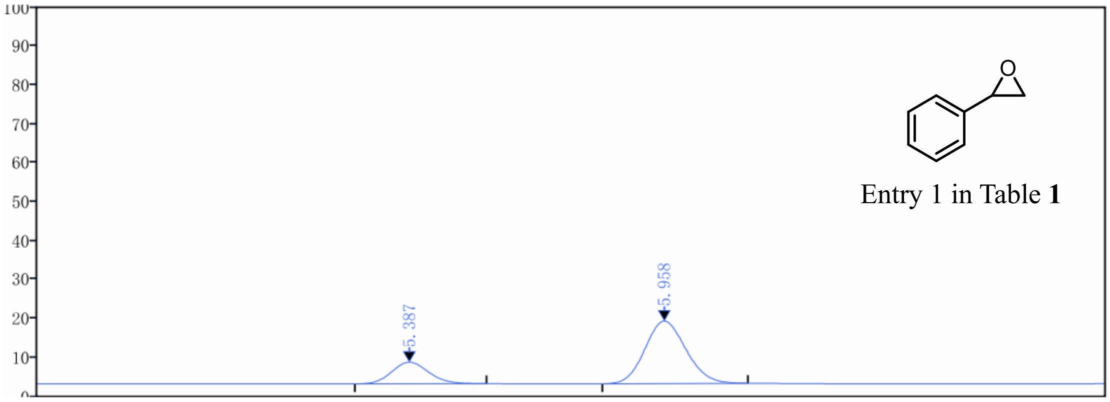

Serial Number	Type	Retention Time [min]	Peak Area	Area %
1	BM	5.387	476.22	23.72
2	MM	5.958	1531.38	76.28
The Total			2007.60	

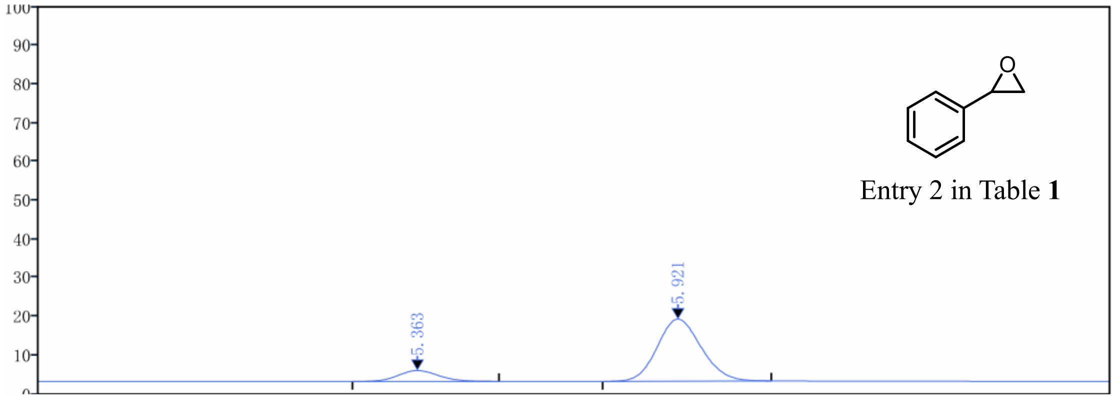

Serial Number	Type	Retention Time [min]	Peak Area	Area %
1	MM	5.363	68.90	13.90
2	MM	5.921	426.81	86.10
The Total			495.71	

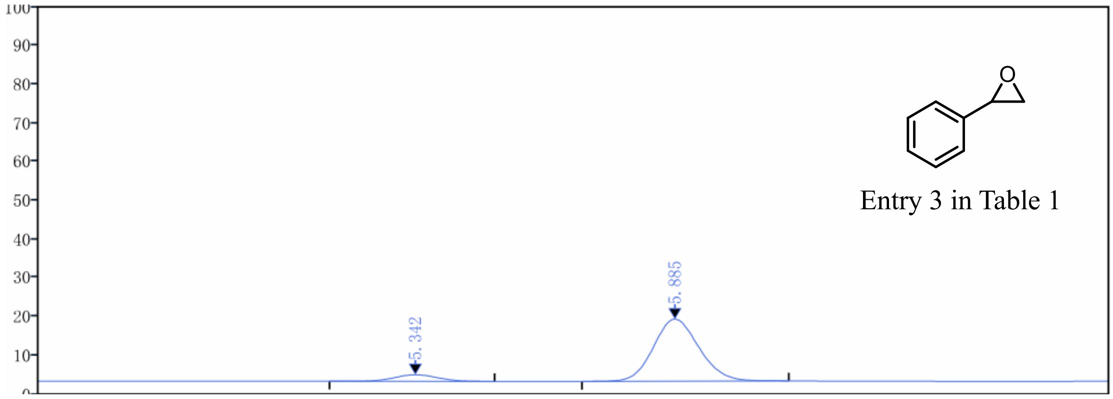

Serial Number	Type	Retention Time [min]	Peak Area	Area %
1	MM	5.342	48.26	8.94
2	MM	5.885	491.80	91.06
The Total			540.06	

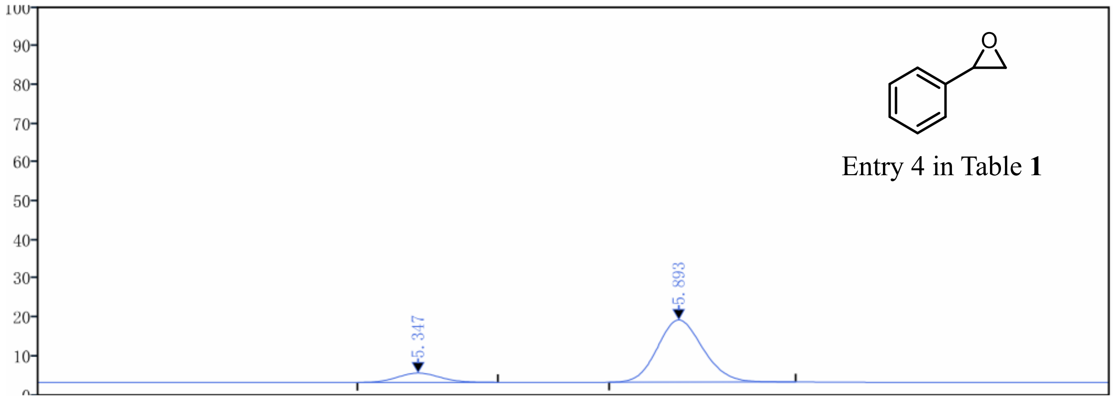

Serial Number	Type	Retention Time [min]	Peak Area	Area %
1	MM	5.347	117.71	12.27
2	MM	5.893	841.69	87.73
The Total			959.40	

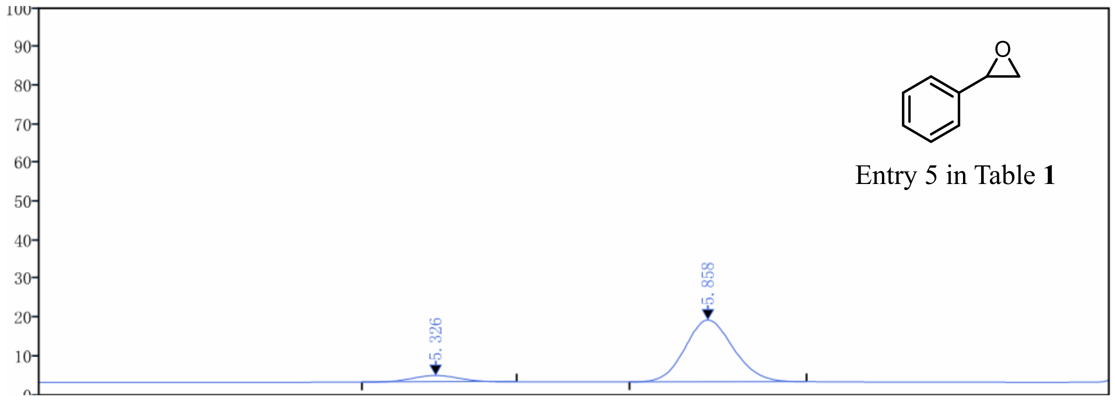

Serial Number	Type	Retention Time [min]	Peak Area	Area %
1	MM	5.326	54.08	7.81
2	MM	5.858	638.23	92.19
The Total			692.31	

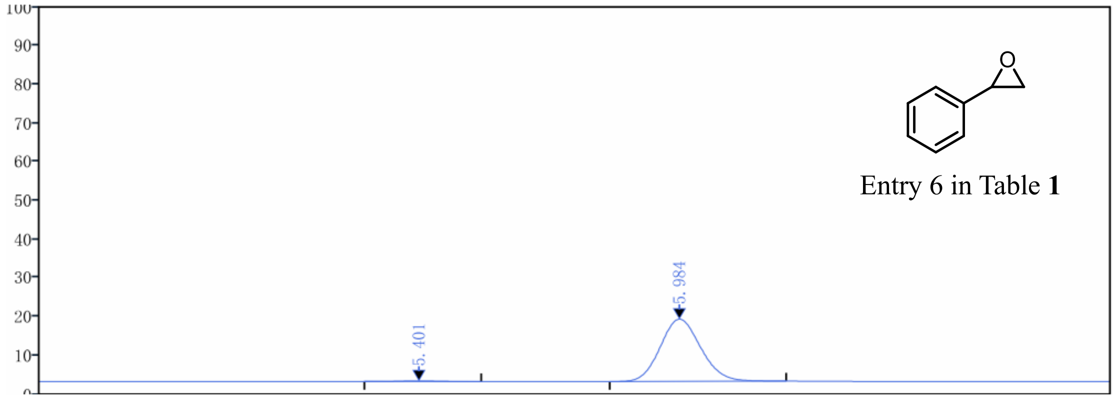

Serial Number	Type	Retention Time [min]	Peak Area	Area %
1	MM	5.401	6.63	0.50
2	MM	5.984	1329.45	99.50
The Total			1336.08	

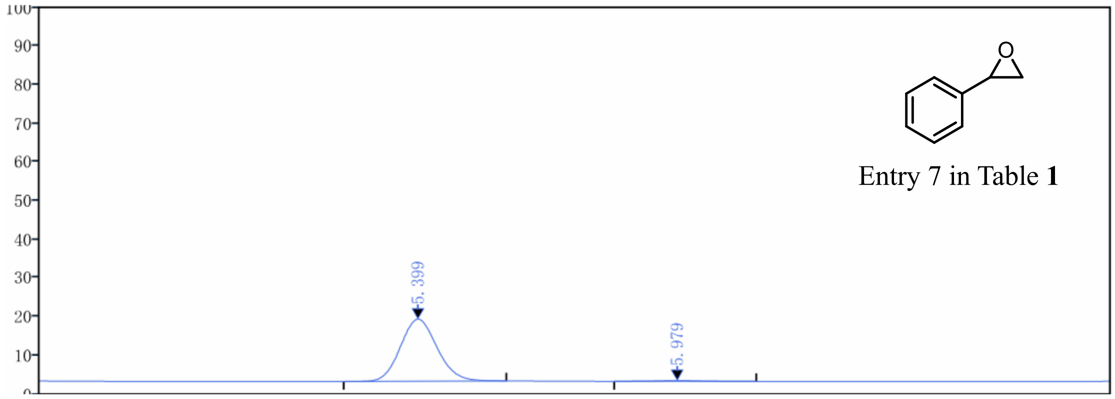

Serial Number	Type	Retention Time [min]	Peak Area	Area %
1	BM	5.399	1458.73	99.64
2	MM	5.979	5.24	0.36
The Total			1463.97	

13. Figure S10. HPLC analysis of dynamic enantiosorption by (S)-1.

13.1 Enantiosorption of SO by (*S*)-1.

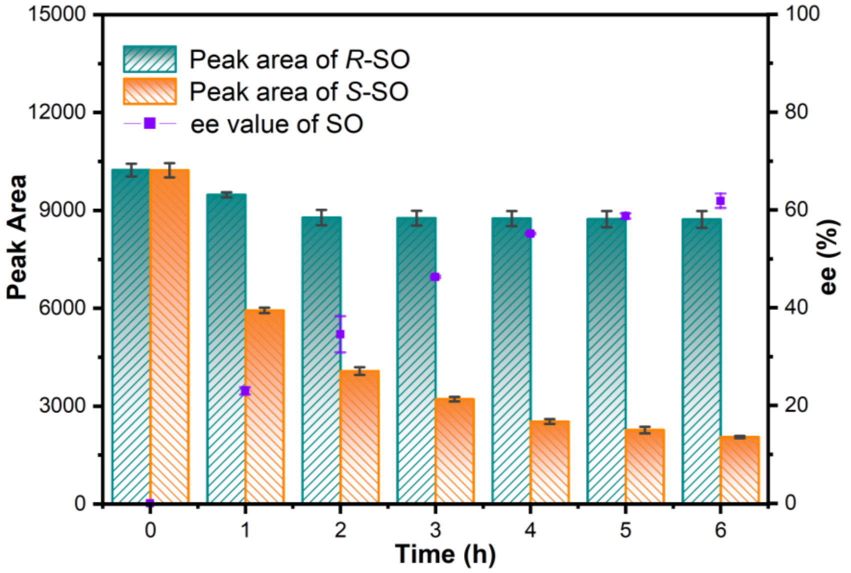

The HPLC spectra are listed as follows.

The first run of enantiosorption of SO by (*S*)-1

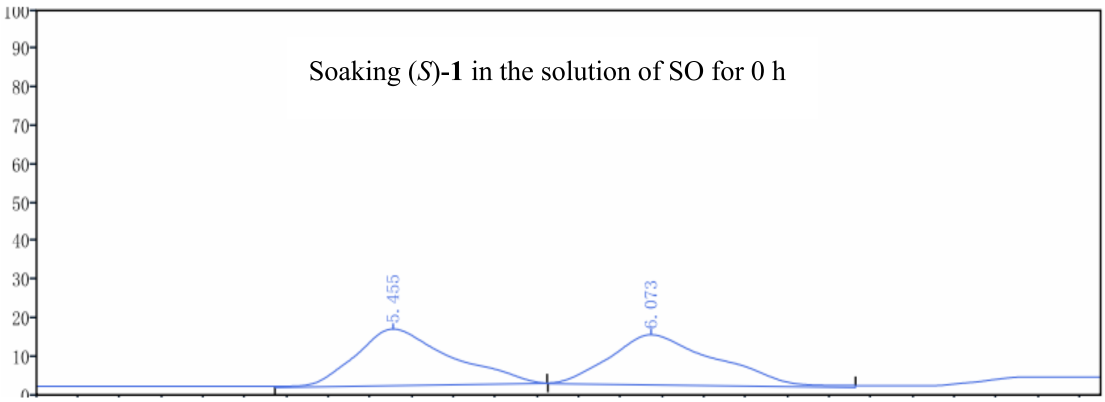

Serial Number	Type	Retention Time [min]	Peak Area	Area %
1	MM	5.455	10196.43	50.06
2	MM	6.073	10173.26	49.94
The Total			20369.69	

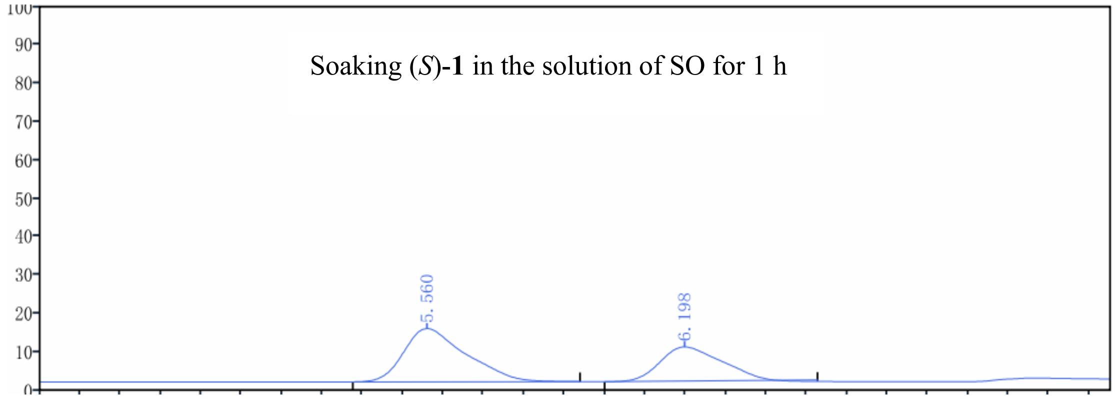

Serial Number	Type	Retention Time [min]	Peak Area	Area %
1	MM	5.560	9402.78	61.54
2	MM	6.198	5876.62	38.46
The Total			15279.40	

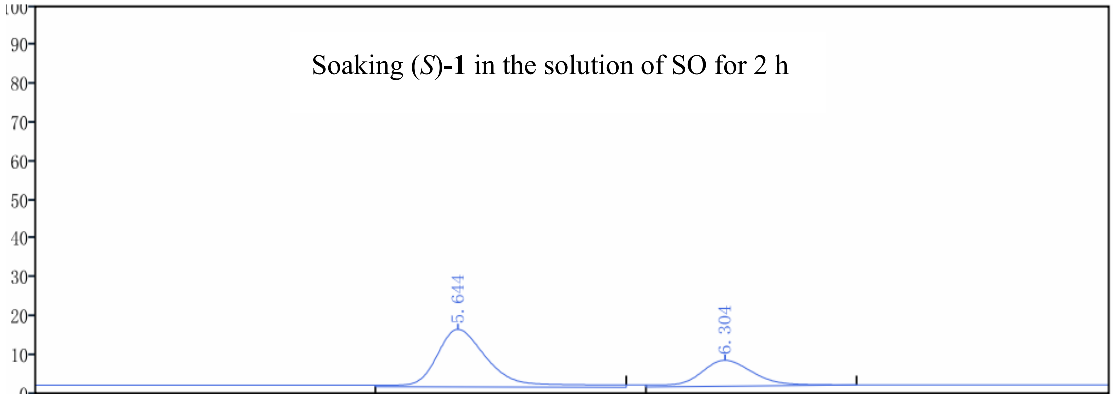

Serial Number	Type	Retention Time [min]	Peak Area	Area %
1	MM	5.644	8867.46	68.66
2	MM	6.304	4046.92	31.34
The Total			12914.38	

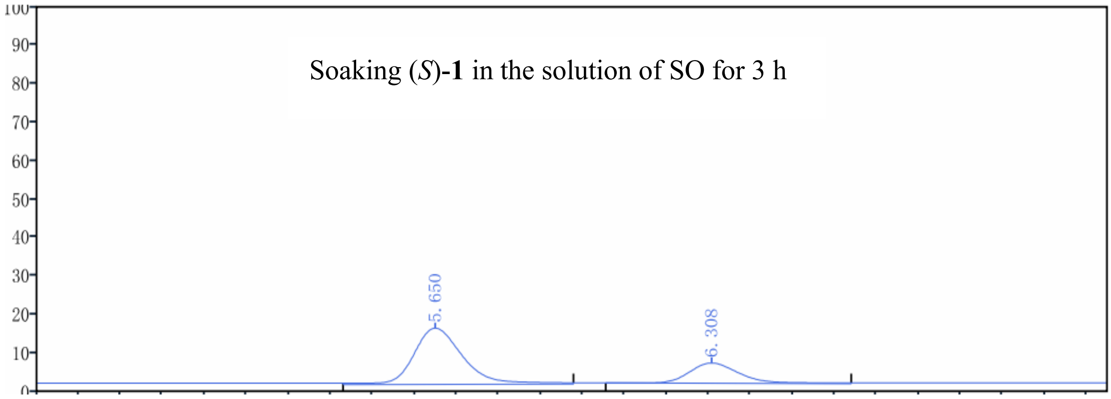

Serial Number	Type	Retention Time [min]	Peak Area	Area %
1	MM	5.650	8843.34	73.14
2	MM	6.308	3247.95	26.86
The Total			12091.29	

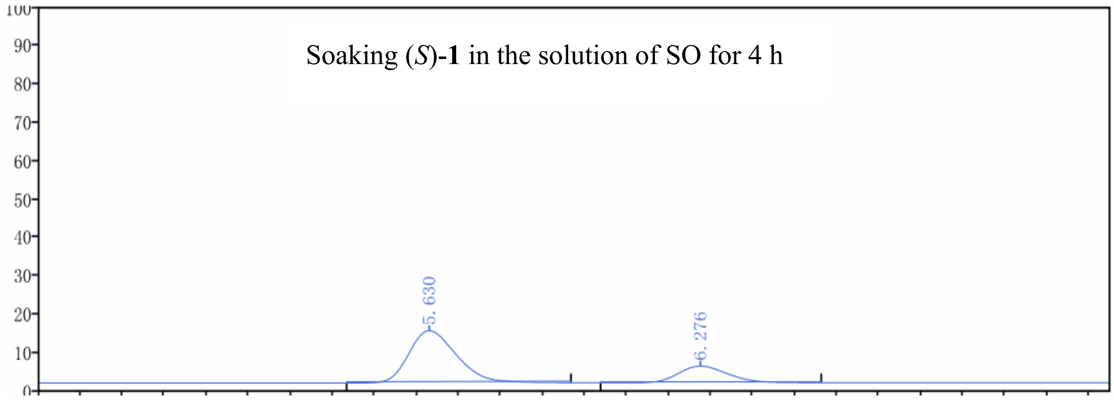

Serial Number	Type	Retention Time [min]	Peak Area	Area %
1	MM	5.630	8821.31	77.49
2	MM	6.276	2562.58	22.51
The Total			11383.89	

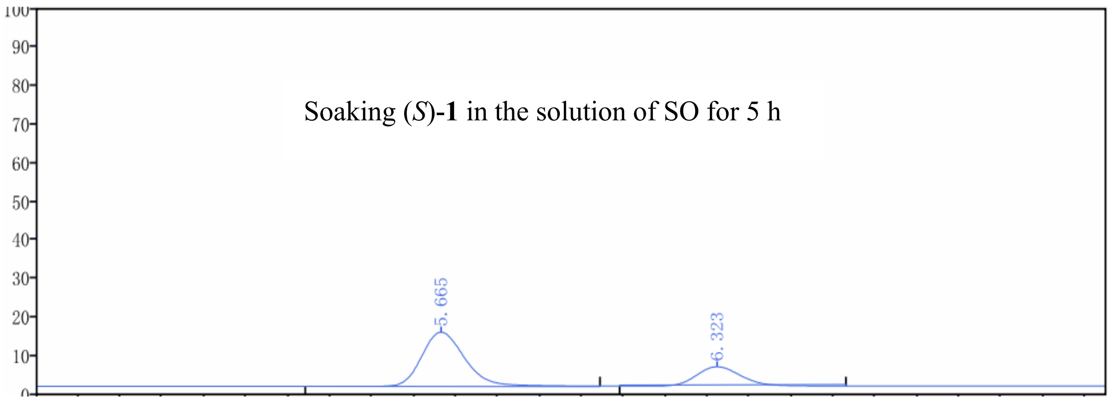

Serial Number	Type	Retention Time [min]	Peak Area	Area %
1	MM	5.665	8810.84	79.46
2	MM	6.323	2277.83	20.54
The Total			11088.67	

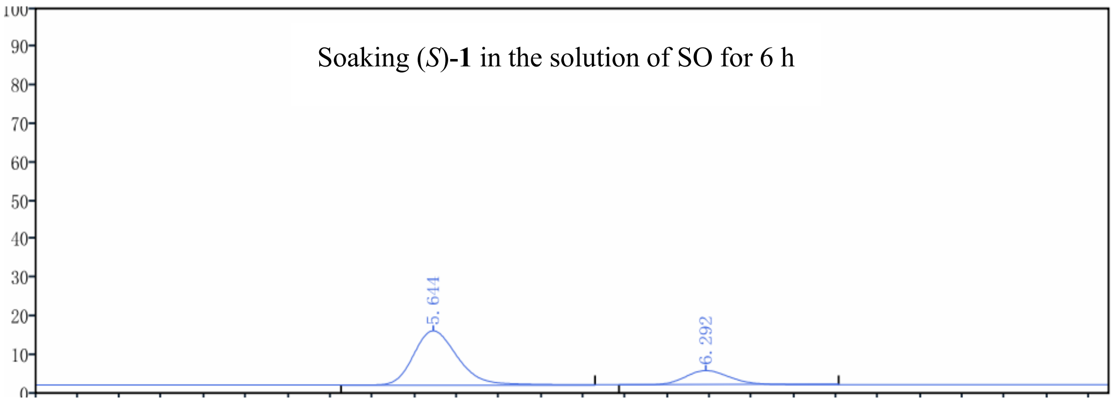

Serial Number	Type	Retention Time [min]	Peak Area	Area %
1	MM	5.644	8801.91	81.07
2	MM	6.292	2055.02	18.93
The Total			10856.93	

The second run of enantiosorption of SO by (*S*)-1

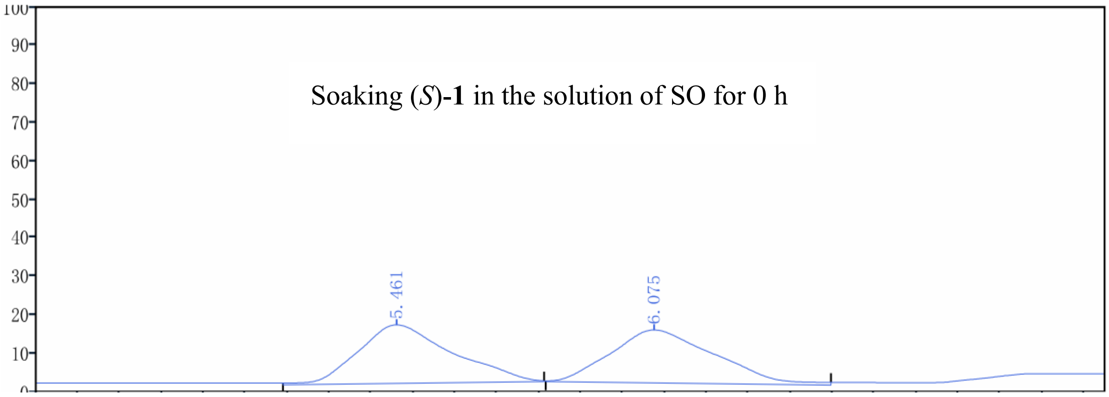

Serial Number	Type	Retention Time [min]	Peak Area	Area %
1	MM	5.461	10060.53	50.03
2	MM	6.075	10046.56	49.97
The Total			20107.09	

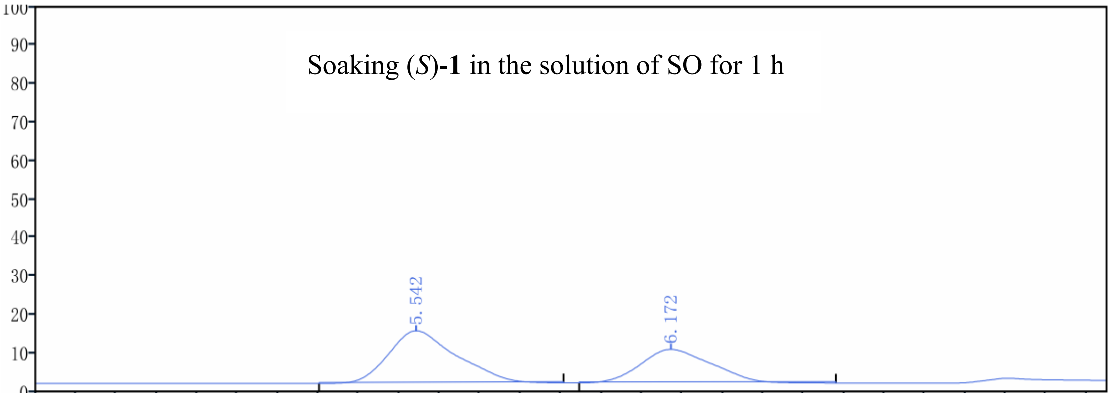

Serial Number	Type	Retention Time [min]	Peak Area	Area %
1	MM	5.542	9473.84	61.09
2	MM	6.172	6033.63	38.91
The Total			15507.47	

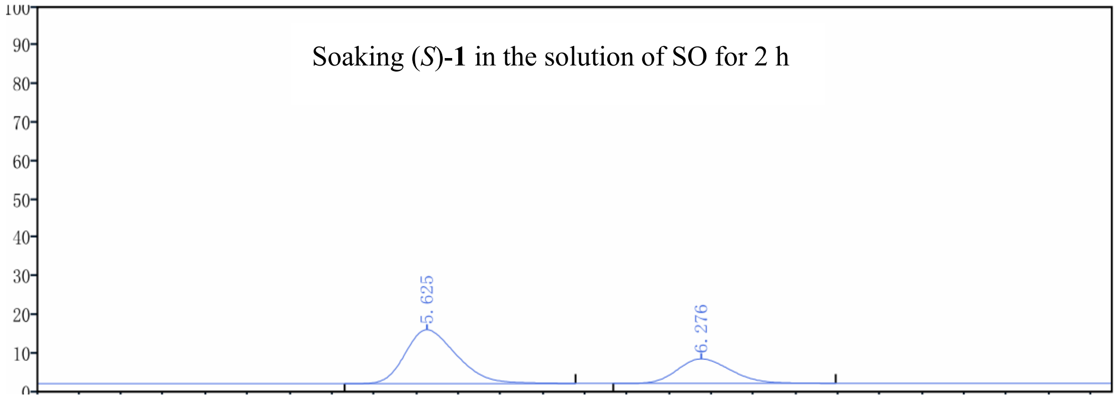

Serial Number	Type	Retention Time [min]	Peak Area	Area %
1	BM	5.625	8512.20	68.18
2	MM	6.276	3973.56	31.82
The Total			12485.76	

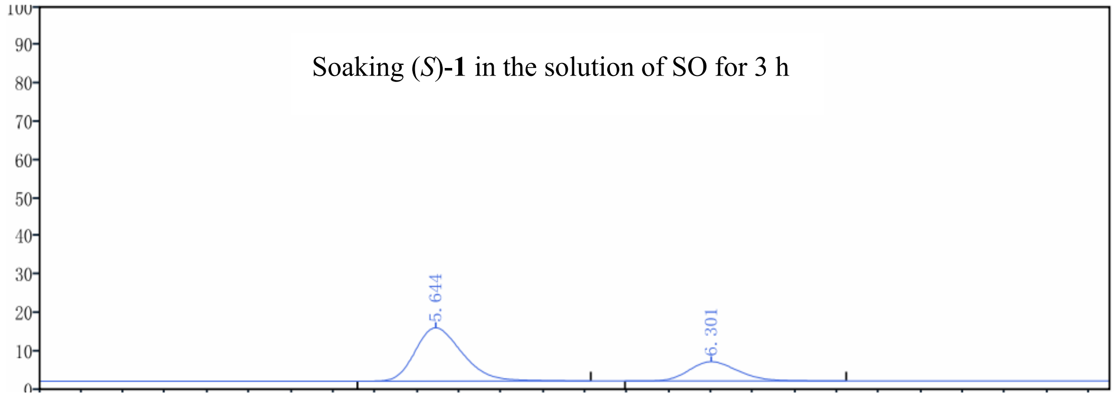

Serial Number	Type	Retention Time [min]	Peak Area	Area %
1	BM	5.644	8508.83	73.05
2	MM	6.301	3138.75	26.95
The Total			11647.58	

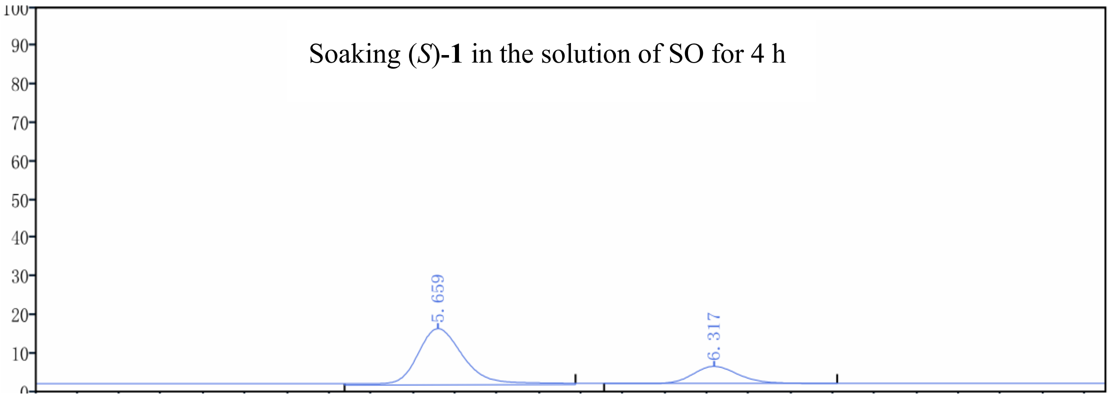

Serial Number	Type	Retention Time [min]	Peak Area	Area %
1	MM	5.659	8497.43	77.67
2	MM	6.317	2443.02	22.33
The Total			10940.45	

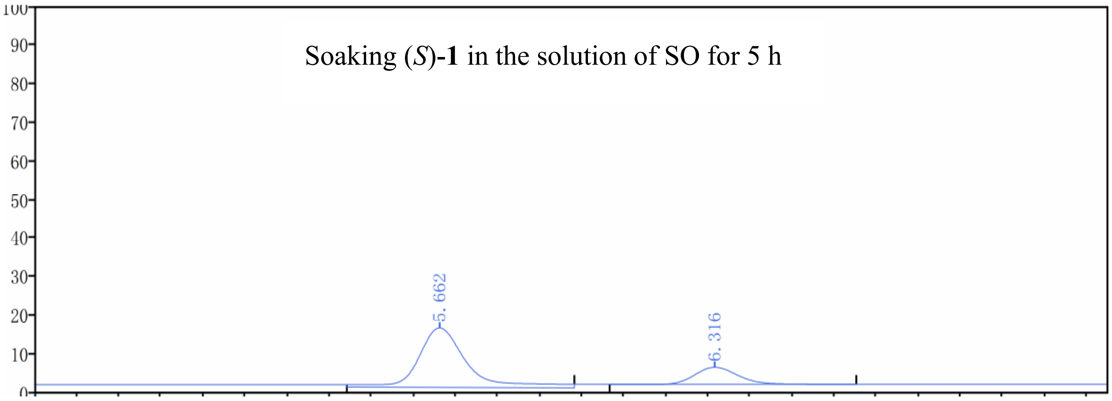

Serial Number	Type	Retention Time [min]	Peak Area	Area %
1	MM	5.662	8458.32	79.61
2	MM	6.316	2165.98	20.39
The Total			10624.30	

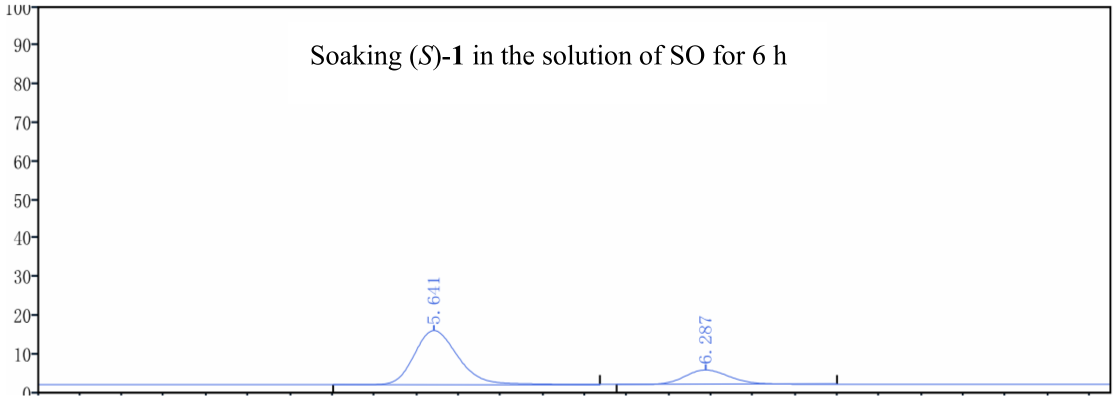

Serial Number	Type	Retention Time [min]	Peak Area	Area %
1	MM	5.641	8438.90	80.15
2	MM	6.287	2089.77	19.85
The Total			10528.67	

The third run of enantiosorption of SO by (*S*)-1

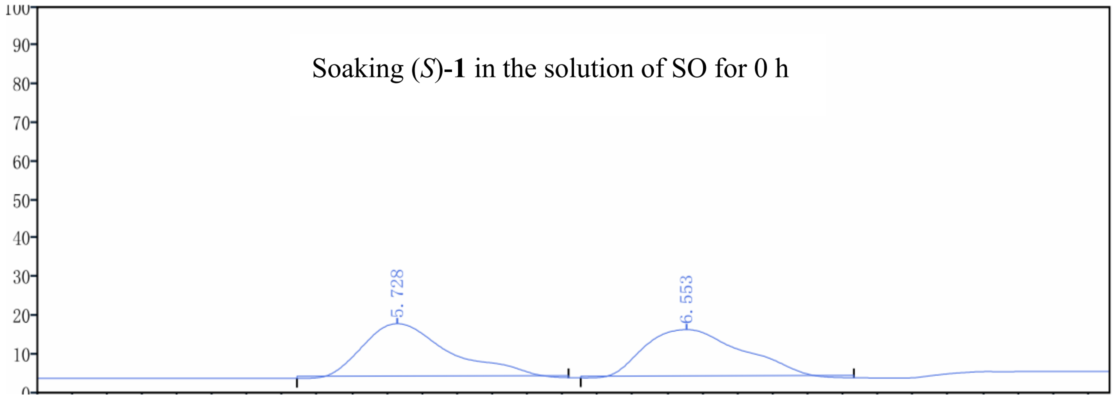

Serial Number	Type	Retention Time [min]	Peak Area	Area %
1	MM	5.728	10449.10	49.94
2	MM	6.553	10475.08	50.06
The Total			20924.18	

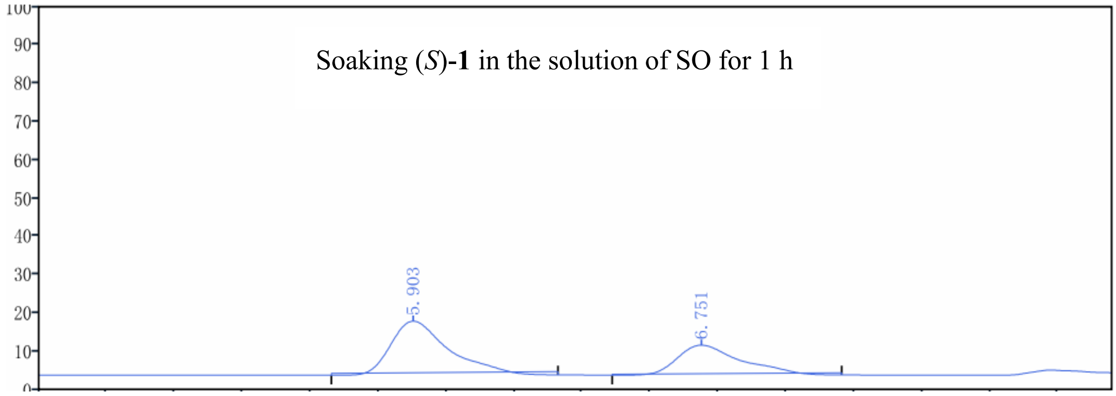

Serial Number	Type	Retention Time [min]	Peak Area	Area %
1	MM	5.903	9562.36	61.84
2	MM	6.751	5900.18	38.16
The Total			15462.54	

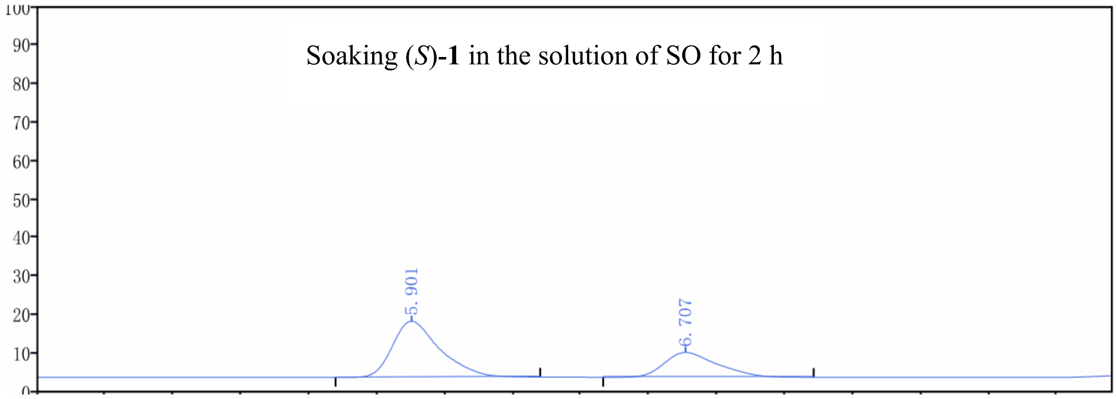

Serial Number	Type	Retention Time [min]	Peak Area	Area %
1	MM	5.901	8956.42	68.03
2	MM	6.707	4209.11	31.97
The Total			13165.53	

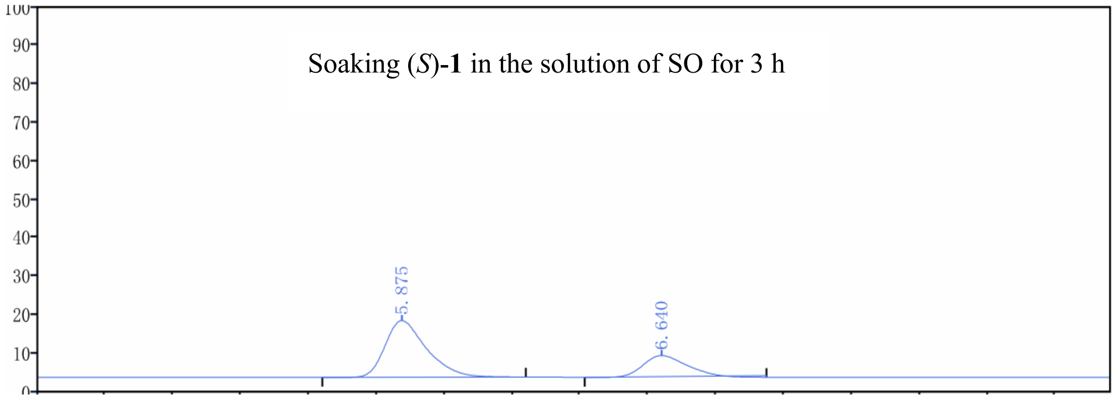

Serial Number	Type	Retention Time [min]	Peak Area	Area %
1	MM	5.875	8944.37	73.30
2	MM	6.640	3258.13	26.70
The Total			12202.50	

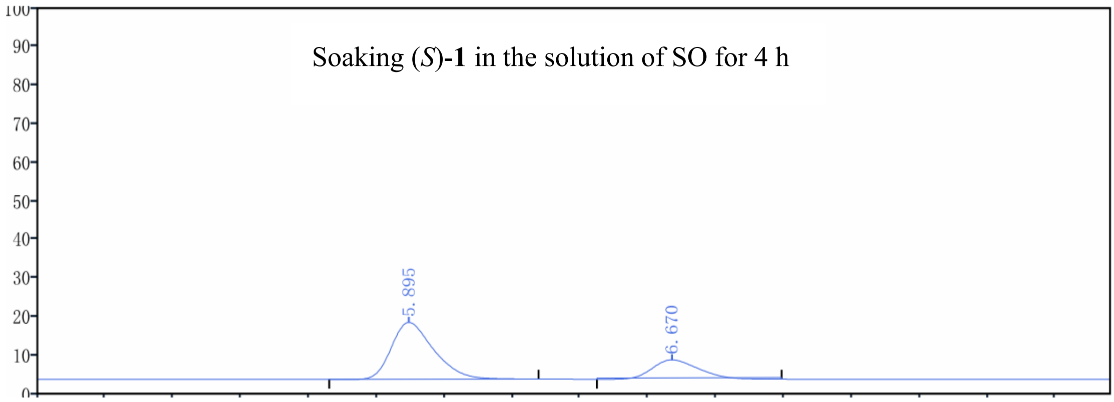

Serial Number	Type	Retention Time [min]	Peak Area	Area %
1	MM	5.895	8943.67	77.61
2	MM	6.670	2579.64	22.39
The Total			11523.31	

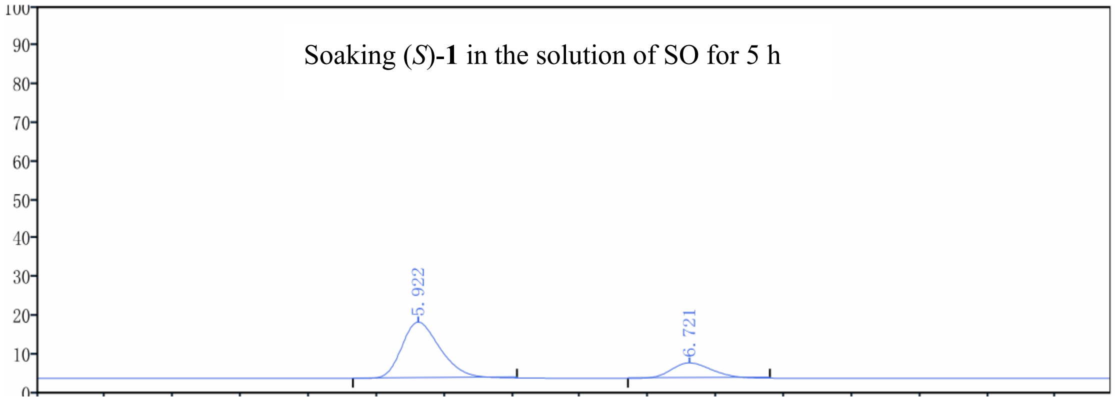

Serial Number	Type	Retention Time [min]	Peak Area	Area %
1	MM	5.922	8939.29	79.07
2	MM	6.721	2366.61	20.93
The Total			11305.90	

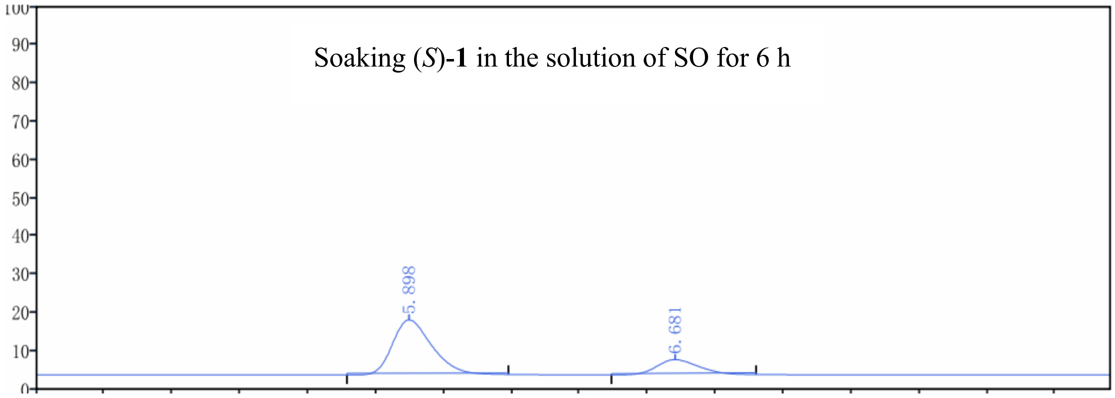

Serial Number	Type	Retention Time [min]	Peak Area	Area %
1	MM	5.898	8934.31	81.60
2	MM	6.681	2014.30	18.40
The Total			10948.61	

13.2 Enantiosorption of GPE by (*S*)-1.

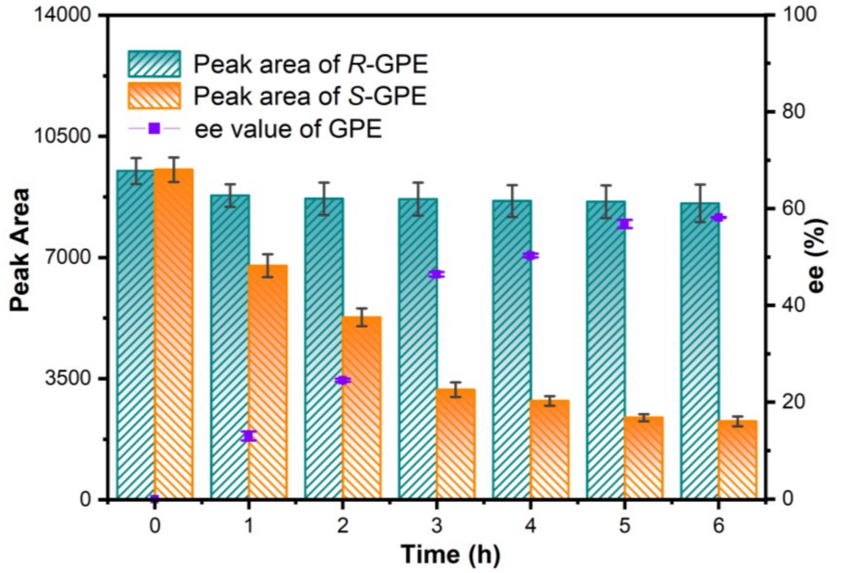

The HPLC spectra are listed as follows.

The first run of enantiosorption of GPE by (*S*)-1

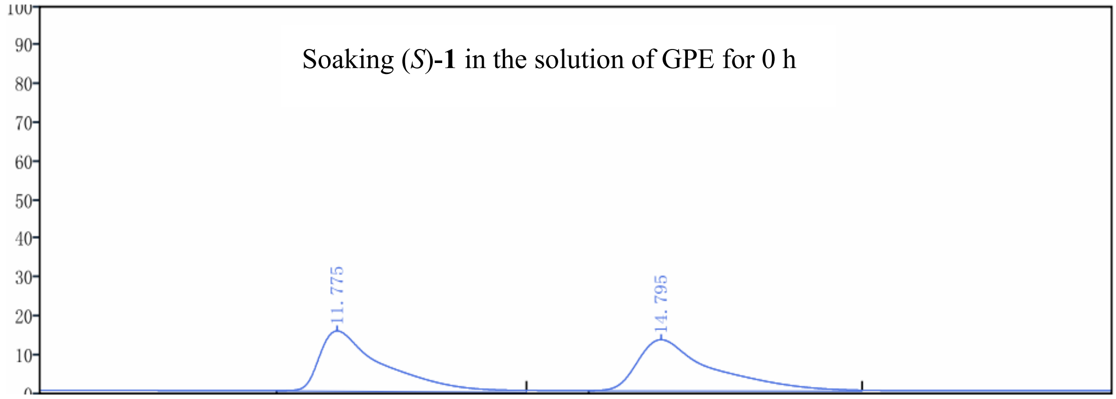

Serial Number	Type	Retention Time [min]	Peak Area	Area %
1	MM	11.775	9859.07	50.02
2	MM	14.795	9850.36	49.98
The Total			19709.43	

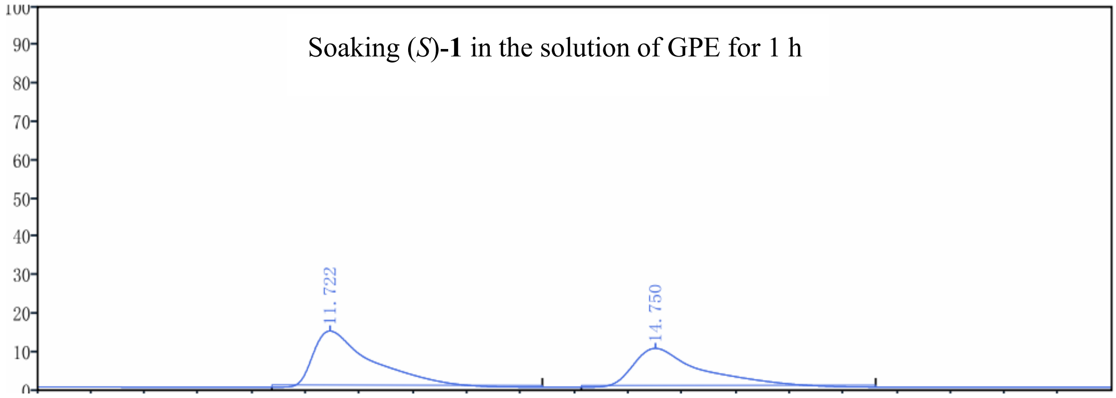

Serial Number	Type	Retention Time [min]	Peak Area	Area %
1	MM	11.722	8186.17	56.18
2	MM	14.750	6384.61	43.82
The Total			14570.78	

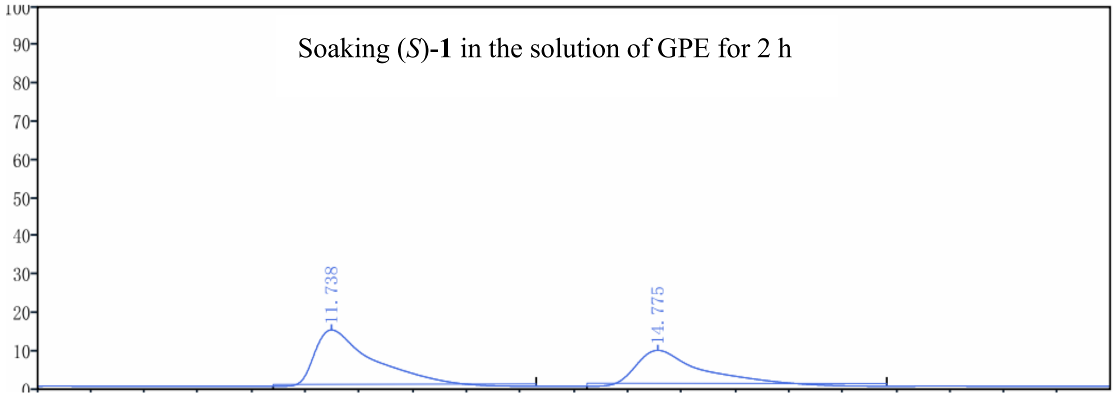

Serial Number	Type	Retention Time [min]	Peak Area	Area %
1	MM	11.738	8174.25	62.09
2	MM	14.775	4990.25	37.91
The Total			13164.50	

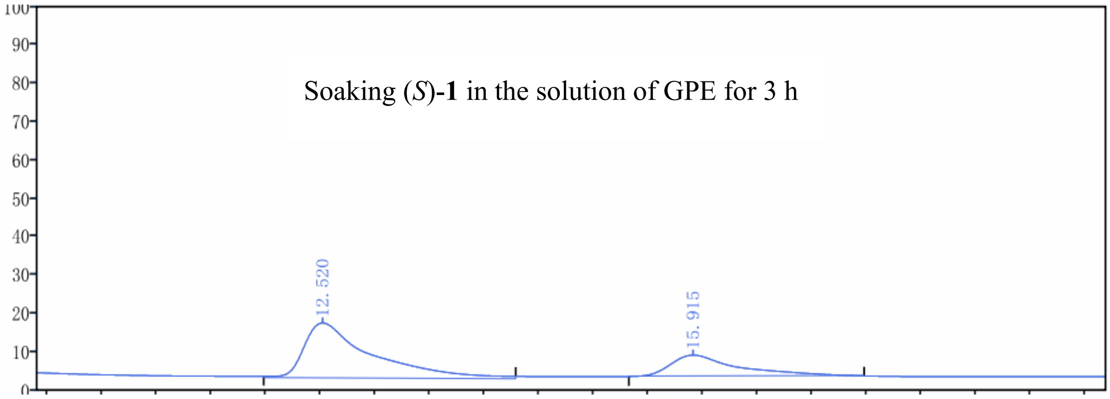

Serial Number	Type	Retention Time [min]	Peak Area	Area %
1	MM	12.520	8153.73	73.46
2	MM	15.915	2946.15	26.54
The Total			11099.88	

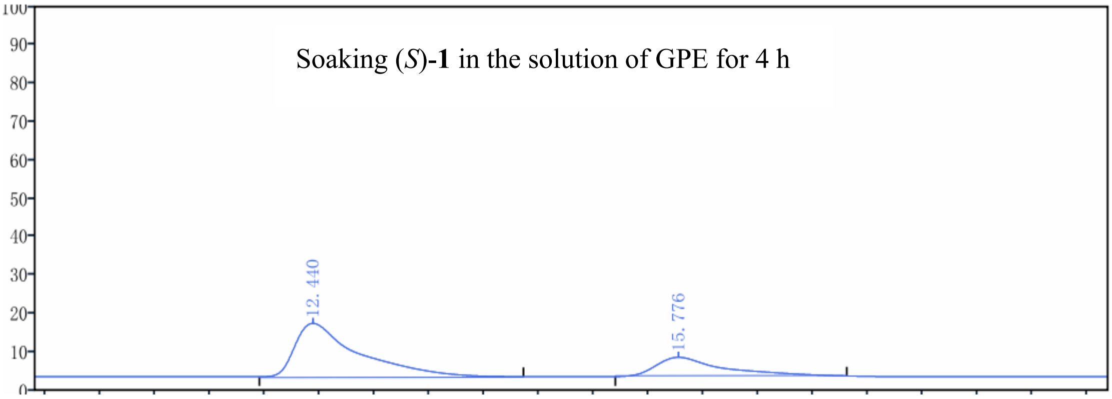

Serial Number	Type	Retention Time [min]	Peak Area	Area %
1	MM	12.440	8109.08	75.02
2	MM	15.776	2699.97	24.98
The Total			10809.05	

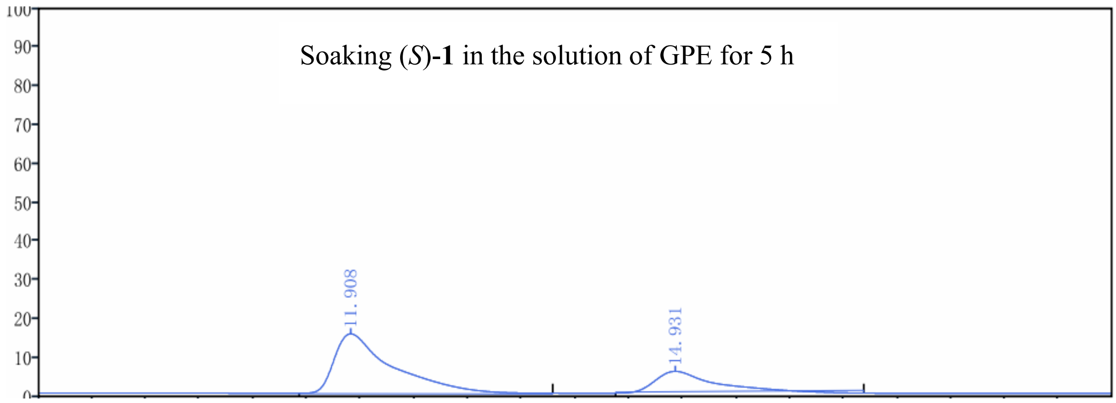

Serial Number	Type	Retention Time [min]	Peak Area	Area %
1	MM	11.908	8074.85	78.06
2	MM	14.931	2269.68	21.94
The Total			10344.53	

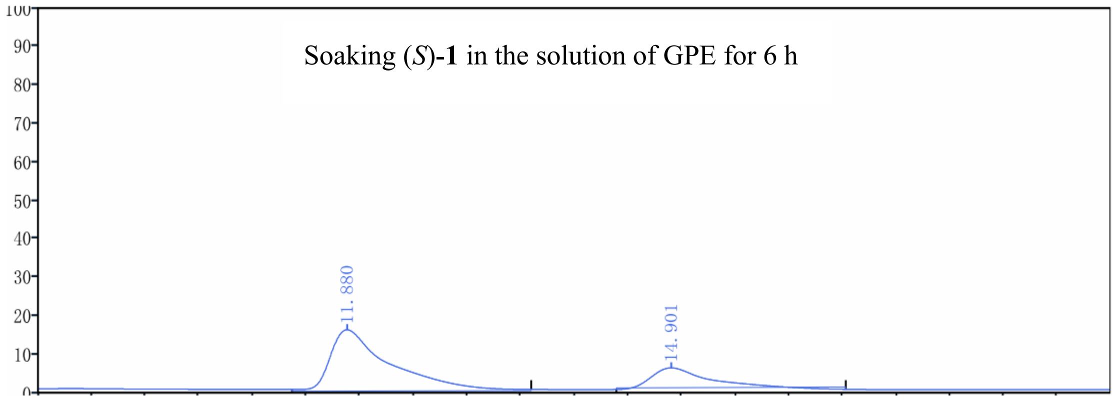

Serial Number	Type	Retention Time [min]	Peak Area	Area %
1	MM	11.880	7946.26	79.07
2	MM	14.901	2103.08	20.93
The Total			10049.34	

The second run of enantiosorption of GPE by (*S*)-1

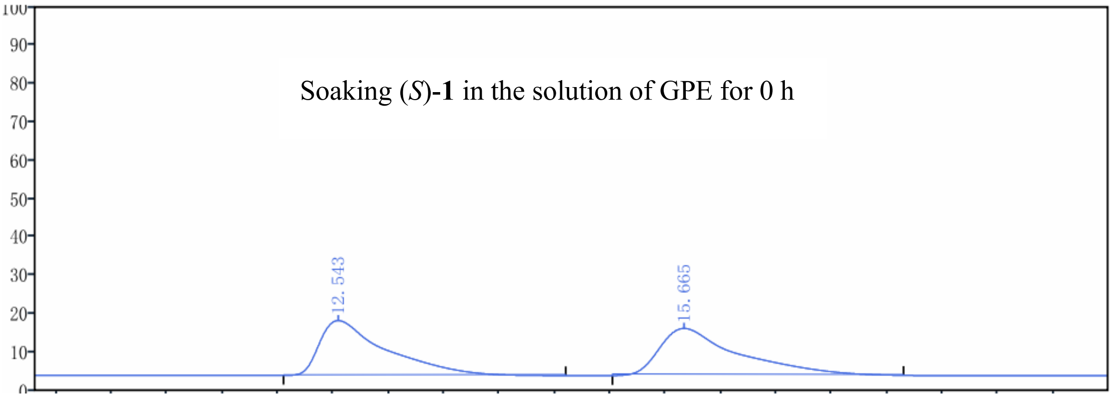

Serial Number	Type	Retention Time [min]	Peak Area	Area %
1	MM	12.543	9811.65	49.96
2	MM	15.665	9828.43	50.04
The Total			19640.08	

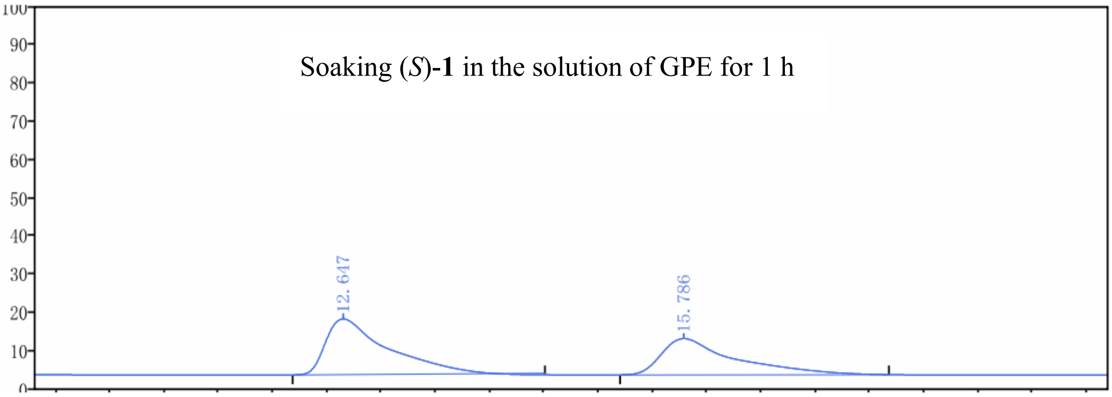

Serial Number	Type	Retention Time [min]	Peak Area	Area %
1	MM	12.647	8843.27	56.01
2	MM	15.786	6944.18	43.99
The Total			15787.45	

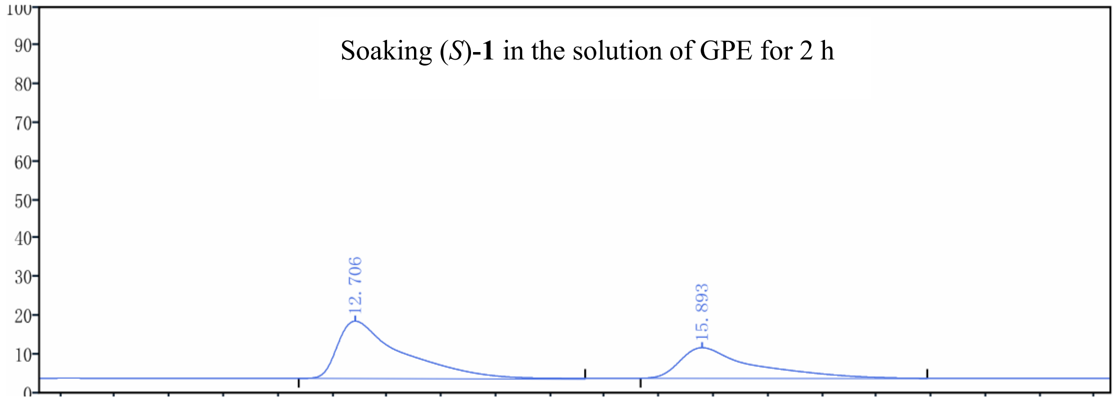

Serial Number	Type	Retention Time [min]	Peak Area	Area %
1	MM	12.706	8842.75	62.41
2	MM	15.893	5325.98	37.59
The Total			14168.73	

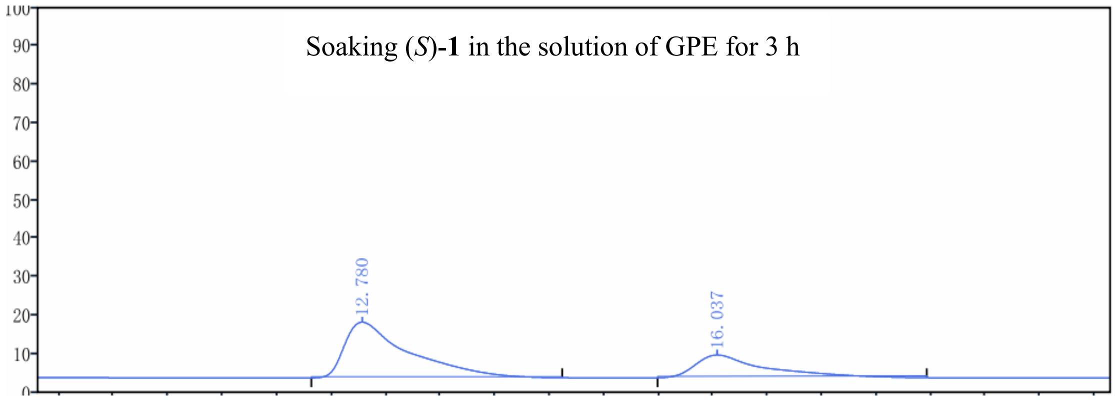

Serial Number	Type	Retention Time [min]	Peak Area	Area %
1	MM	12.780	8838.88	73.19
2	MM	16.037	3236.76	26.81
The Total			12073.64	

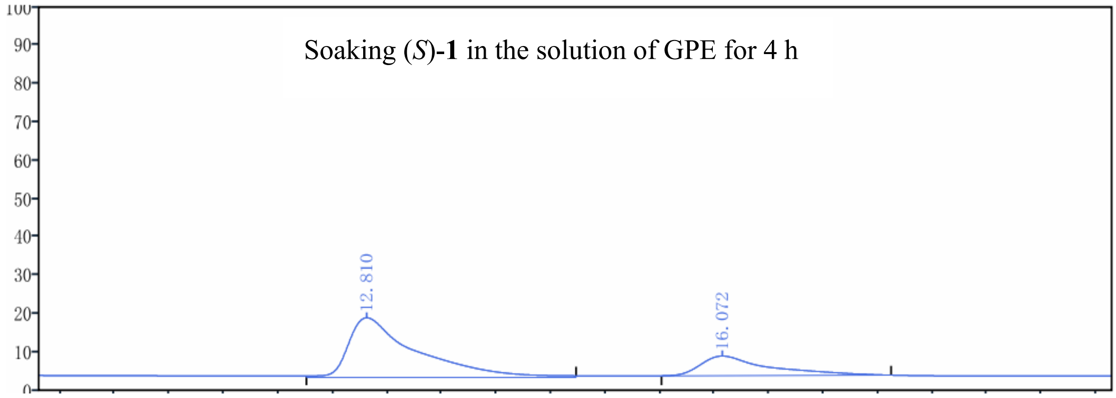

Serial Number	Type	Retention Time [min]	Peak Area	Area %
1	MM	12.810	8828.91	75.36
2	MM	16.072	2886.00	24.64
The Total			11714.91	

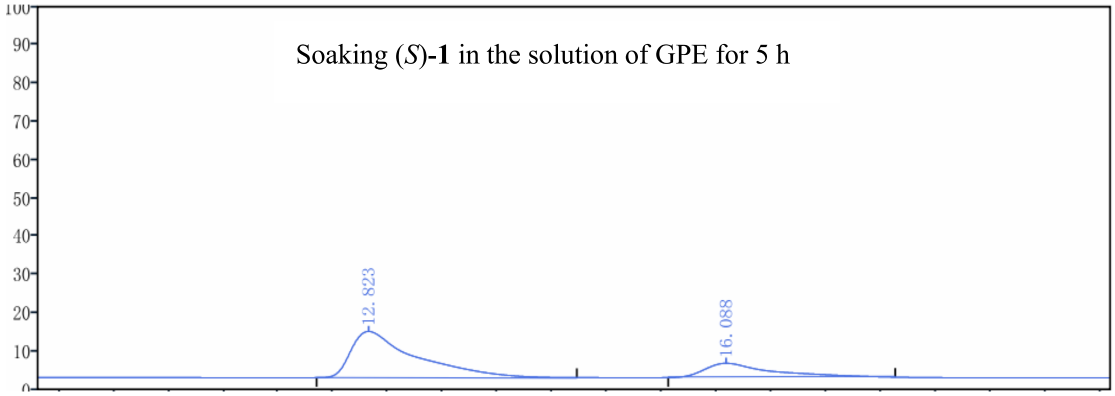

Serial Number	Type	Retention Time [min]	Peak Area	Area %
1	MM	12.823	8818.72	78.88
2	MM	16.088	2360.70	21.12
The Total			11179.42	

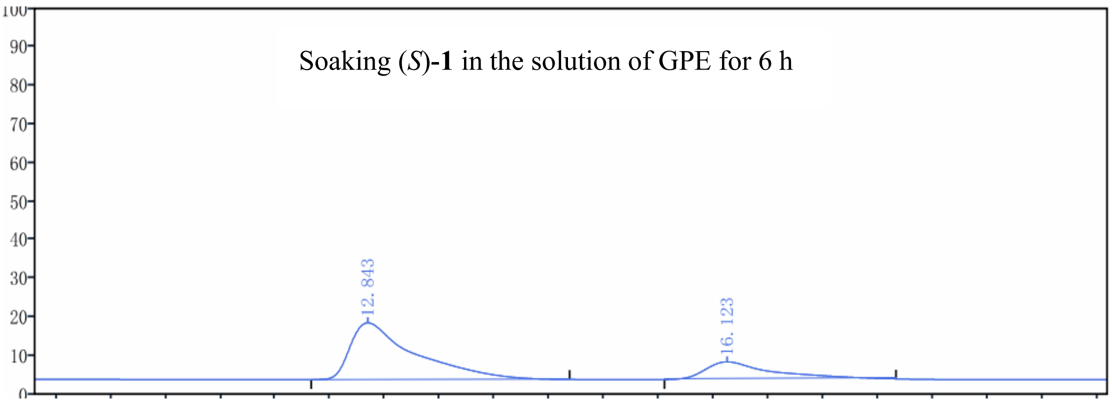

Serial Number	Type	Retention Time [min]	Peak Area	Area %
1	MM	12.843	8806.54	79.05
2	MM	16.123	2334.21	20.95
The Total			11140.75	

The third run of enantiosorption of GPE by (*S*)-1

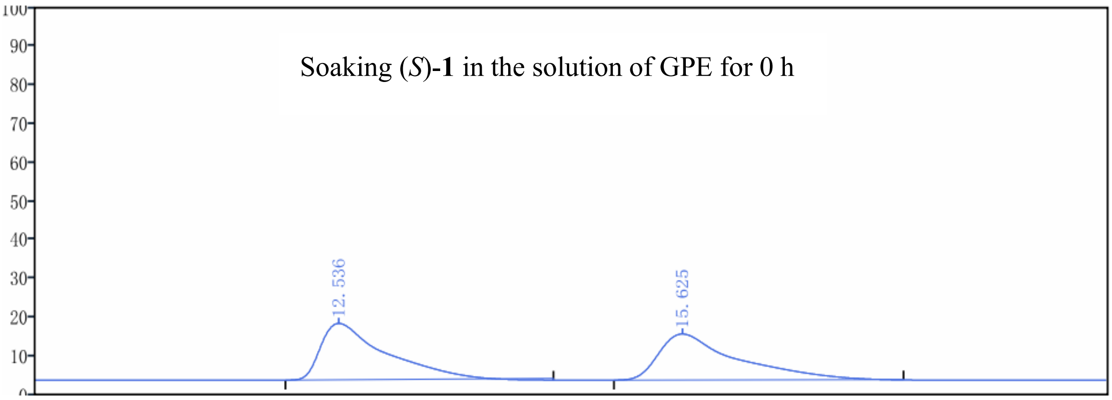

Serial Number	Type	Retention Time [min]	Peak Area	Area %
1	MM	12.536	9868.89	50.02
2	MM	15.625	9862.31	49.98
The Total			19731.20	

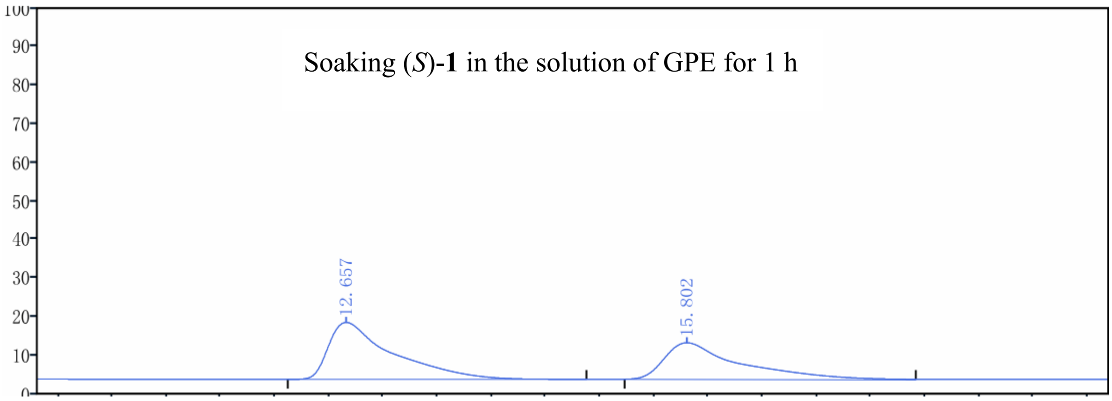

Serial Number	Type	Retention Time [min]	Peak Area	Area %
1	MM	12.657	9091.38	56.63
2	MM	15.802	6962.10	43.37
The Total			16053.48	

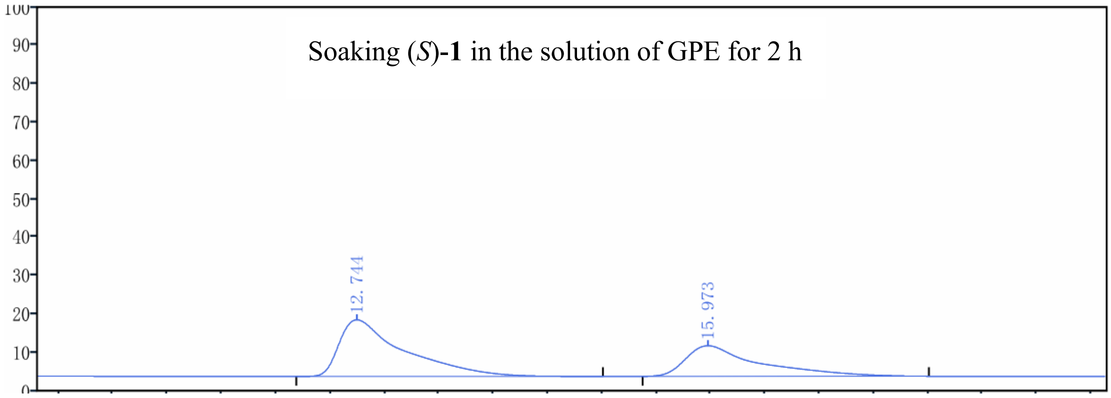

Serial Number	Type	Retention Time [min]	Peak Area	Area %
1	BM	12.744	9075.48	62.30
2	MM	15.973	5491.70	37.70
The Total			14567.18	

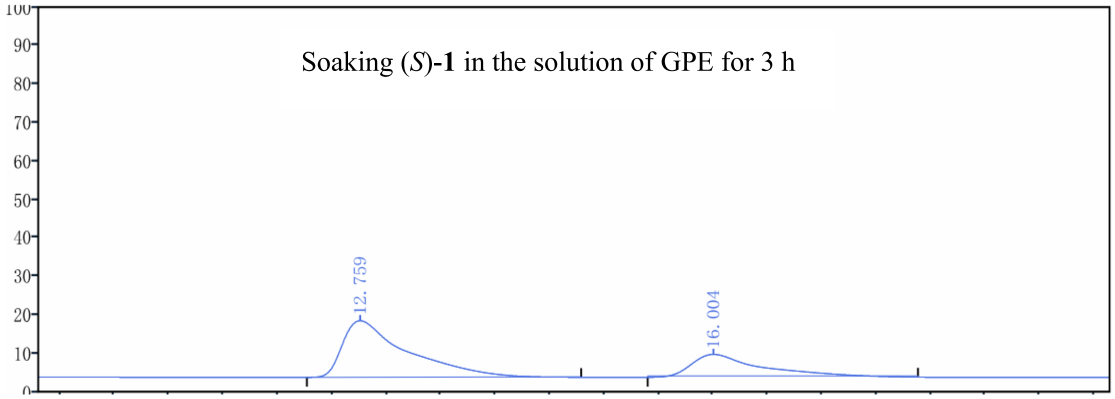

Serial Number	Type	Retention Time [min]	Peak Area	Area %
1	MM	12.759	9070.47	73.01
2	MM	16.004	3353.45	26.99
The Total			12423.92	

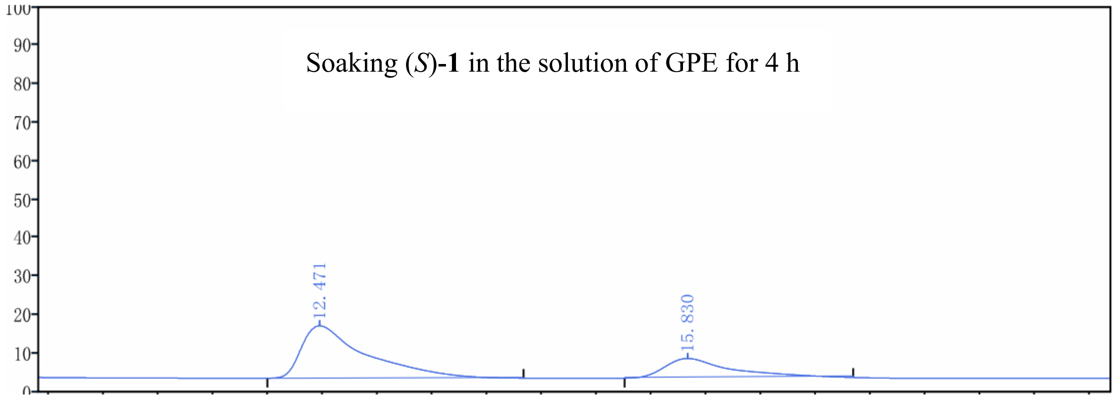

Serial Number	Type	Retention Time [min]	Peak Area	Area %
1	MM	12.471	8962.53	75.09
2	MM	15.830	2973.51	24.91
The Total			11936.04	

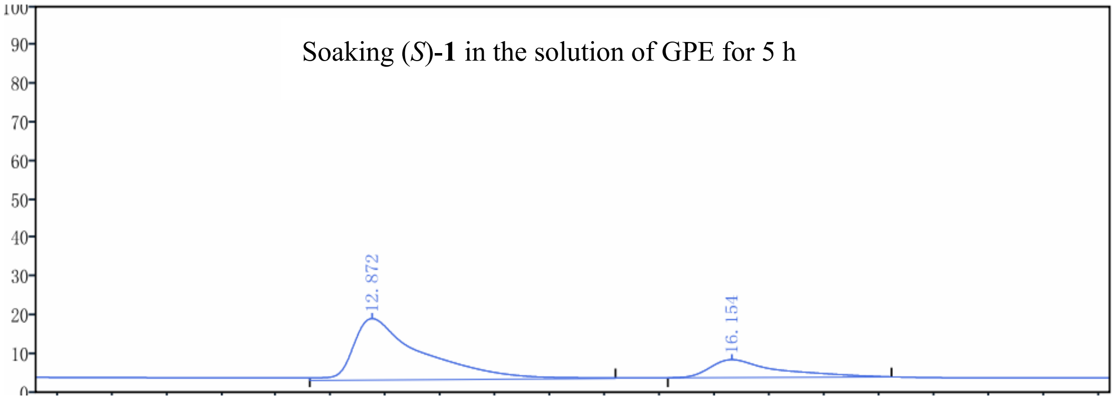

Serial Number	Type	Retention Time [min]	Peak Area	Area %
1	MM	12.872	8949.95	78.32
2	MM	16.154	2477.81	21.68
The Total			11427.76	

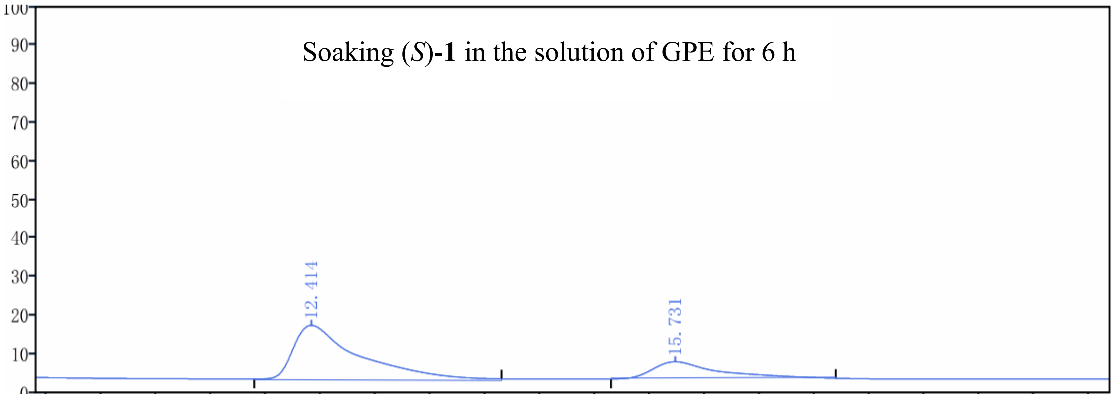

Serial Number	Type	Retention Time [min]	Peak Area	Area %
1	MM	12.414	8945.56	79.13
2	MM	15.731	2359.06	20.87
The Total			11304.62	

13.3 Enantiosorption of GTE by (*S*)-1.

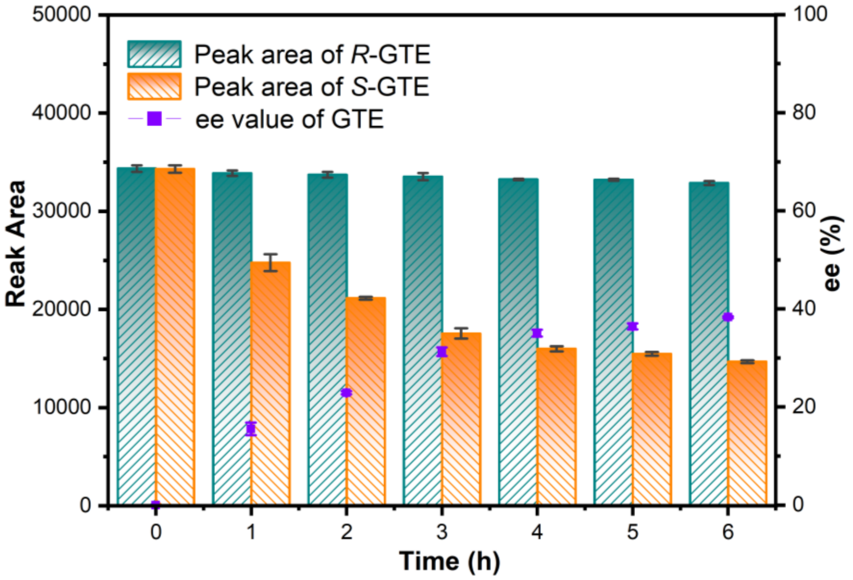

The HPLC spectra are listed as follows.

The first run of enantiosorption of GTE by (*S*)-1

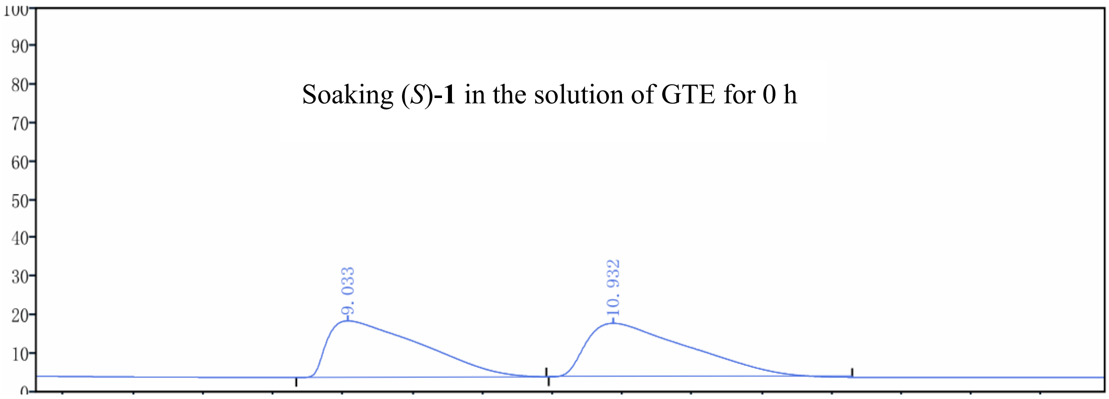

Serial Number	Type	Retention Time [min]	Peak Area	Area %
1	MM	9.033	35313.60	49.90
2	MM	10.932	35450.67	50.10
The Total			70764.27	

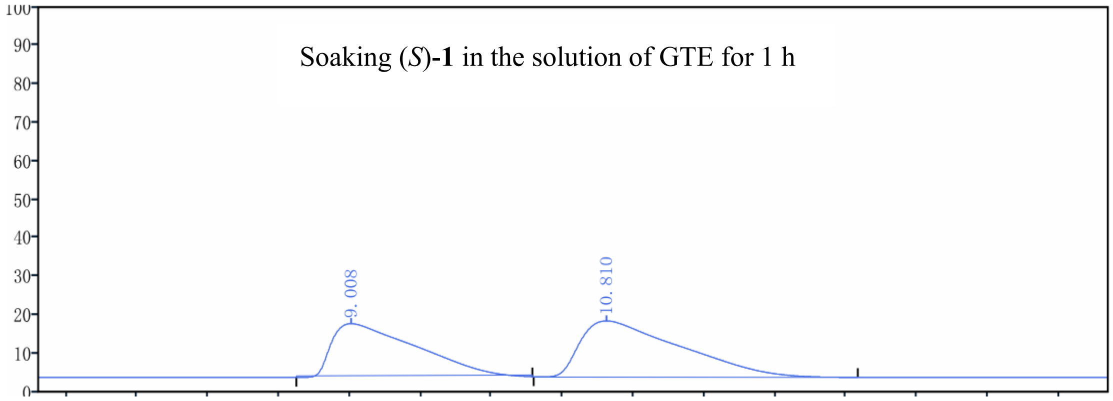

Serial Number	Type	Retention Time [min]	Peak Area	Area %
1	MM	9.008	25762.79	42.97
2	MM	10.810	34192.73	57.03
The Total			59955.52	

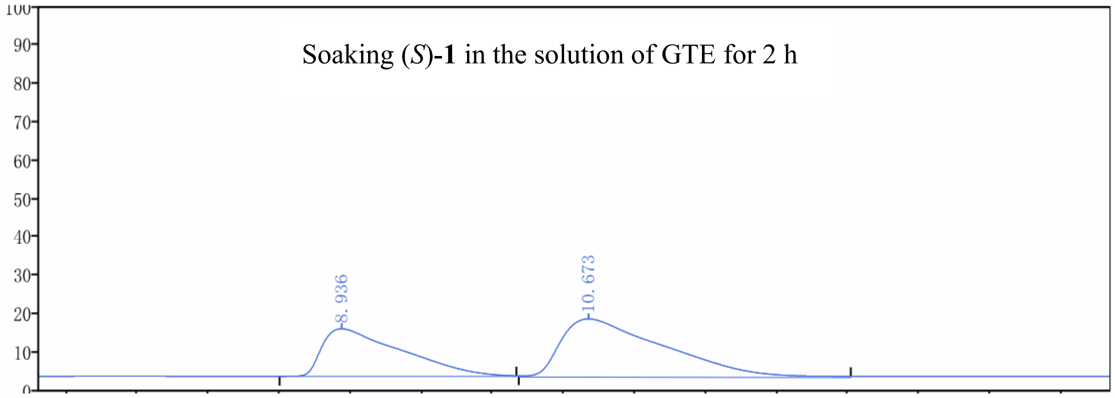

Serial Number	Type	Retention Time [min]	Peak Area	Area %
1	MM	8.936	21266.55	38.45
2	MM	10.673	34048.77	61.55
The Total			55315.32	

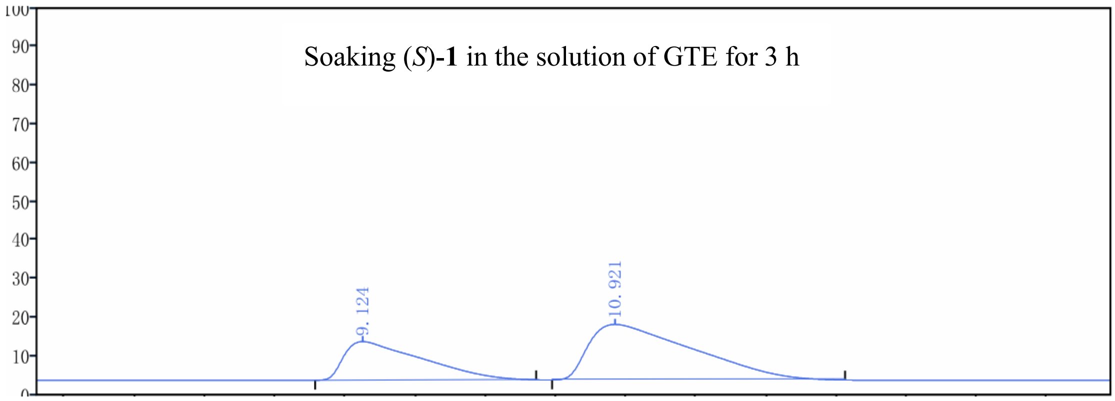

Serial Number	Type	Retention Time [min]	Peak Area	Area %
1	MM	9.124	18150.17	34.85
2	MM	10.921	33931.25	65.15
The Total			52081.42	

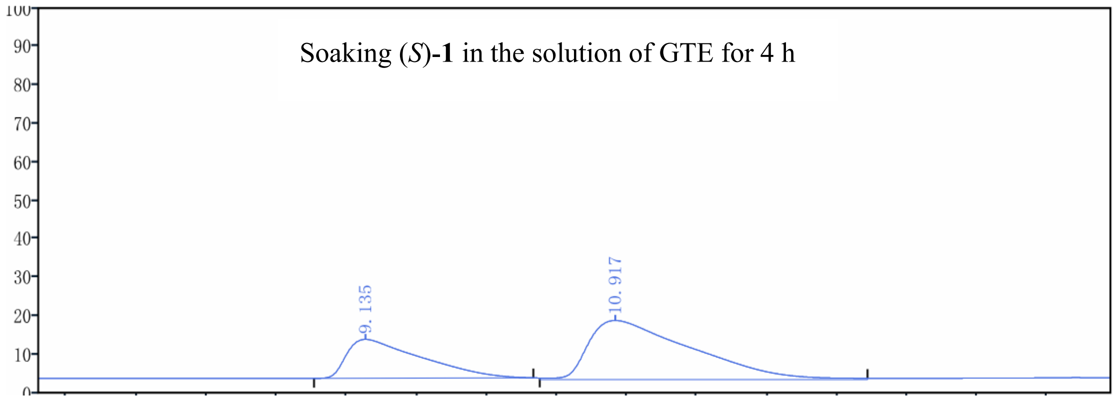

Serial Number	Type	Retention Time [min]	Peak Area	Area %
1	MM	9.135	15773.81	32.22
2	MM	10.917	33184.68	67.78
The Total			48958.49	

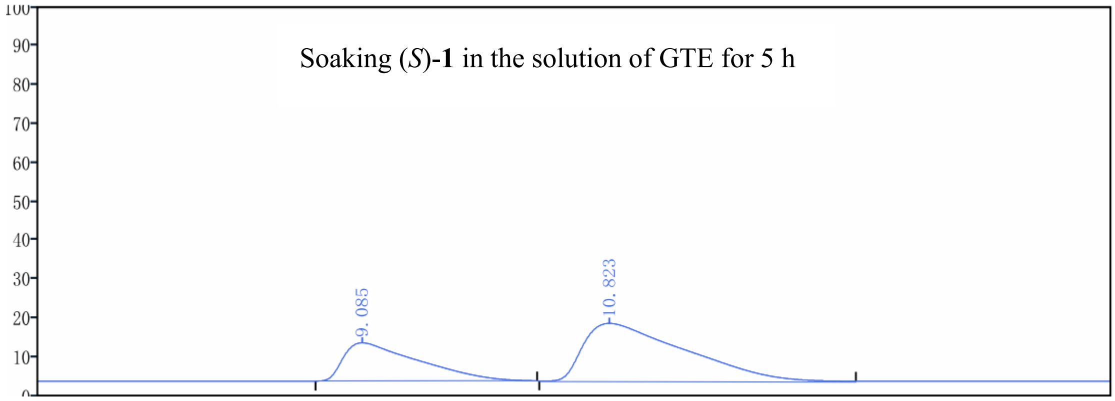

Serial Number	Type	Retention Time [min]	Peak Area	Area %
1	MM	9.085	15506.06	31.89
2	MM	10.823	33114.92	68.11
The Total			48620.98	

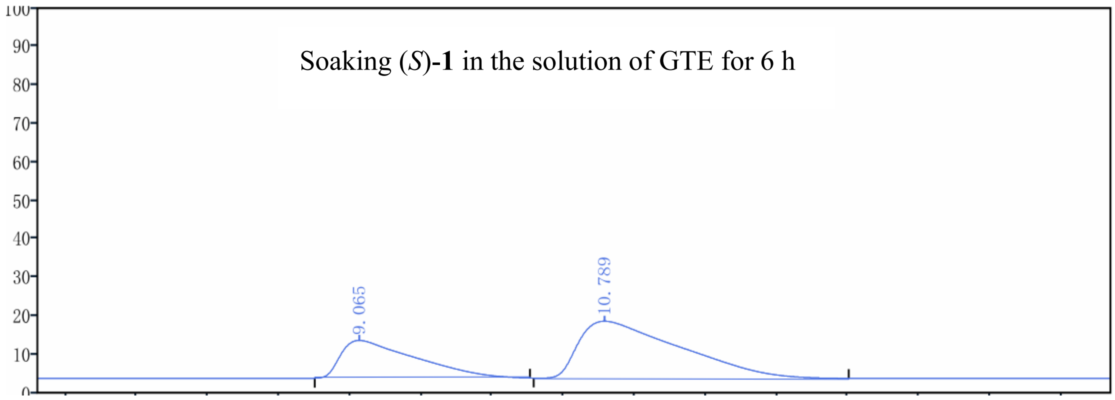

Serial Number	Type	Retention Time [min]	Peak Area	Area %
1	MM	9.065	14755.59	30.85
2	MM	10.789	33074.09	69.15
The Total			47829.68	

The second run of enantiosorption of GTE by (*S*)-1

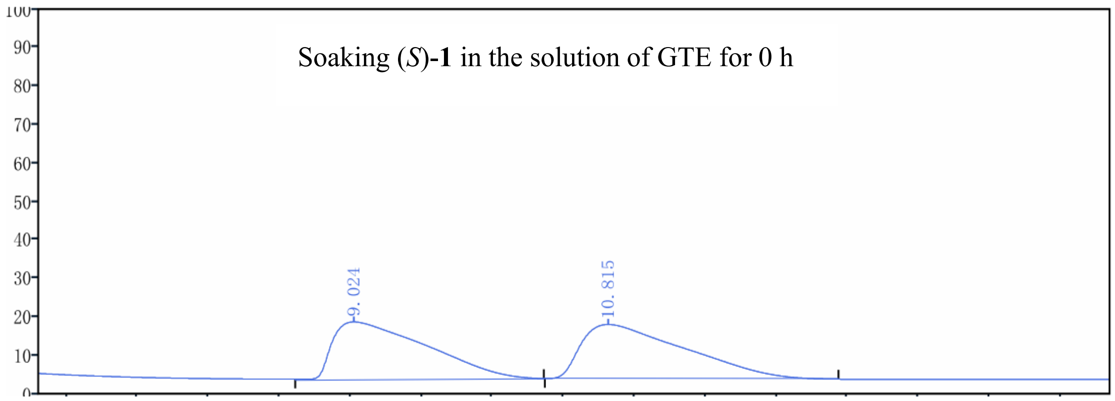

Serial Number	Type	Retention Time [min]	Peak Area	Area %
1	MM	9.024	34546.00	49.99
2	MM	10.815	34565.60	50.01
The Total			69111.60	

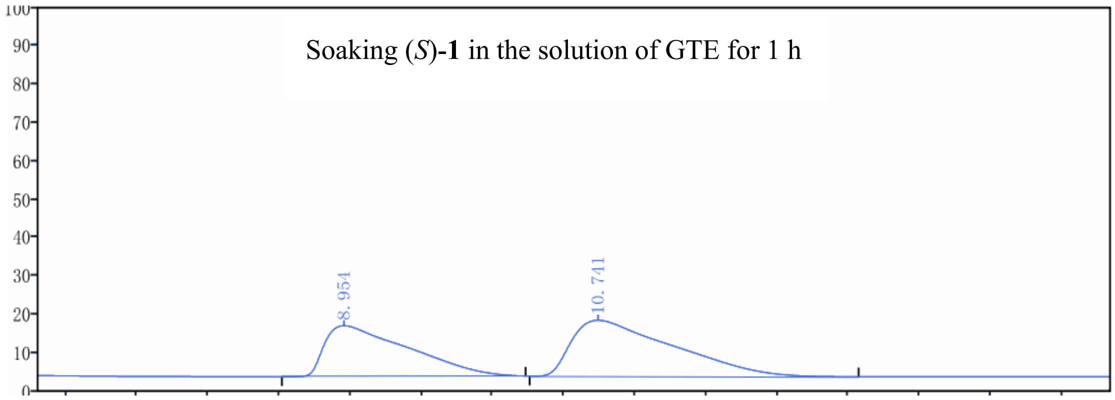

Serial Number	Type	Retention Time [min]	Peak Area	Area %
1	MM	8.954	24386.73	41.96
2	MM	10.741	33726.35	58.04
The Total			58113.08	

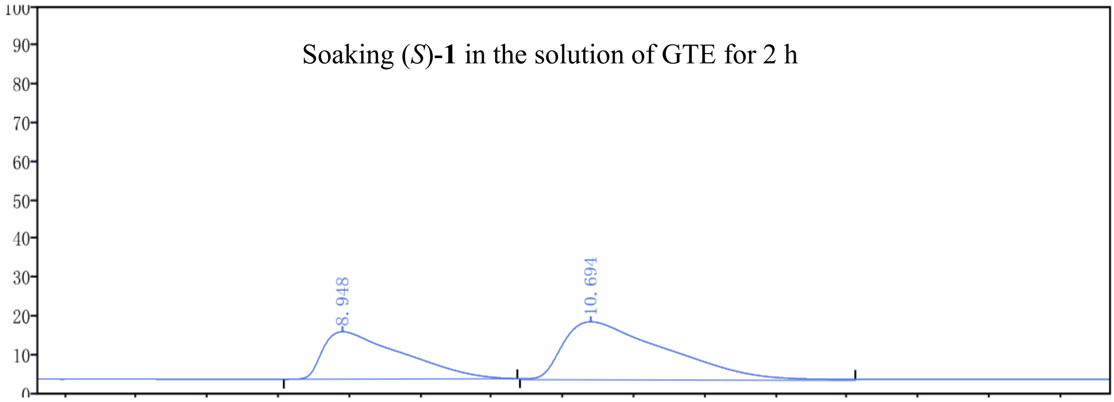

Serial Number	Type	Retention Time [min]	Peak Area	Area %
1	MM	8.948	20988.83	38.47
2	MM	10.694	33575.19	61.53
The Total			54564.02	

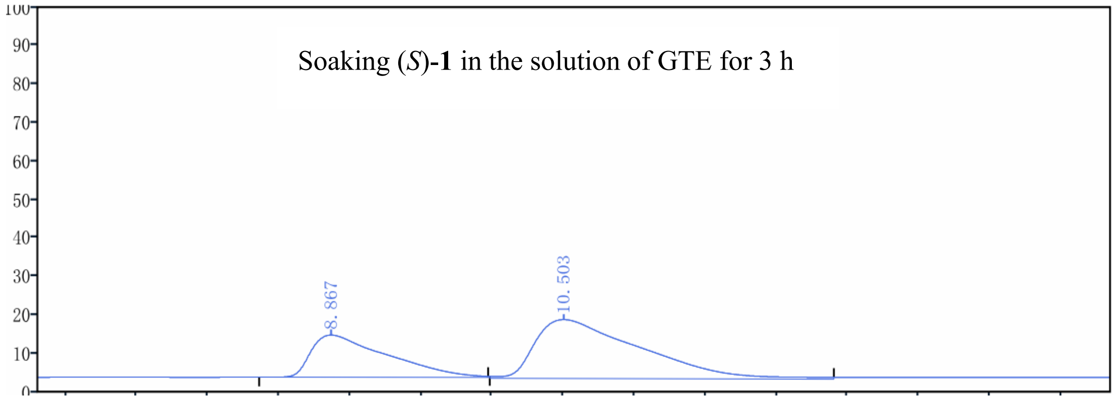

Serial Number	Type	Retention Time [min]	Peak Area	Area %
1	MM	8.867	17356.19	34.19
2	MM	10.503	33407.67	65.81
The Total			50763.86	

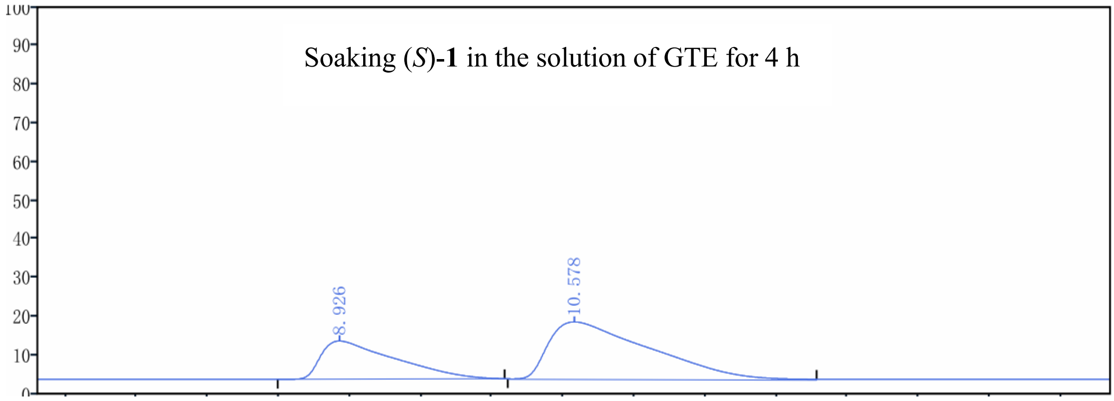

Serial Number	Type	Retention Time [min]	Peak Area	Area %
1	MM	8.926	15923.55	32.33
2	MM	10.578	33331.83	67.67
The Total			49255.38	

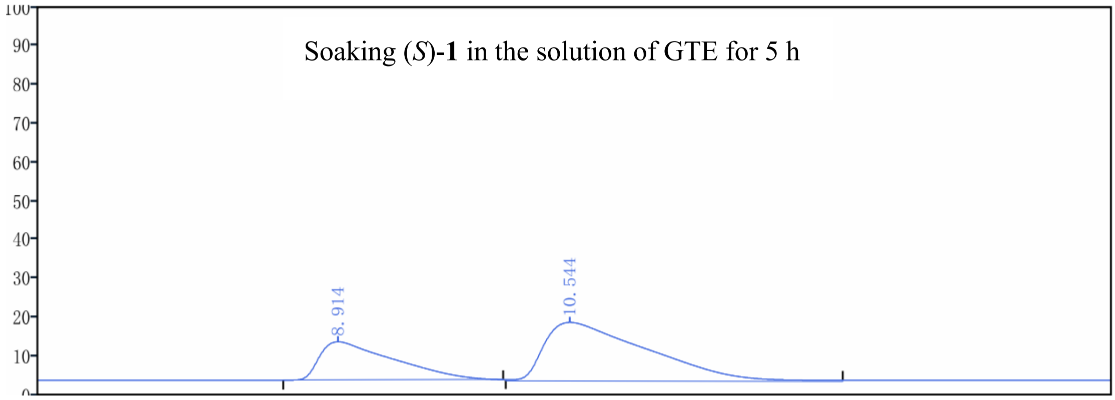

Serial Number	Type	Retention Time [min]	Peak Area	Area %
1	MM	8.914	15275.00	31.45
2	MM	10.544	33300.22	68.55
The Total			48575.22	

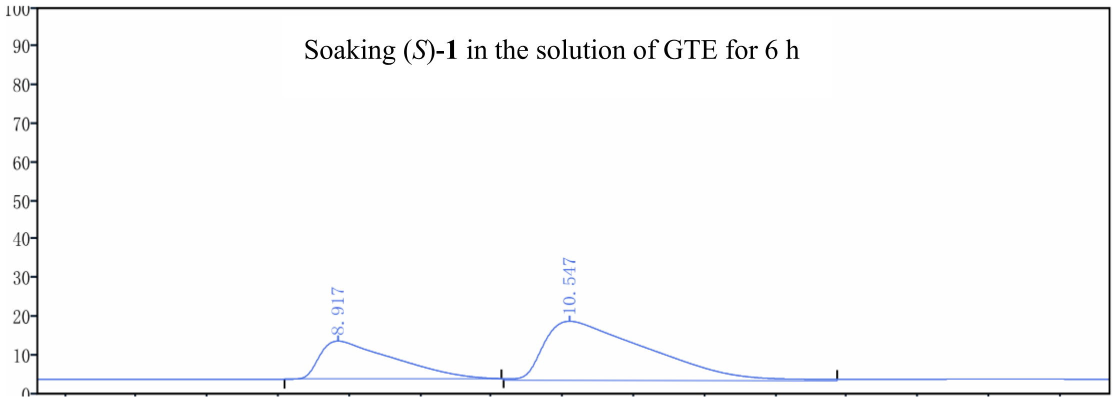

Serial Number	Type	Retention Time [min]	Peak Area	Area %
1	MM	8.917	14766.34	30.96
2	MM	10.547	32929.47	69.04
The Total			47695.81	

The third run of enantiosorption of GTE by (*S*)-1

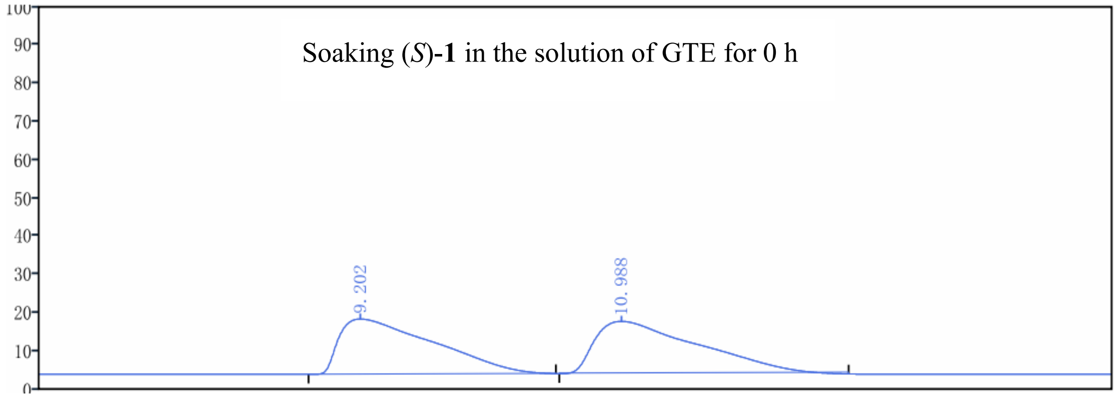

Serial Number	Type	Retention Time [min]	Peak Area	Area %
1	MM	9.202	34516.98	49.99
2	MM	10.988	34530.35	50.01
The Total			69047.33	

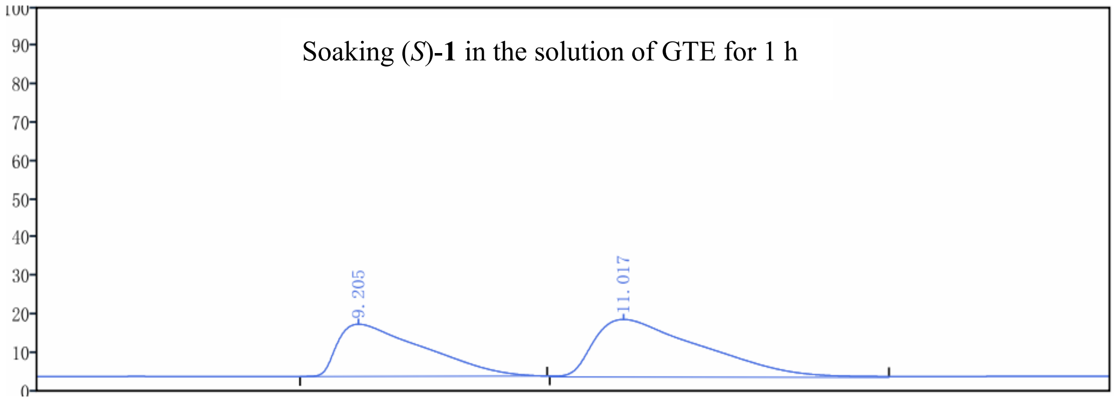

Serial Number	Type	Retention Time [min]	Peak Area	Area %
1	MM	9.205	24170.63	41.77
2	MM	11.017	33695.84	58.23
The Total			57866.47	

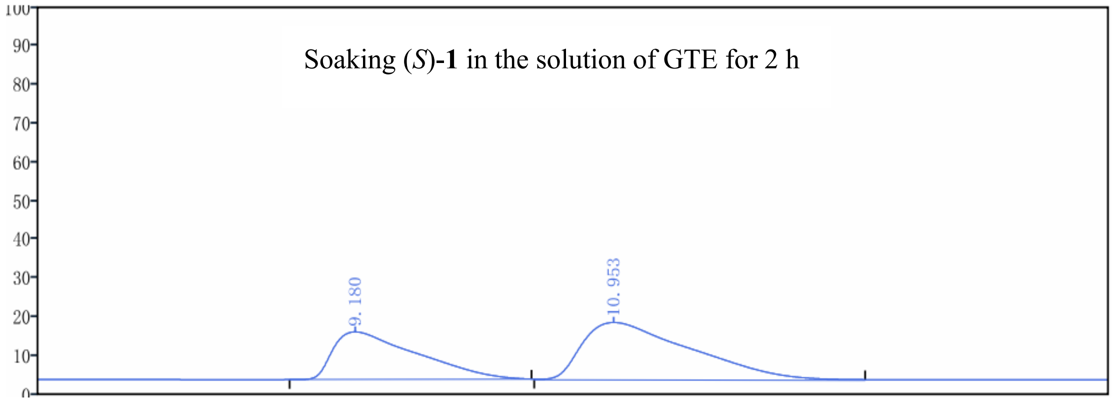

Serial Number	Type	Retention Time [min]	Peak Area	Area %
1	MM	9.180	21191.31	38.73
2	MM	10.953	33527.96	61.27
The Total			54719.27	

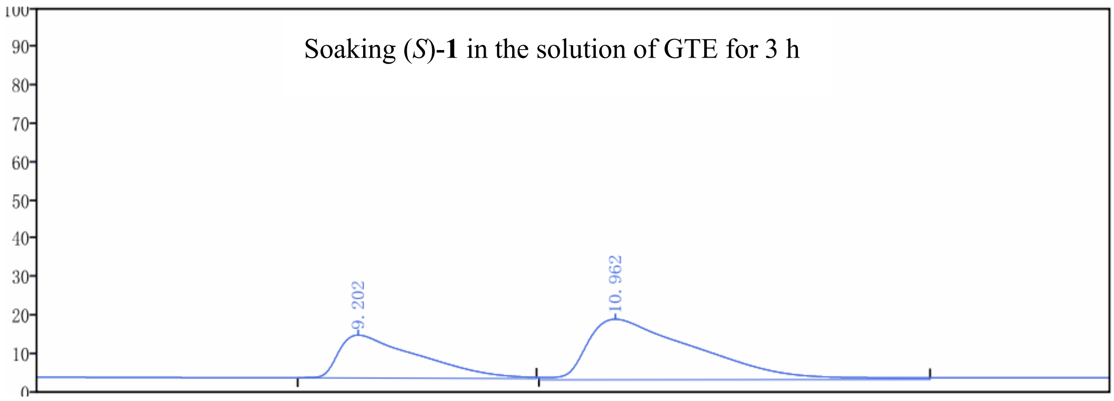

Serial Number	Type	Retention Time [min]	Peak Area	Area %
1	MM	9.202	17156.23	34.03
2	MM	10.962	33257.76	65.97
The Total			50413.99	

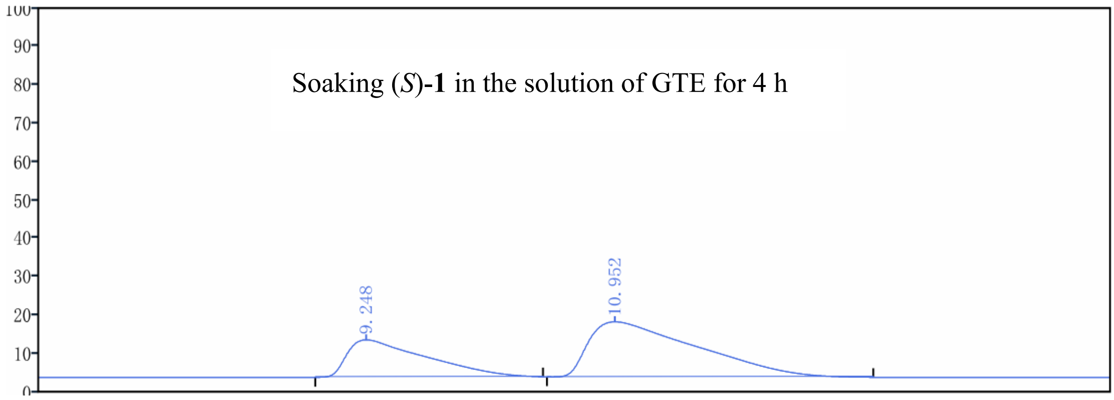

Serial Number	Type	Retention Time [min]	Peak Area	Area %
1	MM	9.248	16280.09	32.88
2	MM	10.952	33229.97	67.12
The Total			49510.06	

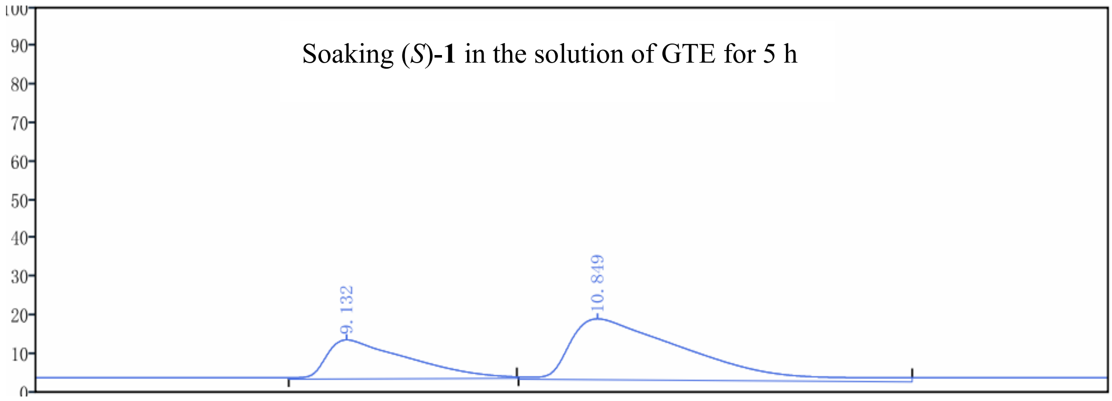

Serial Number	Type	Retention Time [min]	Peak Area	Area %
1	MM	9.132	14720.10	31.04
2	MM	10.849	32707.52	68.96
The Total			47427.62	

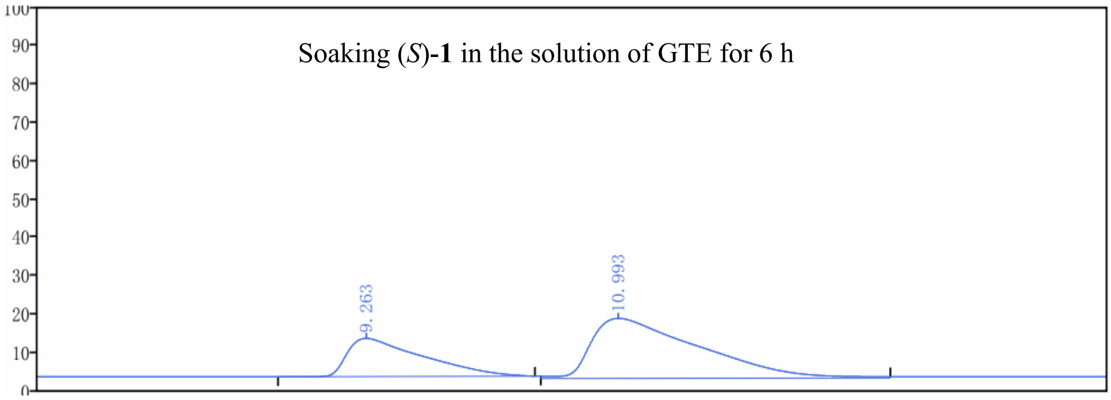

Serial Number	Type	Retention Time [min]	Peak Area	Area %
1	BM	9.263	14511.04	30.75
2	MM	10.993	32674.68	69.25
The Total			47185.72	

14. Figure S11. Binding energy difference for (R)- and (S)- enantiomers of epoxides resolved by (*S*)-1[3].

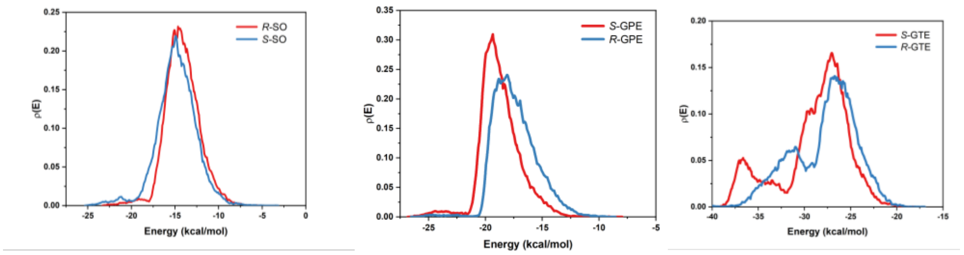

15. Figure S12. HPLC spectra of epoxides (in Figure 3).

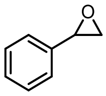

Chiral HPLC analysis: Daicel Chiralce IC: hexane/*i*-PrOH = 95/5, flow rate = 1 mL/min, 220 nm, t_R = 5.164 min, t_R = 5.583 min.

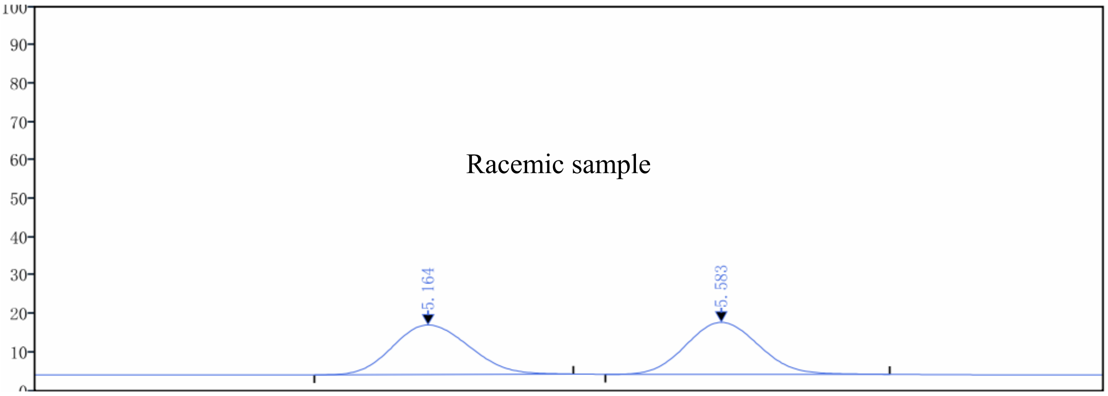

Serial Number	Type	Retention Time [min]	Peak Area	Area %
1	MM	5.164	1833.13	49.99
2	MM	5.583	1833.65	50.01
The Total			3666.78	

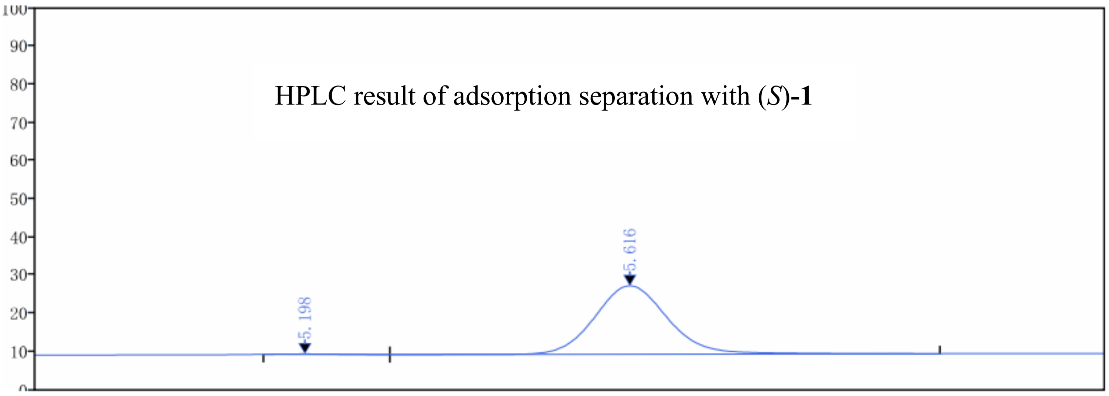

Serial Number	Type	Retention Time [min]	Peak Area	Area %
1	MM	5.198	6.19	0.48
2	MM	5.616	1290.83	99.52
The Total			1297.02	

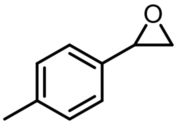

Chiral HPLC analysis: Daicel Chiralce OD: hexane/*i*-PrOH = 98/2, flow rate =0.8 mL/min, 220 nm, t_R = 6.411 min, t_R = 8.039 min.

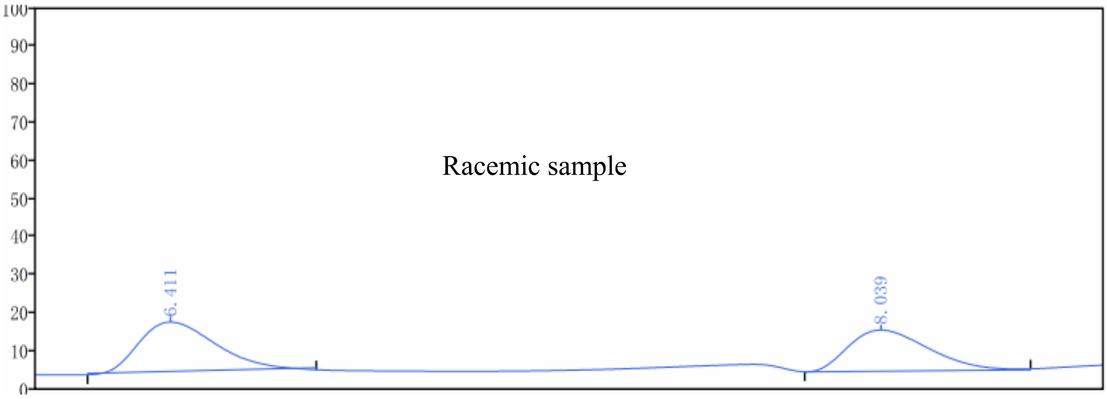

Serial Number	Type	Retention Time [min]	Peak Area	Area %
1	MM	6.411	10198.83	52.58
2	BM	8.039	9196.26	47.42
The Total			19395.09	

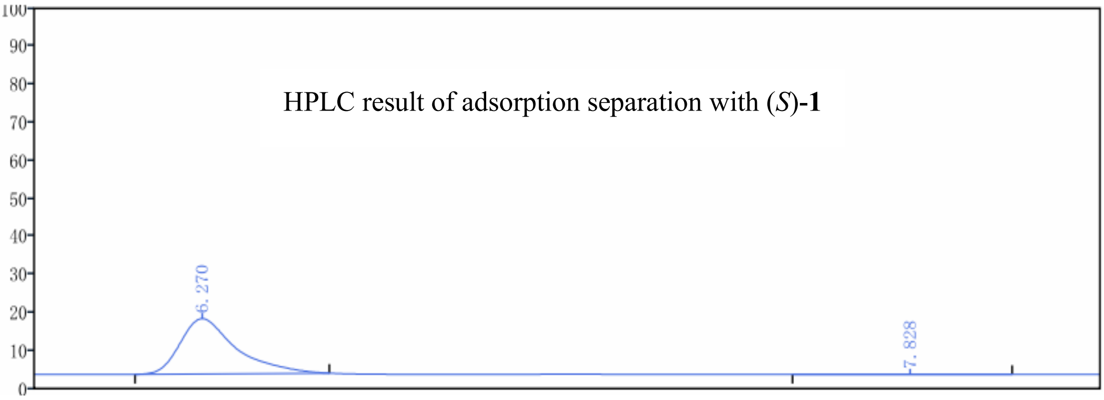

Serial Number	Type	Retention Time [min]	Peak Area	Area %
1	MM	6.270	6440.19	99.99
2	MM	7.828	0.24	0.01
The Total			6440.43	

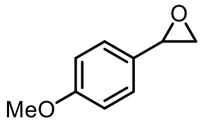

Chiral HPLC analysis: Daicel Chiralce OD: hexane/*i*-PrOH = 98/2, flow rate = 0.8 mL/min, 220 nm, t_R = 6.125 min, t_R = 7.048 min.

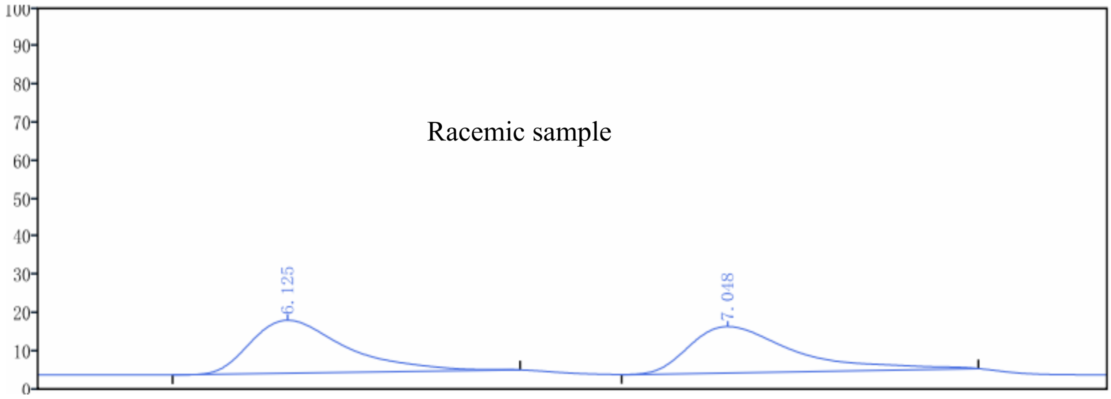

Serial Number	Type	Retention Time [min]	Peak Area	Area %
1	MM	6.125	11681.13	50.87
2	VM	7.048	11280.00	49.13
The Total			22961.13	

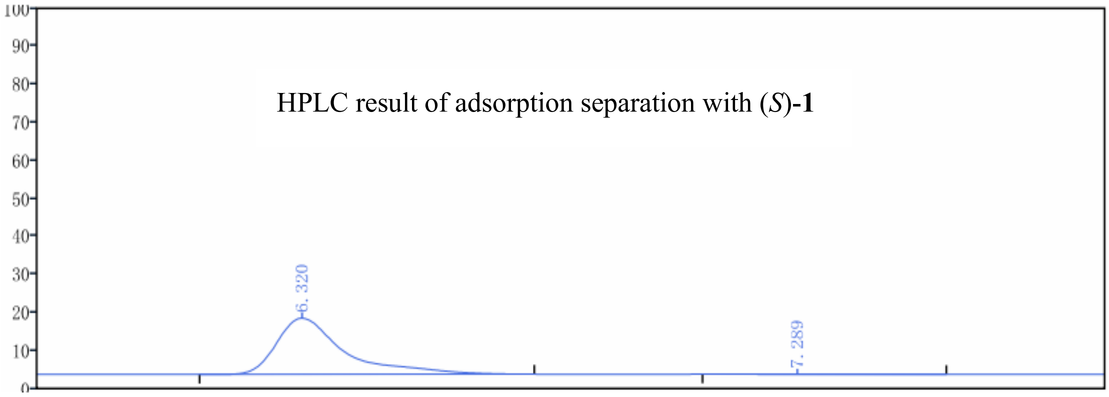

Serial Number	Type	Retention Time [min]	Peak Area	Area %
1	MM	6.320	6440.19	99.99
2	MM	7.289	0.24	0.01
The Total			6440.43	

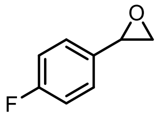

Chiral HPLC analysis: Daicel Chiralce OD: hexane/*i*-PrOH = 99/1, flow rate = 0.7 mL/min, 220 nm, t_R = 5.405 min, t_R = 5.883 min.

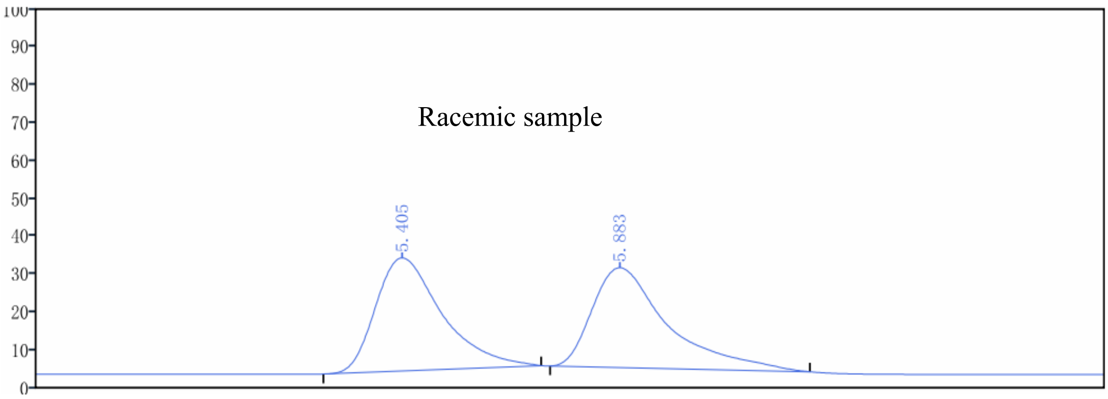

Serial Number	Type	Retention Time [min]	Peak Area	Area %
1	MM	5.405	7861.67	49.89
2	BM	5.883	7897.24	50.11
The Total			15758.91	

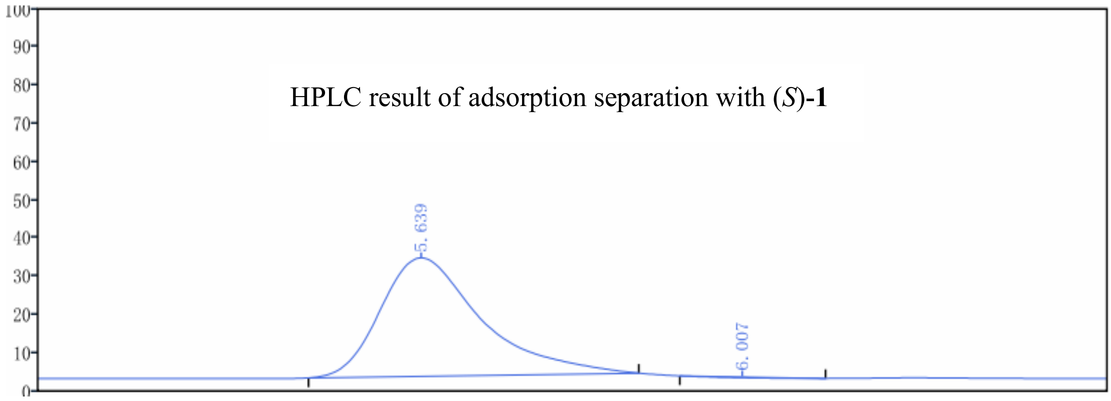

Serial Number	Type	Retention Time [min]	Peak Area	Area %
1	MM	5.639	6971.57	99.44
2	MM	6.007	39.02	0.56
The Total			7010.59	

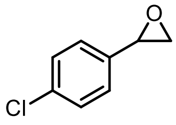

Chiral HPLC analysis: Daicel Chiralce OD: hexane/*i*-PrOH = 99/1, flow rate = 0.7 mL/min, 220 nm, t_R = 6.302 min, t_R = 6.974 min.

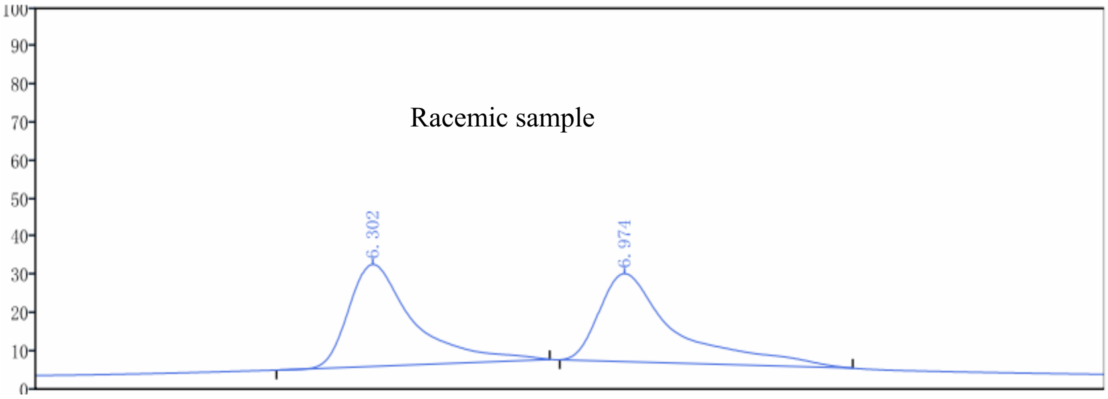

Serial Number	Type	Retention Time [min]	Peak Area	Area %
1	MM	6.302	8857.30	49.97
2	BM	6.974	8869.30	50.03
The Total			17726.60	

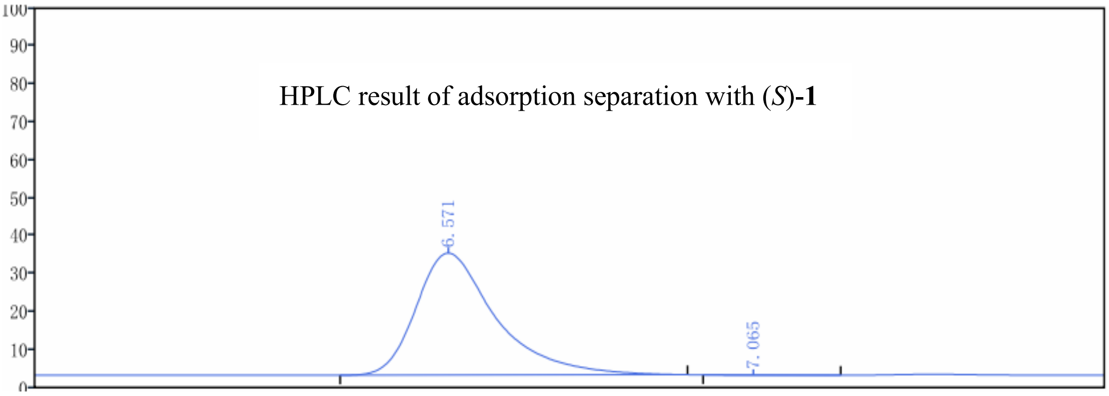

Serial Number	Type	Retention Time [min]	Peak Area	Area %
1	MM	6.571	3591.15	99.90
2	MM	7.065	3.42	0.10
The Total			3594.57	

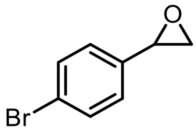

Chiral HPLC analysis: Daicel Chiralce OD: hexane/*i*-PrOH = 95/5, flow rate = 1 mL/min, 220 nm, t_R = 13.313 min, t_R = 17.385 min.

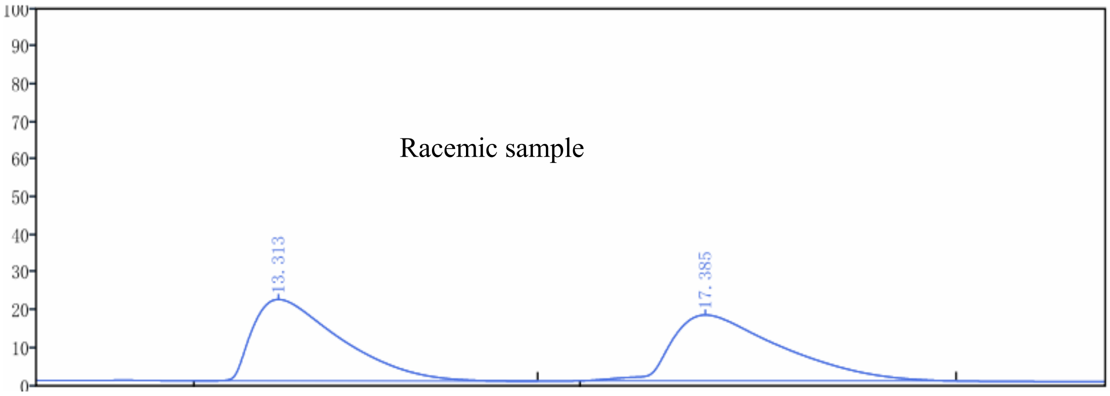

Serial Number	Type	Retention Time [min]	Peak Area	Area %
1	BB	13.313	16824.71	49.77
2	MM	17.385	16979.89	50.23
The Total			33804.60	

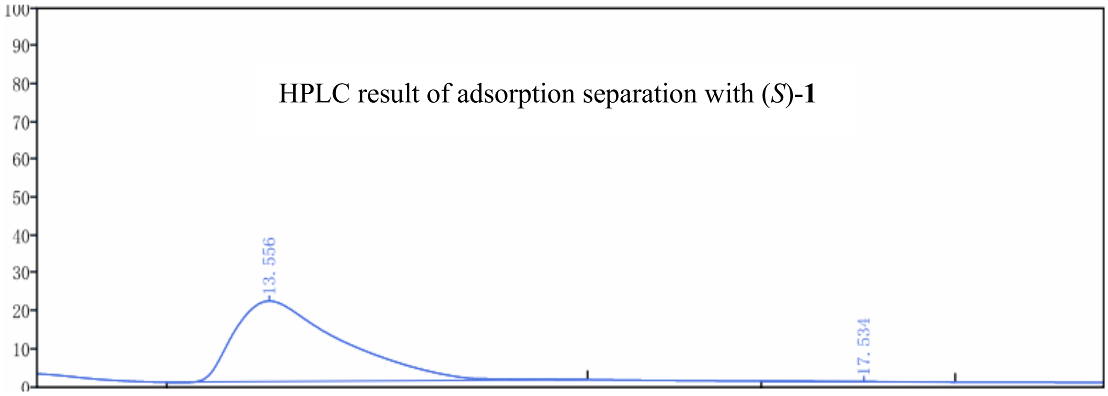

Serial Number	Type	Retention Time [min]	Peak Area	Area %
1	MM	13.556	3703.66	99.92
2	MM	17.534	2.94	0.08
The Total			3706.60	

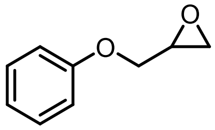

Chiral HPLC analysis: Daicel Chiralce AS-H: hexane/*i*-PrOH =98/2; flow rate =0.8 mL/min; 254 nm; t_R = 12.376 min, t_R = 15.708 min.

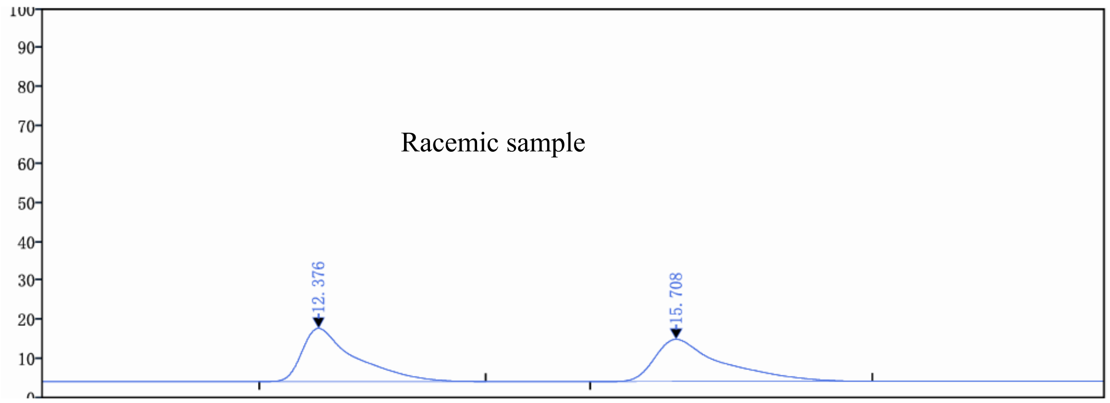

Serial Number	Type	Retention Time [min]	Peak Area	Area %
1	BM	12.376	411.07	49.67
2	MM	15.708	416.55	50.33
The Total			827.61	

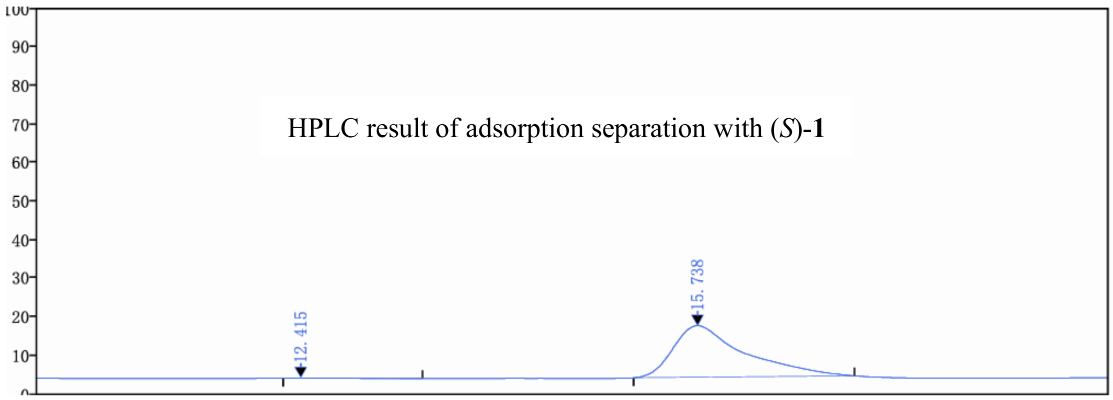

Serial Number	Type	Retention Time [min]	Peak Area	Area %
1	MM	12.415	0.32	0.01
2	MM	15.738	5411.83	99.99
The Total			5412.16	

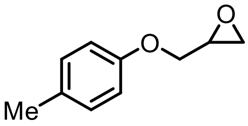

Chiral HPLC analysis: Daicel Chiralce OD: hexane/*i*-PrOH =98/2; flow rate = 1 mL/min; 210 nm; t_R = 8.725 min, t_R =10.895 min.

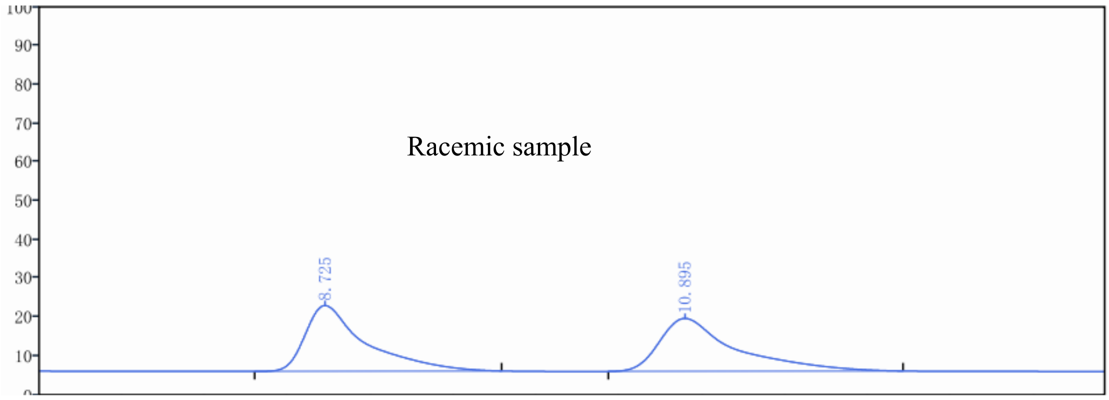

Serial Number	Type	Retention Time [min]	Peak Area	Area %
1	MM	8.725	1467.44	49.84
2	MM	10.895	1476.88	50.16
The Total			2944.32	

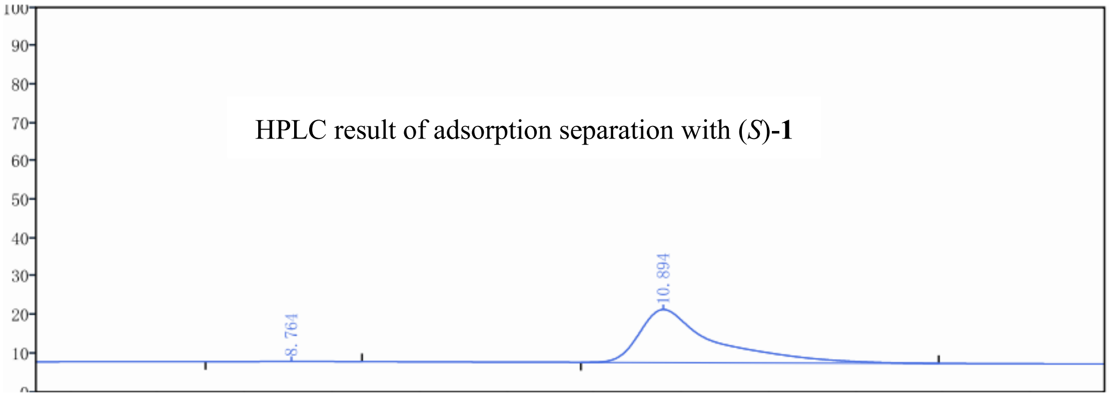

Serial Number	Type	Retention Time [min]	Peak Area	Area %
1	MM	8.764	3.90	0.42
2	BM	10.894	934.87	99.58
The Total			938.77	

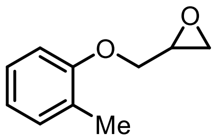

Chiral HPLC analysis: Daicel Chiralce IA: hexane/*i*-PrOH =99/1; flow rate = 1 mL/min; 220 nm; t_R = 6.002 min, t_R =6.350 min.

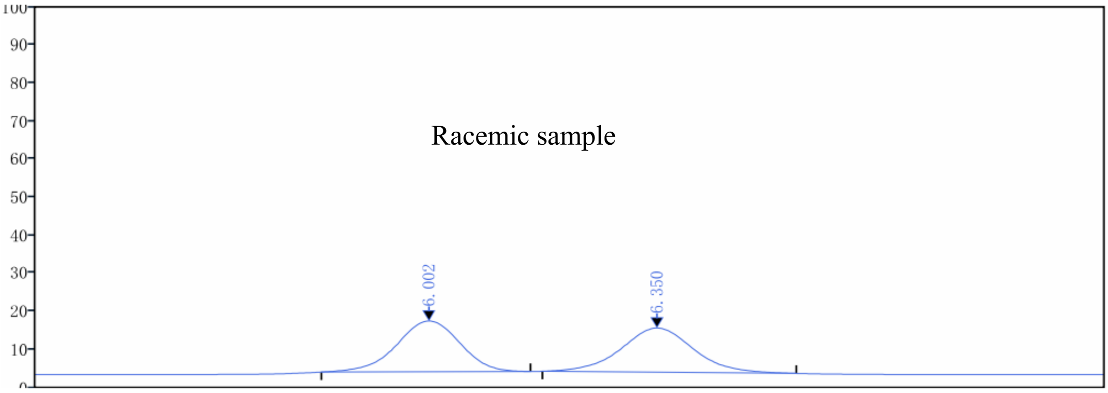

Serial Number	Type	Retention Time [min]	Peak Area	Area %
1	MM	6.002	7975.05	49.99
2	MM	6.350	7977.67	50.01
The Total			15952.72	

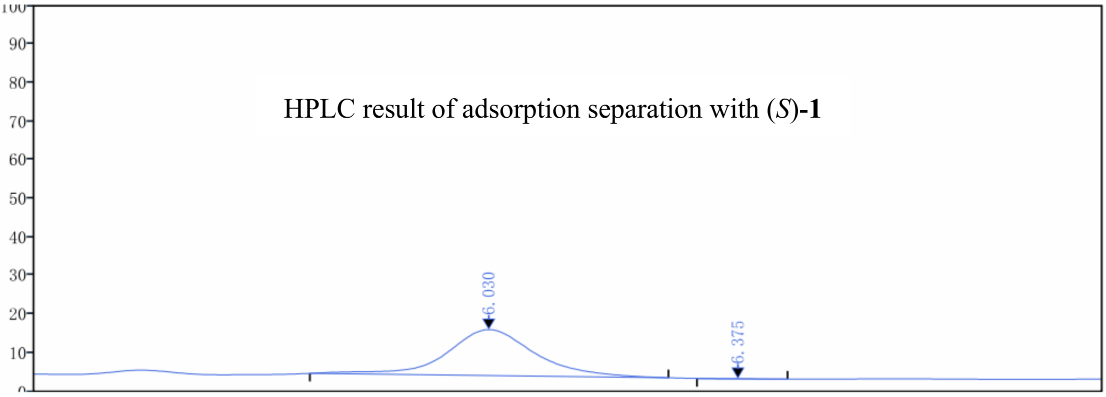

Serial Number	Type	Retention Time [min]	Peak Area	Area %
1	MM	6.030	4761.71	99.77
2	MM	6.375	10.93	0.23
The Total			4772.64	

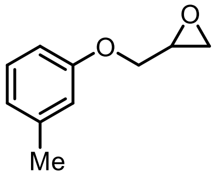

Chiral HPLC analysis: Daicel Chiralce AS-H: hexane/*i*-PrOH =98/2; flow rate = 1 mL/min; 220 nm; t_R = 7.905 min, t_R =8.791 min.

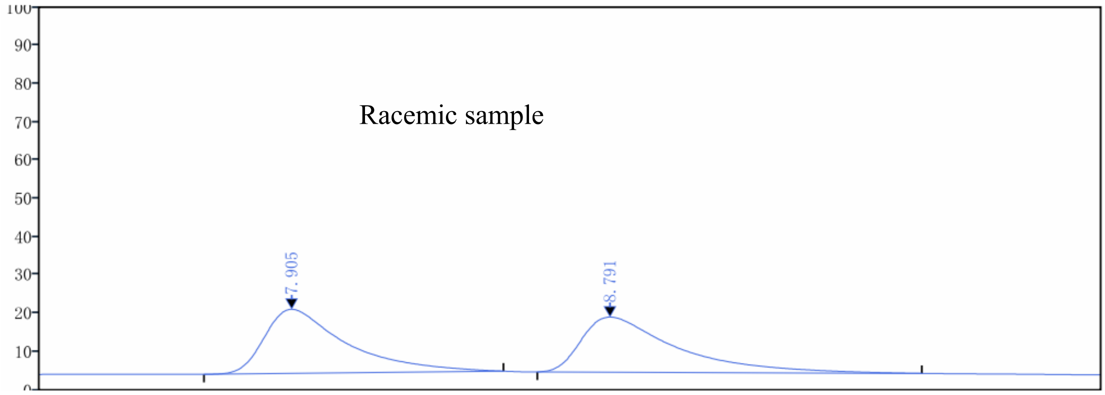

Serial Number	Type	Retention Time [min]	Peak Area	Area %
1	MM	7.905	6299.49	49.99
2	MM	8.791	6300.87	50.01
The Total			12600.35	

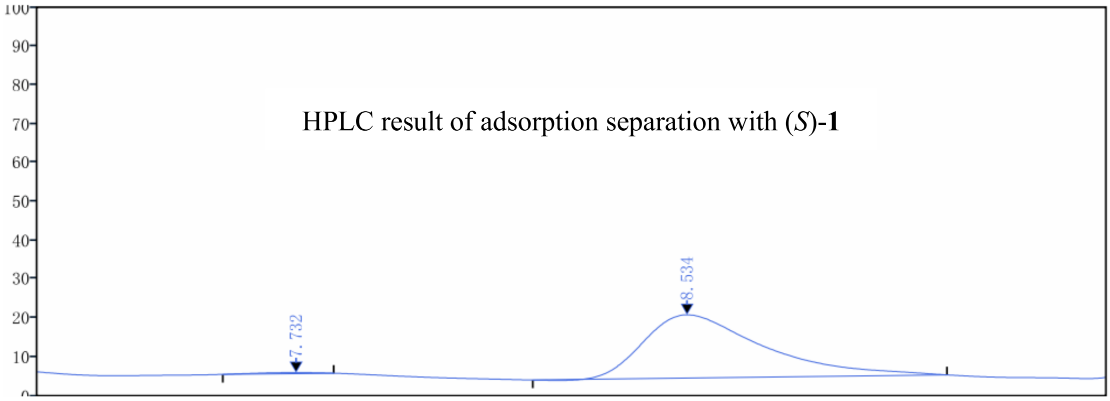

Serial Number	Type	Retention Time [min]	Peak Area	Area %
1	MM	7.732	7.51	0.88
2	MM	8.534	847.22	99.12
The Total			854.72	

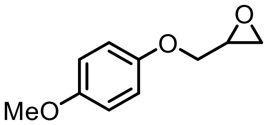
Chiral HPLC analysis: Daicel Chiralce OD: hexane/*i*-PrOH =90/10; flow rate = 1 mL/min; 254 nm; t_R = 8.478 min, t_R =11.095 min.

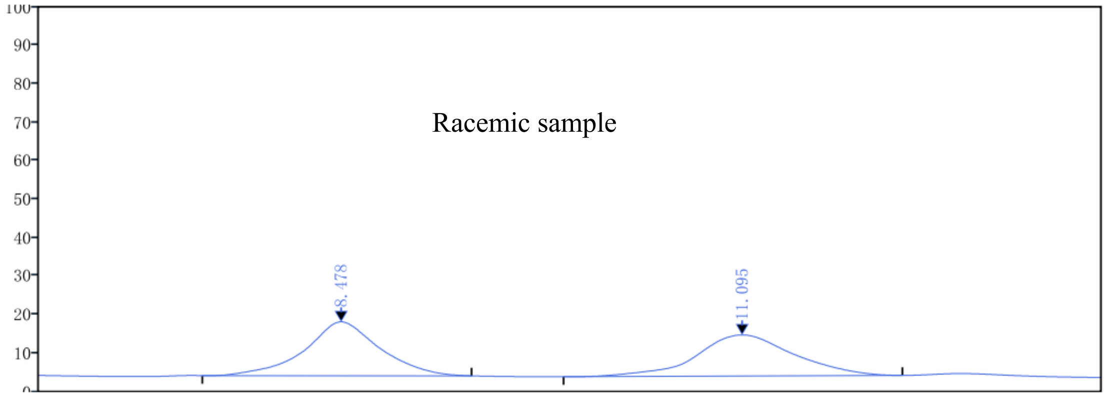

Serial Number	Type	Retention Time [min]	Peak Area	Area %
1	MM	8.478	515.46	50.07
2	MM	11.095	514.05	49.93
The Total			1029.51	

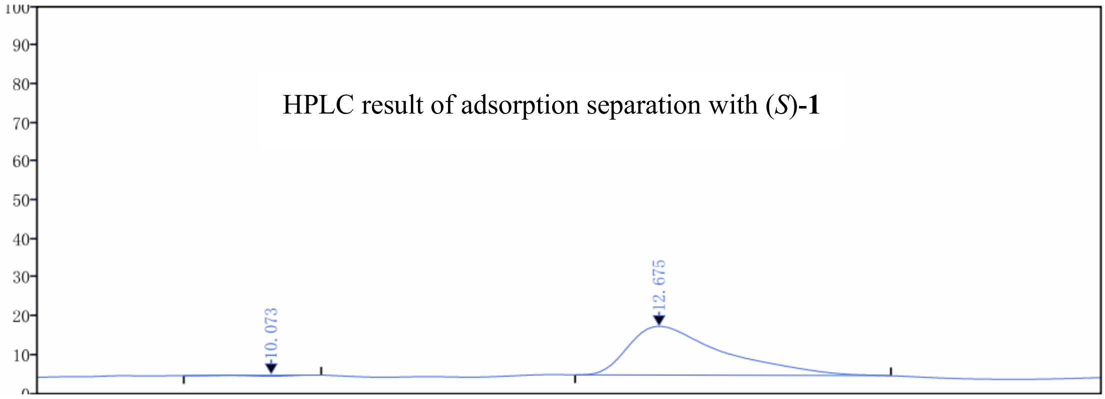

Serial Number	Type	Retention Time [min]	Peak Area	Area %
1	MM	10.073	3.49	0.52
2	BM	12.675	667.48	99.48
The Total			670.97	

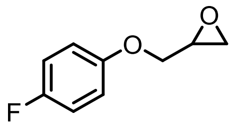

Chiral HPLC analysis: Daicel Chiralce OD: hexane/*i*-PrOH =98/2; flow rate = 1 mL/min; 210 nm; t_R = 8.042 min, t_R =9.084 min.

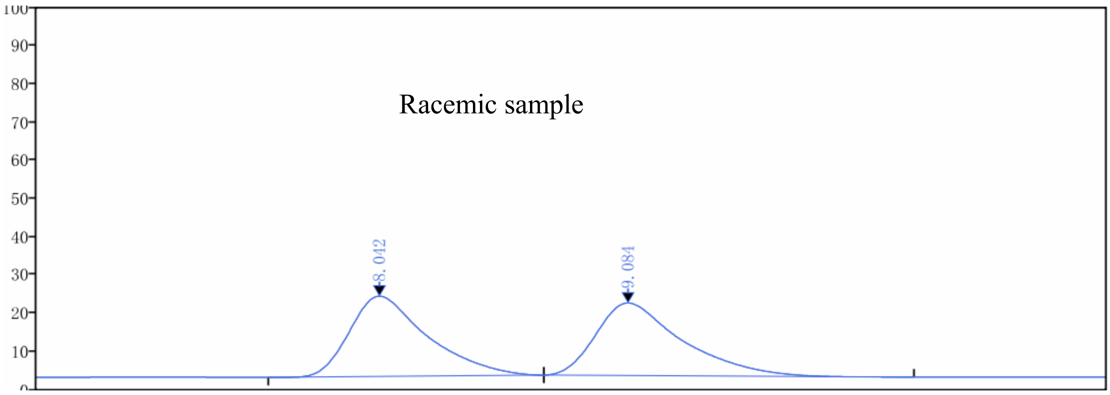

Serial Number	Type	Retention Time [min]	Peak Area	Area %
1	MM	8.042	14870.59	49.98
2	MM	9.084	14883.19	50.02
The Total			29753.78	

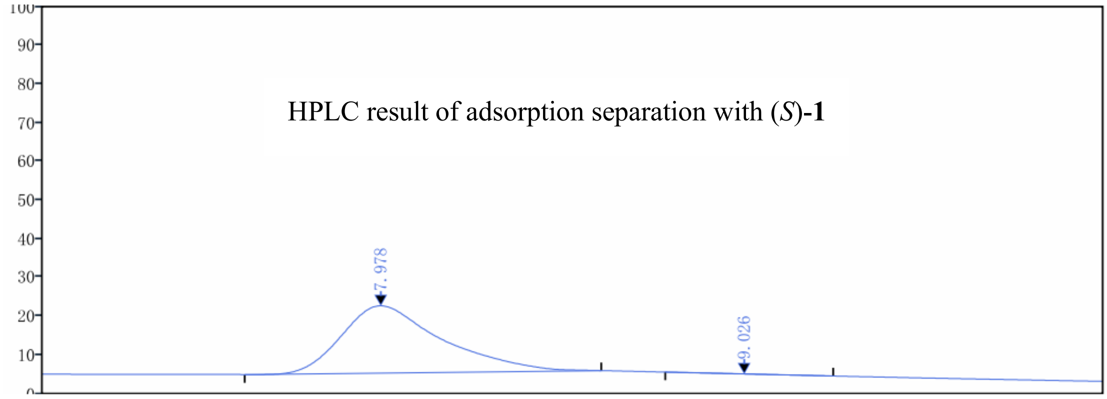

Serial Number	Type	Retention Time [min]	Peak Area	Area %
1	MM	7.978	465.76	99.95
2	MM	9.026	0.26	0.05
The Total			466.02	

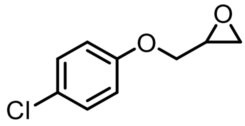

Chiral HPLC analysis: Daicel Chiralce OJ-H: hexane/*i*-PrOH =98/2; flow rate = 1 mL/min; 210 nm; t_R = 15.477 min, t_R =20.205 min.

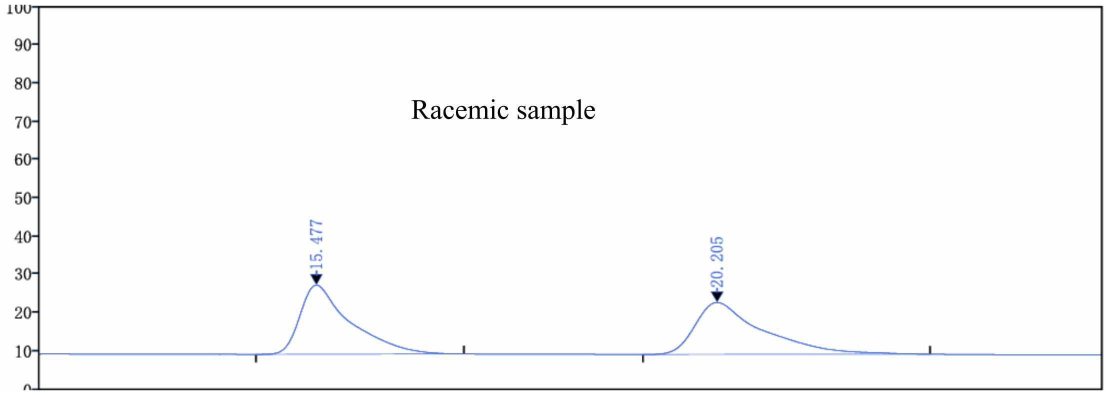

Serial Number	Type	Retention Time [min]	Peak Area	Area %
1	BM	15.477	1371.20	49.71
2	BM	20.205	1387.47	50.29
The Total			2758.67	

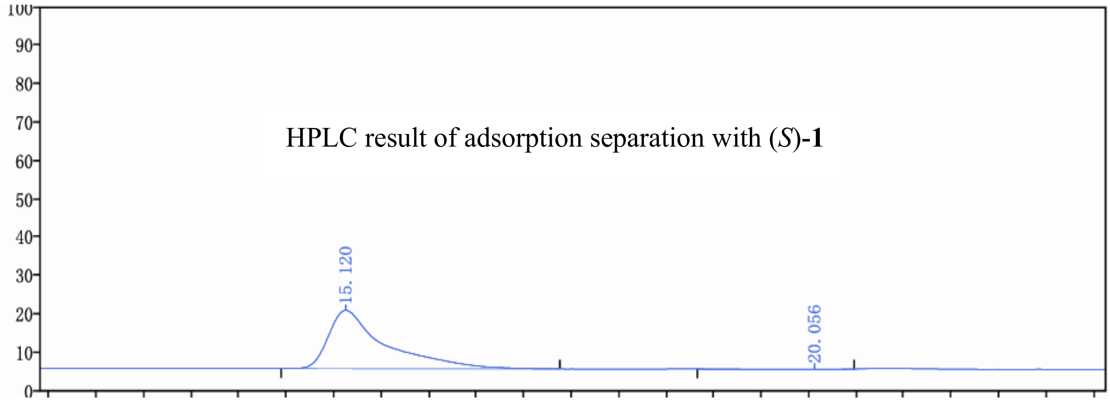

Serial Number	Type	Retention Time [min]	Peak Area	Area %
1	BM	15.120	711.01	99.53
2	MM	20.056	3.37	0.47
The Total			21965.58	

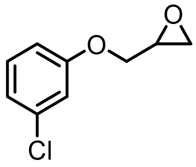

Chiral HPLC analysis: Daicel Chiralce AD-H: hexane/*i*-PrOH =98/2; flow rate = 1 mL/min; 254 nm; t_R = 9.625 min, t_R =11.395 min.

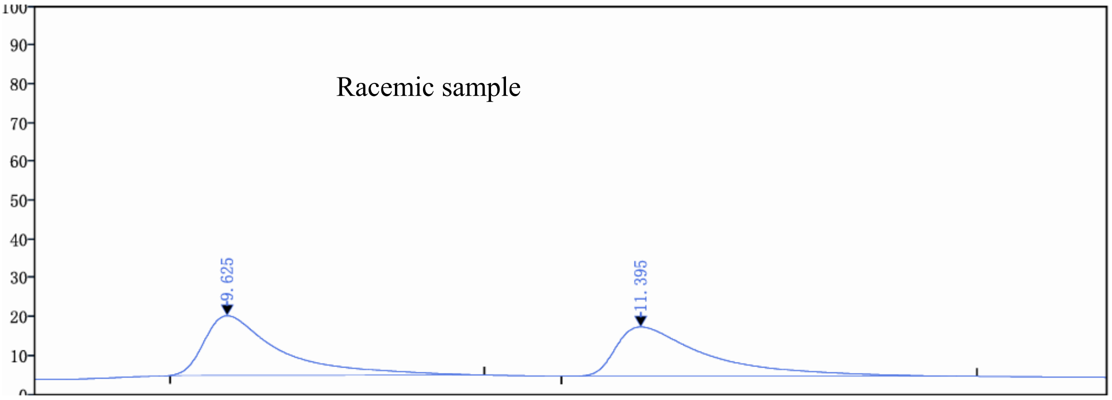

Serial Number	Type	Retention Time [min]	Peak Area	Area %
1	MM	9.625	471.37	51.00
2	MM	11.395	452.96	49.00
The Total			924.34	

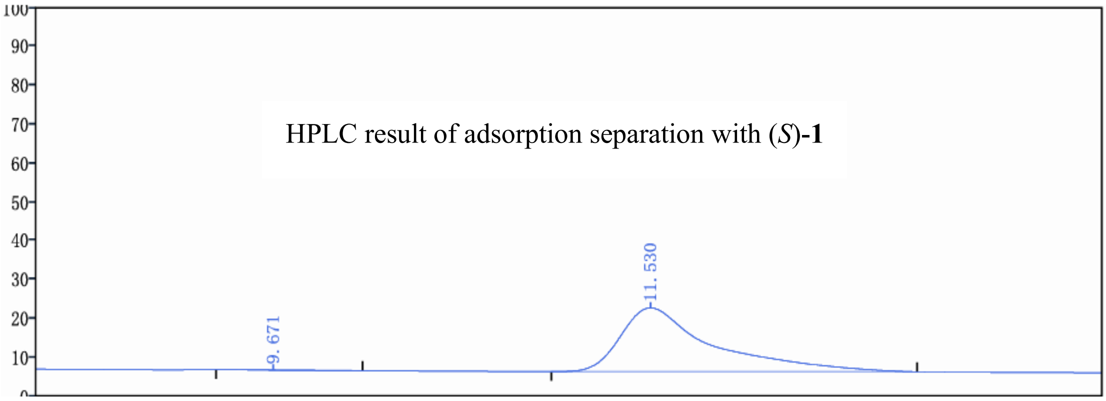

Serial Number	Type	Retention Time [min]	Peak Area	Area %
1	MM	9.671	1.23	0.18
2	BM	11.530	672.63	99.82
The Total			673.86	

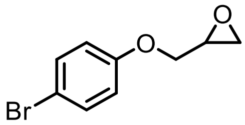

Chiral HPLC analysis: Daicel Chiralce OJ-H: hexane/*i*-PrOH =90/10; flow rate = 1 mL/min; 230 nm; t_R = 10.598 min, t_R = 13.028 min.

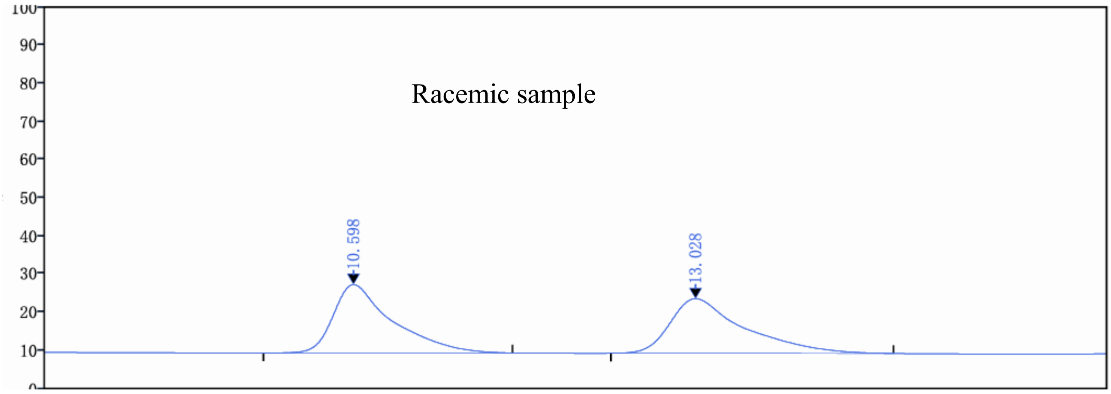

Serial Number	Type	Retention Time [min]	Peak Area	Area %
1	BM	10.598	921.61	50.03
2	MM	13.028	920.67	49.97
The Total			1842.28	

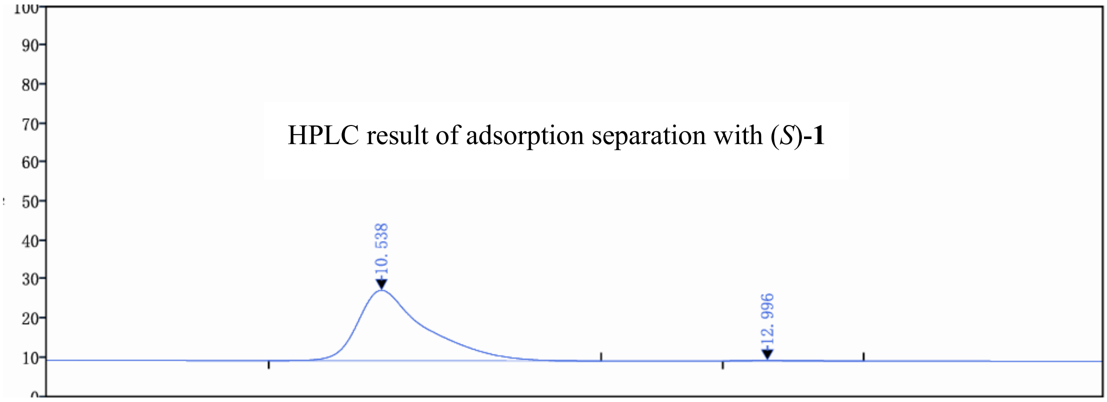

Serial Number	Type	Retention Time [min]	Peak Area	Area %
1	BB	10.538	1285.98	99.52
2	MM	12.996	6.26	0.48
The Total			1292.24	

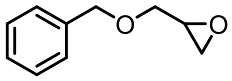

Chiral HPLC analysis: Daicel Chiralce IA: hexane/*i*-PrOH =98/2; flow rate = 1 mL/min; 220 nm; t_R = 6.396 min, t_R =6.696 min.

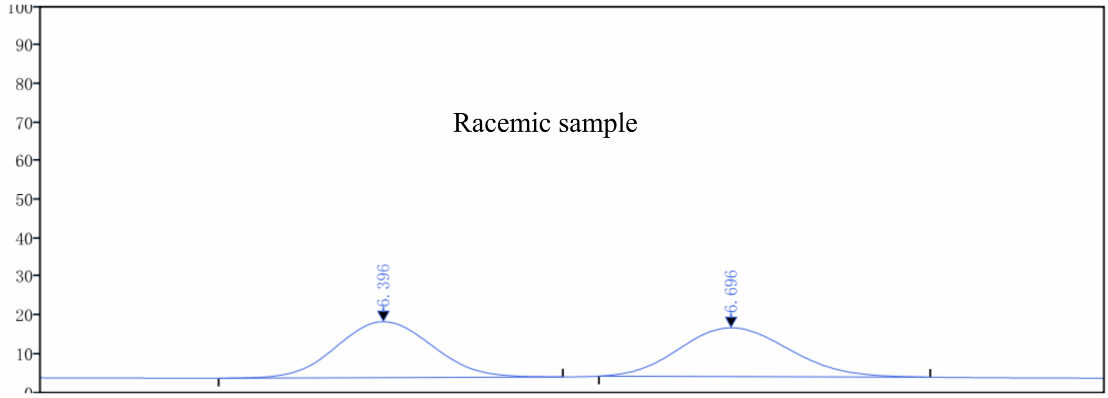

Serial Number	Type	Retention Time [min]	Peak Area	Area %
1	MM	6.396	1944.30	50.01
2	MM	6.696	1943.38	49.99
The Total			3887.68	

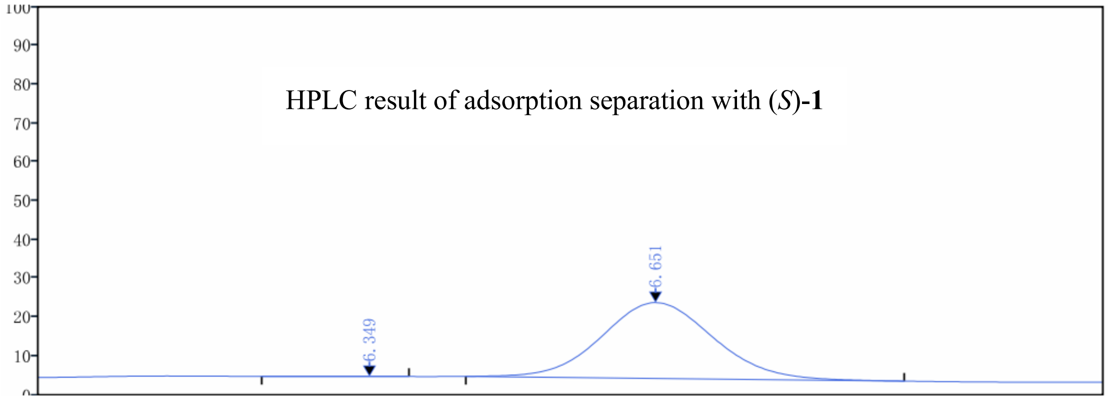

Serial Number	Type	Retention Time [min]	Peak Area	Area %
1	MM	6.349	0.77	0.02
2	MM	6.651	3543.36	99.98
The Total			3544.13	

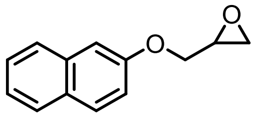

Chiral HPLC analysis: Daicel Chiralce OJ-H: hexane/*i*-PrOH =70/30; flow rate = 1 mL/min; 254 nm; t_R = 14.698 min, t_R =23.021min.

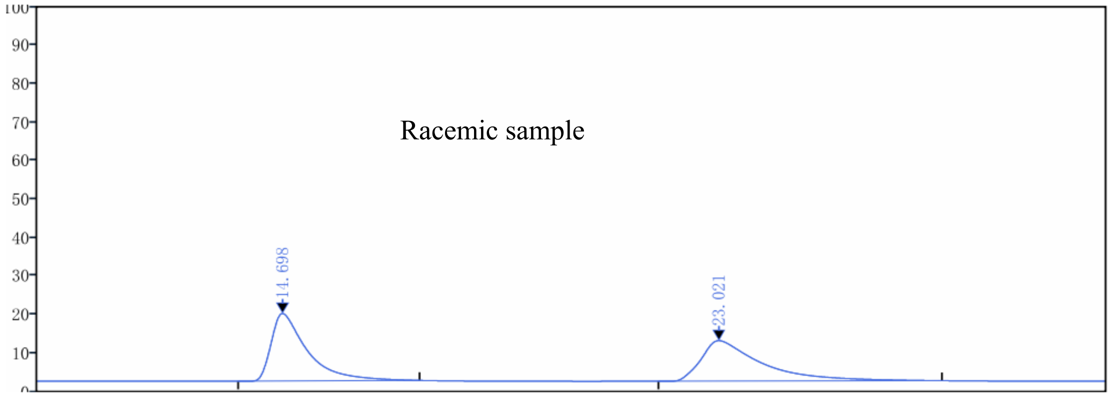

Serial Number	Type	Retention Time [min]	Peak Area	Area %
1	MM	14.698	9043.61	49.99
2	MM	23.021	9047.89	50.01
The Total			18091.50	

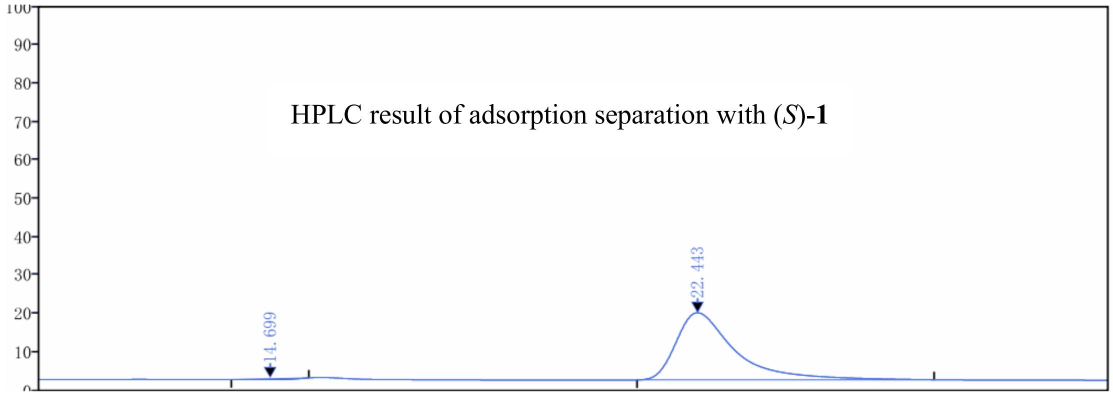

Serial Number	Type	Retention Time [min]	Peak Area	Area %
1	MM	14.699	43.12	1.08
2	HM	22.443	3940.45	98.92
The Total			3983.56	

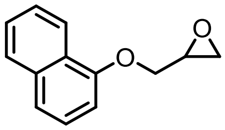

Chiral HPLC analysis: Daicel Chiralce AS-H: hexane/*i*-PrOH =98/2; flow rate = 1 mL/min; 230 nm; t_R = 9.994 min, t_R =12.114 min.

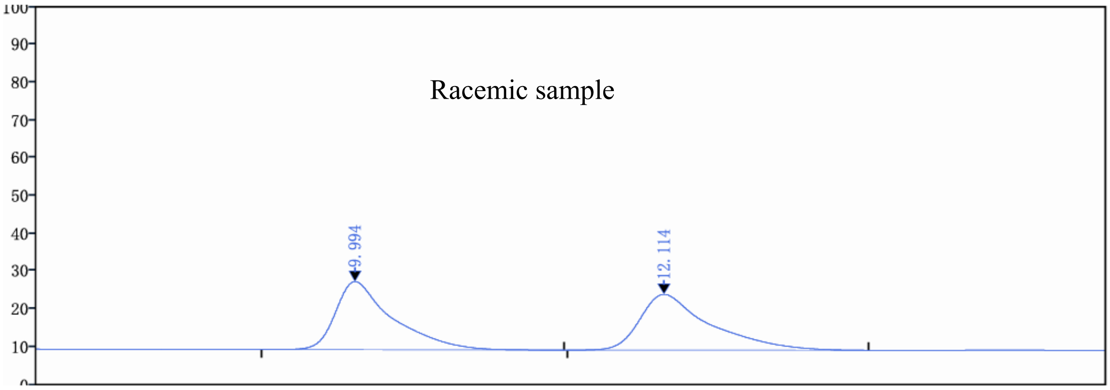

Serial Number	Type	Retention Time [min]	Peak Area	Area %
1	BB	9.994	996.83	50.00
2	BM	12.114	996.65	50.00
The Total			1993.48	

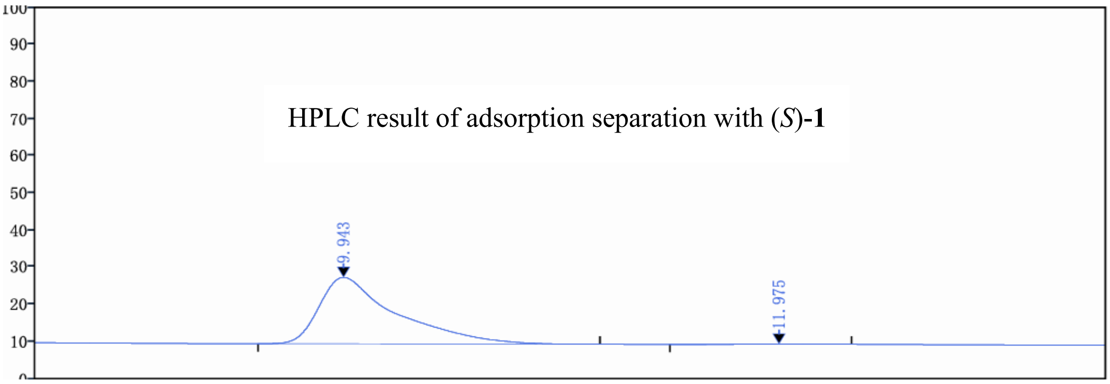

Serial Number	Type	Retention Time [min]	Peak Area	Area %
1	BB	9.943	431.77	99.60
2	MM	11.975	1.75	0.40
The Total			433.52	

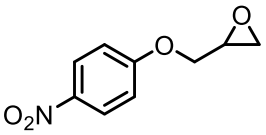

Chiral HPLC analysis: Daicel Chiralce AD-H: hexane/*i*-PrOH =90/10; flow rate = 1 mL/min; 218 nm; t_R = 14.638 min, t_R = 18.051min.

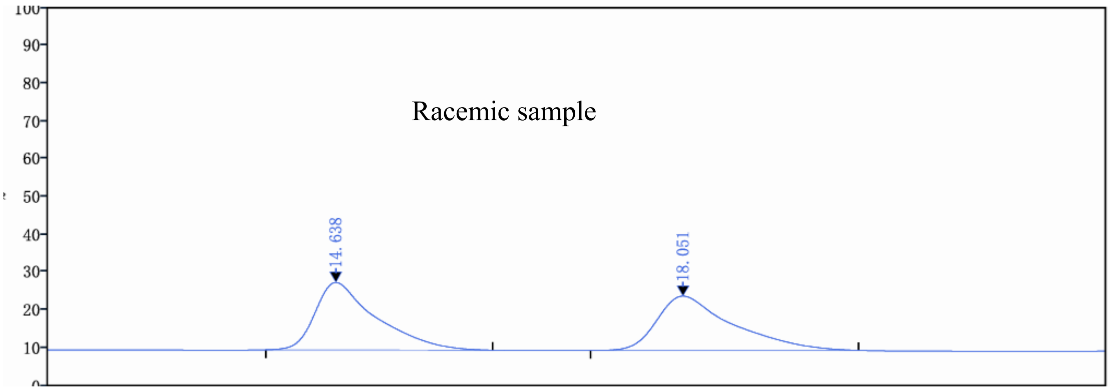

Serial Number	Type	Retention Time [min]	Peak Area	Area %
1	MM	14.638	2484.56	50.09
2	MM	18.051	2475.91	49.91
The Total			4960.47	

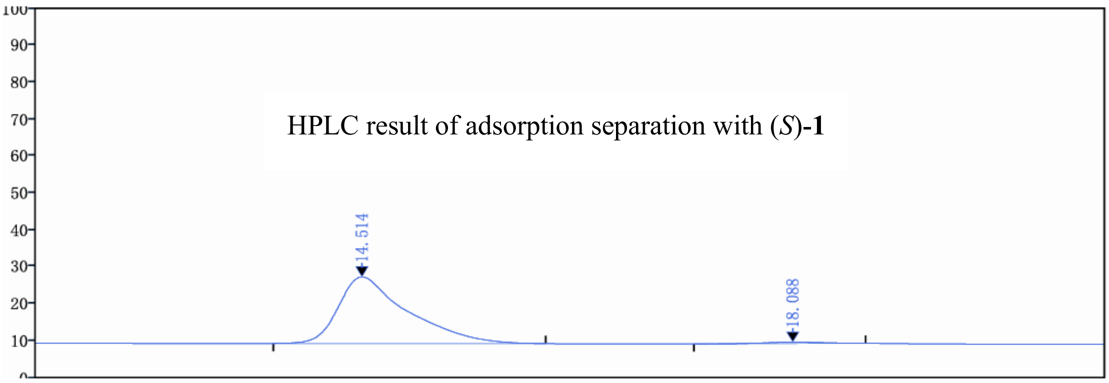

Serial Number	Type	Retention Time [min]	Peak Area	Area %
1	MM	14.514	3236.35	98.38
2	MM	18.088	5.318	1.62
The Total			3289.53	

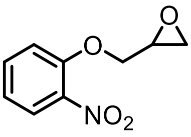

Chiral HPLC analysis: Daicel Chiralce IA: hexane/*i*-PrOH =90/10; flow rate = 1 mL/min; 220 nm; t_R = 10.050 min, t_R =12.852 min.

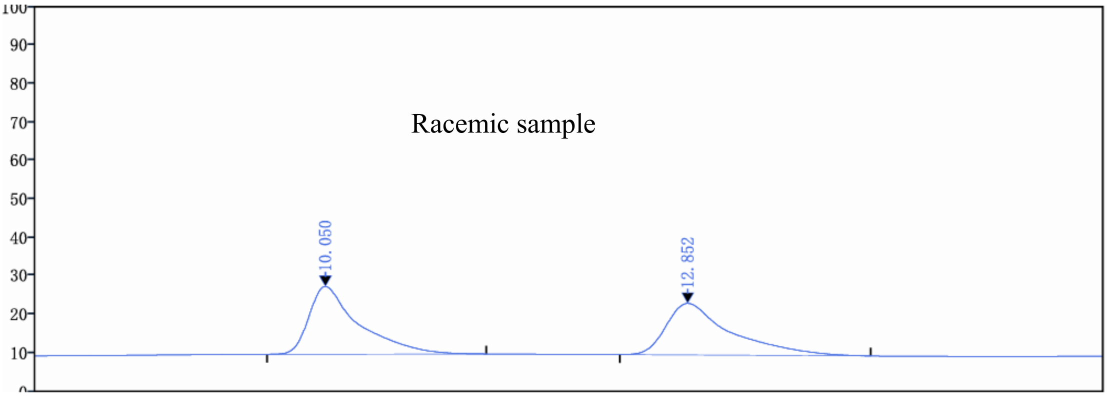

Serial Number	Type	Retention Time [min]	Peak Area	Area %
1	MM	10.050	792.92	50.39
2	BM	12.852	780.70	49.61
The Total			1573.62	

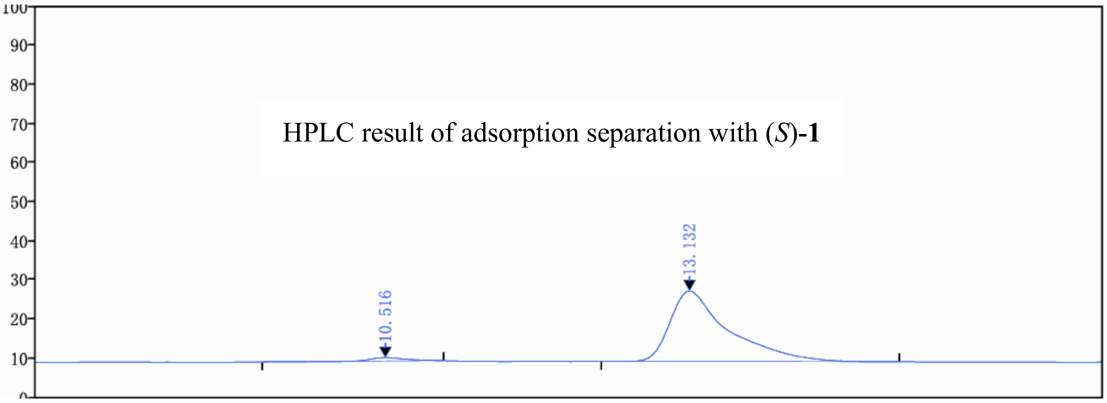

Serial Number	Type	Retention Time [min]	Peak Area	Area %
1	MM	10.516	15.44	2.51
2	BB	13.132	589.79	97.49
The Total			614.23	

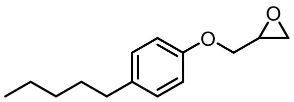

Chiral HPLC analysis: Daicel Chiralce IA: hexane/*i*-PrOH =99/1; flow rate = 0.9 mL/min; 220 nm; t_R = 5.463 min, t_R = 6.057 min.

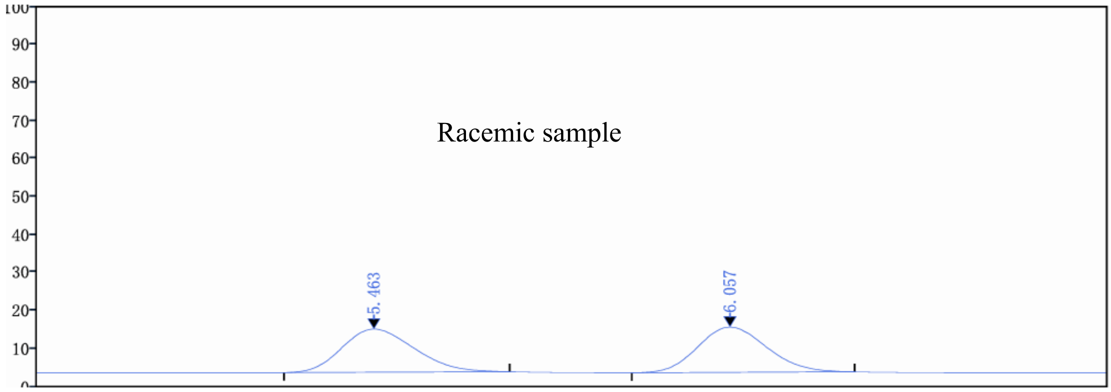

Serial Number	Type	Retention Time [min]	Peak Area	Area %
1	MM	5.463	3680.73	49.98
2	MM	6.057	3683.17	50.02
The Total			7363.91	

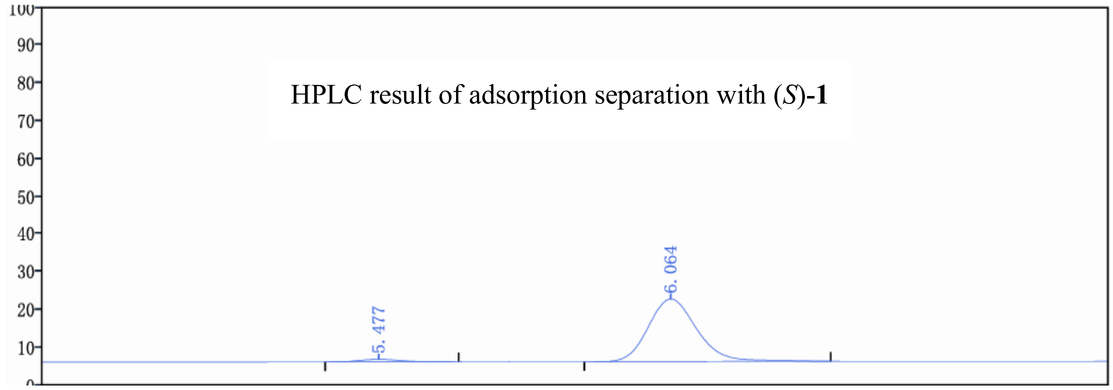

Serial Number	Type	Retention Time [min]	Peak Area	Area %
1	MM	5.477	40.03	3.58
2	MM	6.064	1079.27	96.42
The Total			1119.30	

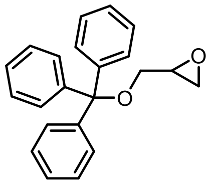

Chiral HPLC analysis: Daicel Chiralce IA: hexane/*i*-PrOH =95/5; flow rate = 1.0 mL/min; 220 nm; t_R = 11.148 min, t_R = 13.440 min.

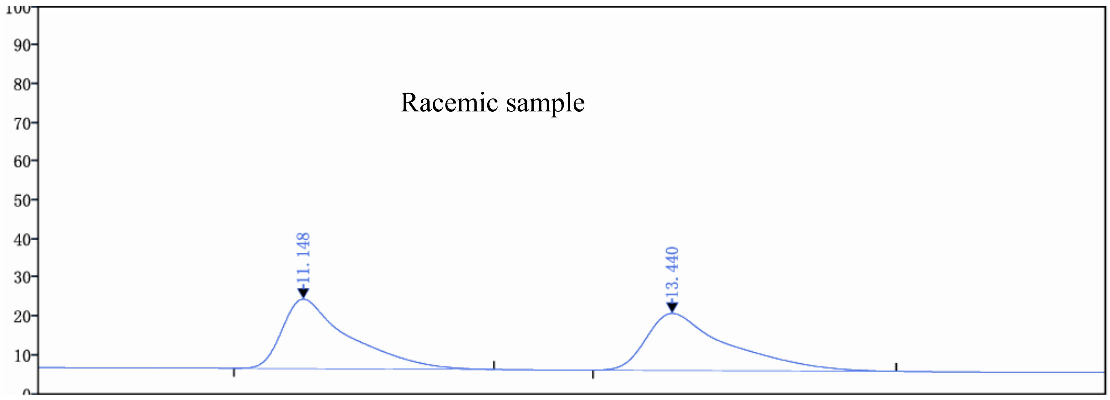

Serial Number	Type	Retention Time [min]	Peak Area	Area %
1	BM	11.148	733.95	50.02
2	MM	13.440	733.45	49.98
The Total			1573.62	

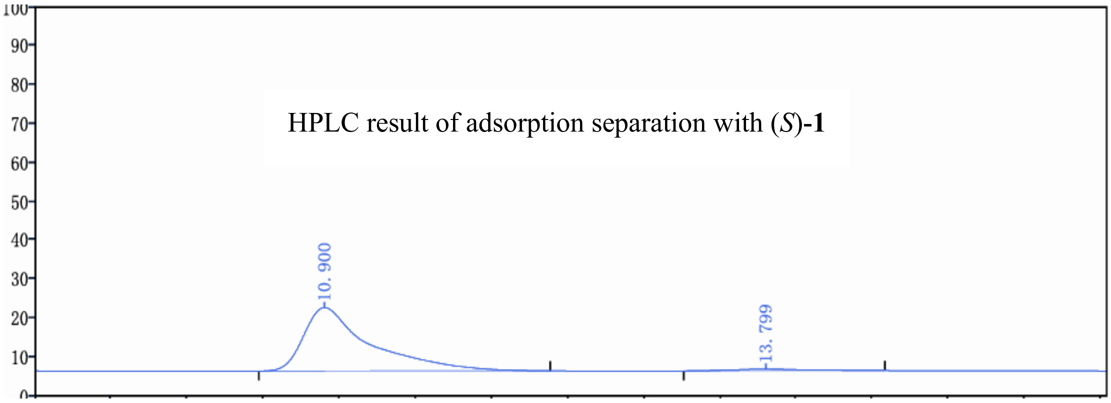

Serial Number	Type	Retention Time [min]	Peak Area	Area %
1	MM	10.900	797.05	97.34
2	MM	13.799	21.77	2.66
The Total			818.82	

16. Figure S13. HPLC spectra for the cycling enantioseparation.

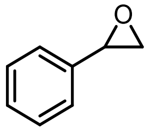

Chiral HPLC analysis: Daicel Chiralce OD-H; hexane/*i*-PrOH = 95/5, flow rate = 1 mL/min, 220 nm, t_R = 5.451 min, t_R = 6.083 min.

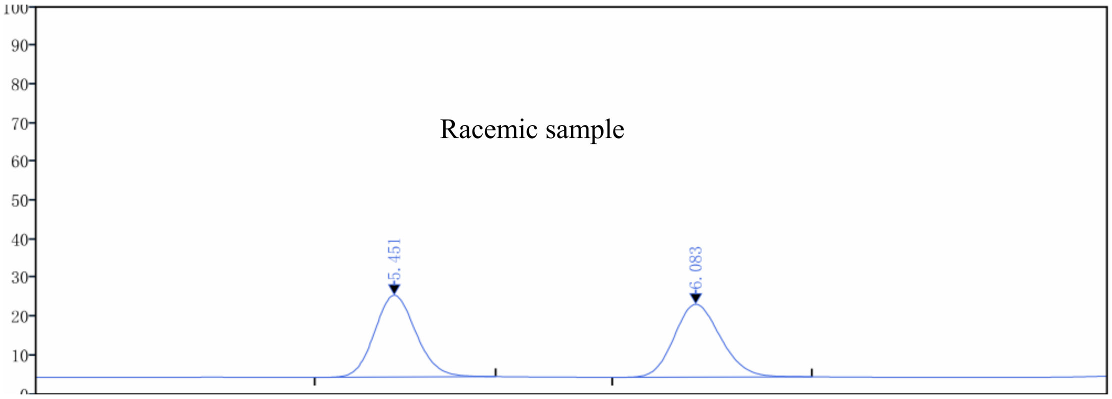

Serial Number	Type	Retention Time [min]	Peak Area	Area %
1	MM	5.451	1597.55	50.02
2	MM	6.083	1596.26	49.98
The Total			3193.81	

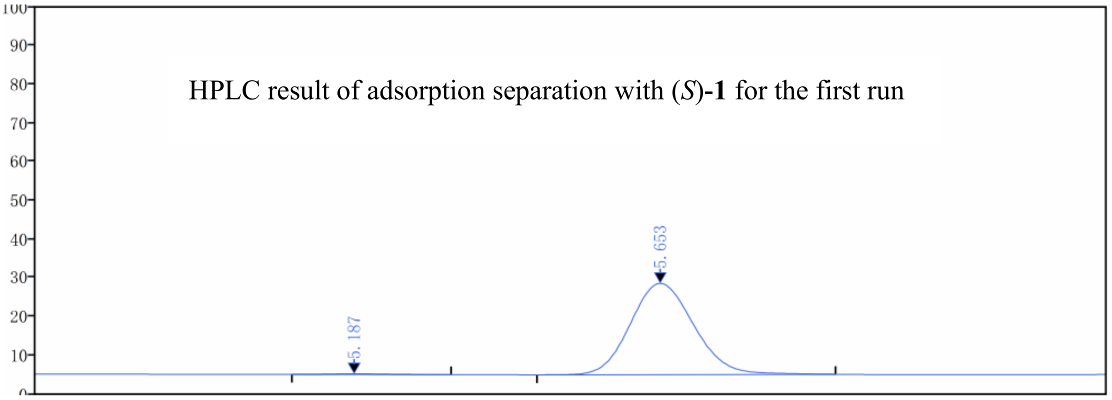

Serial Number	Type	Retention Time [min]	Peak Area	Area %
1	BM	5.187	12.04	0.71
2	BM	5.653	1680.23	99.29
The Total			1692.27	

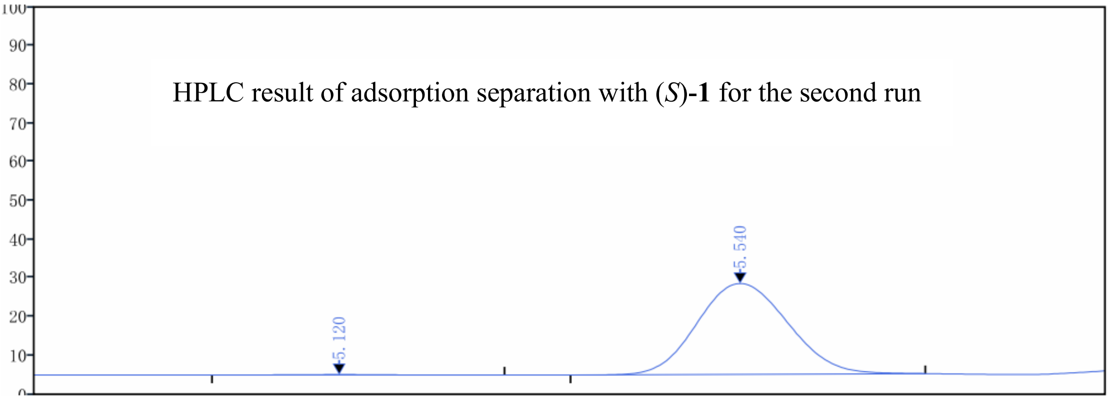

Serial Number	Type	Retention Time [min]	Peak Area	Area %
1	MB	5.120	11.97	0.53
2	BM	5.540	2229.24	99.47
The Total			2241.21	

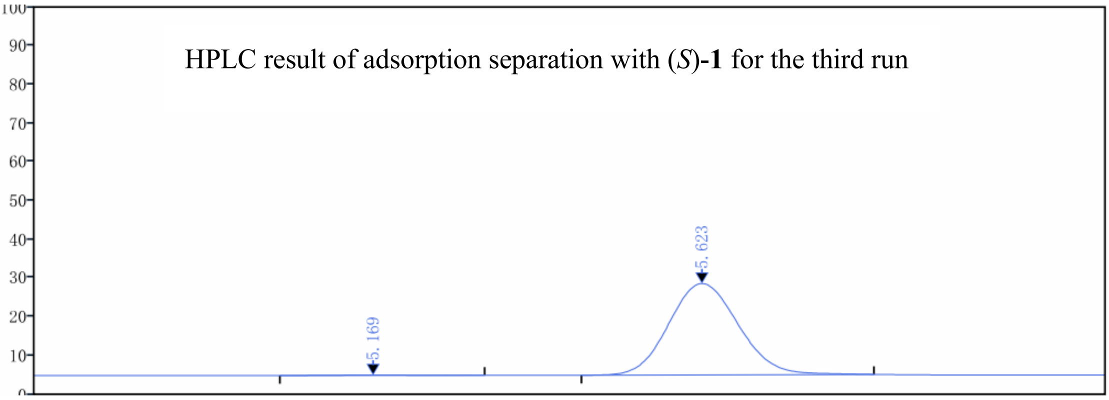

Serial Number	Type	Retention Time [min]	Peak Area	Area %
1	BB	5.169	7.56	0.50
2	MM	5.623	1509.93	99.50
The Total			1517.49	

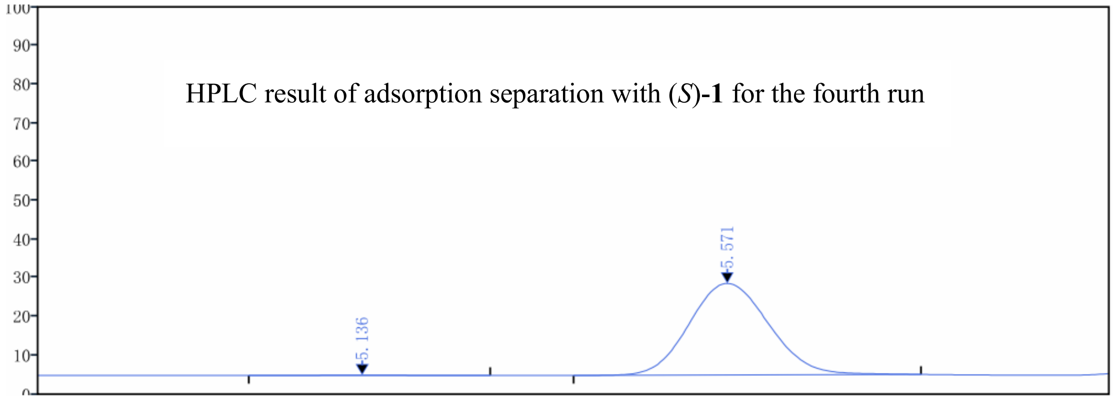

Serial Number	Type	Retention Time [min]	Peak Area	Area %
1	BB	5.136	8.61	0.47
2	MM	5.571	1809.27	99.53
The Total			1817.88	

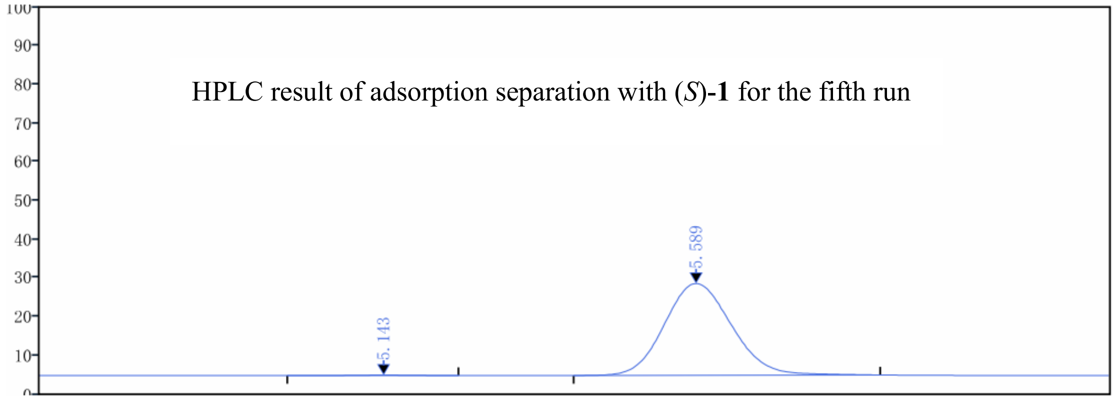

Serial Number	Type	Retention Time [min]	Peak Area	Area %
1	BM	5.143	6.35	0.46
2	MM	5.589	1359.18	99.54
The Total			1365.53	

17. Figure S14. HPLC spectra of *β*-nitroalcohols (in Figure 4).

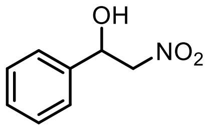

Chiral HPLC analysis: Daicel Chiralce OD; hexane/*i*-PrOH = 90/10, flow rate = 1 mL/min, 220 nm, t_R = 14.210 min, t_R = 16.706 min.

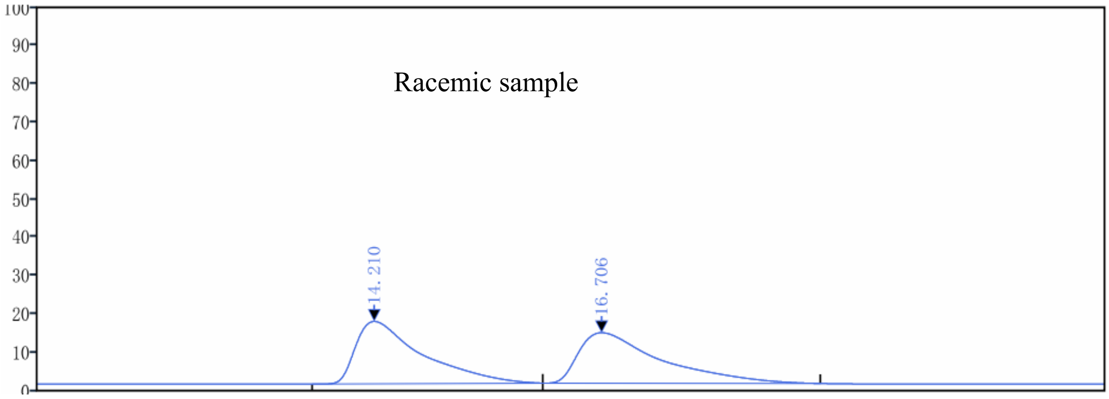

Serial Number	Type	Retention Time [min]	Peak Area	Area %
1	MM	14.210	79385.84	49.98
2	MM	16.706	79437.06	50.02
The Total			158822.90	

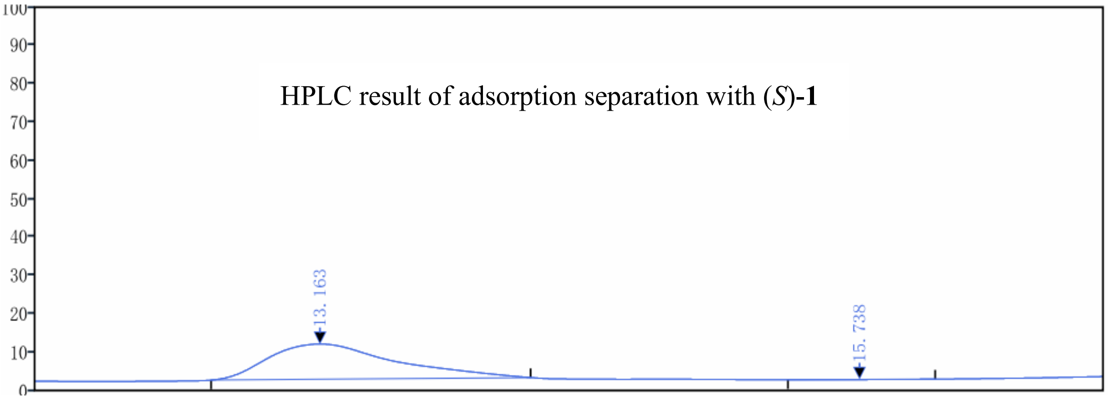

Serial Number	Type	Retention Time [min]	Peak Area	Area %
1	MM	13.163	1981.27	99.69
2	MM	15.738	6.17	0.31
The Total			1987.44	

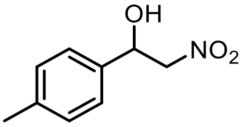

Chiral HPLC analysis: Daicel Chiralce OD; hexane/*i*-PrOH = 90/10, flow rate = 1 mL/min, 220 nm, t_R = 17.732 min, t_R = 23.264 min.

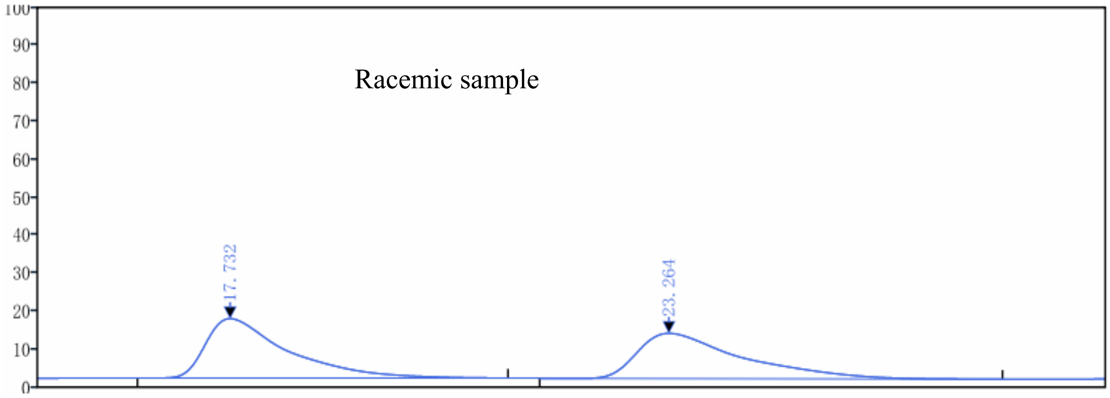

Serial Number	Type	Retention Time [min]	Peak Area	Area %
1	MM	17.732	9834.21	50.84
2	MM	23.264	9508.42	49.16
The Total			19342.63	

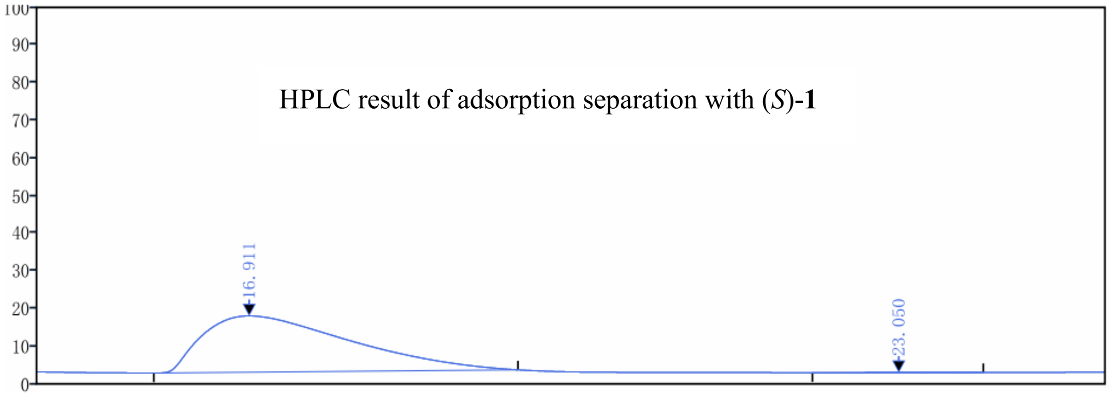

Serial Number	Type	Retention Time [min]	Peak Area	Area %
1	MM	16.911	6936.41	99.90
2	MM	23.050	6.72	0.10
The Total			6943.13	

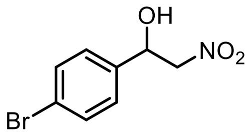

Chiral HPLC analysis: Daicel Chiralce OD; hexane/*i*-PrOH = 90/10, flow rate = 1 mL/min, 220 nm, t_R = 26.974 min, t_R = 36.723 min.

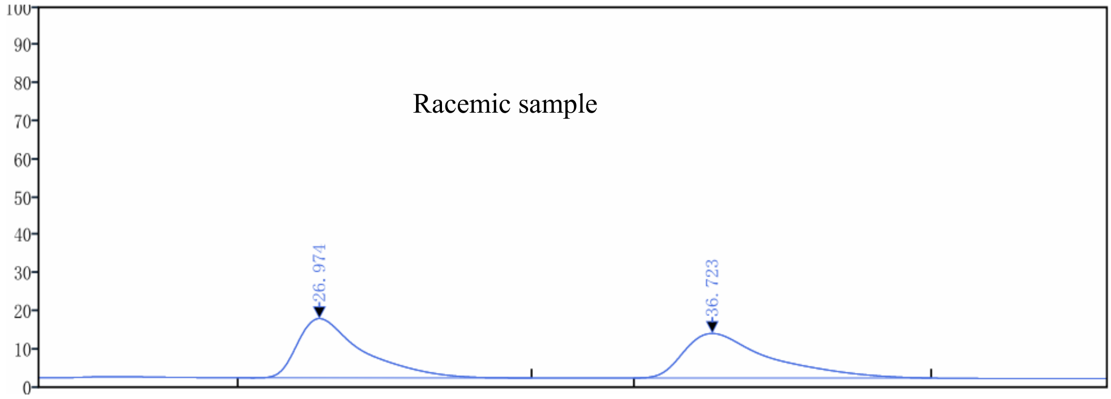

Serial Number	Type	Retention Time [min]	Peak Area	Area %
1	MM	26.974	10592.73	50.23
2	MM	36.723	10497.56	49.77
The Total			21090.29	

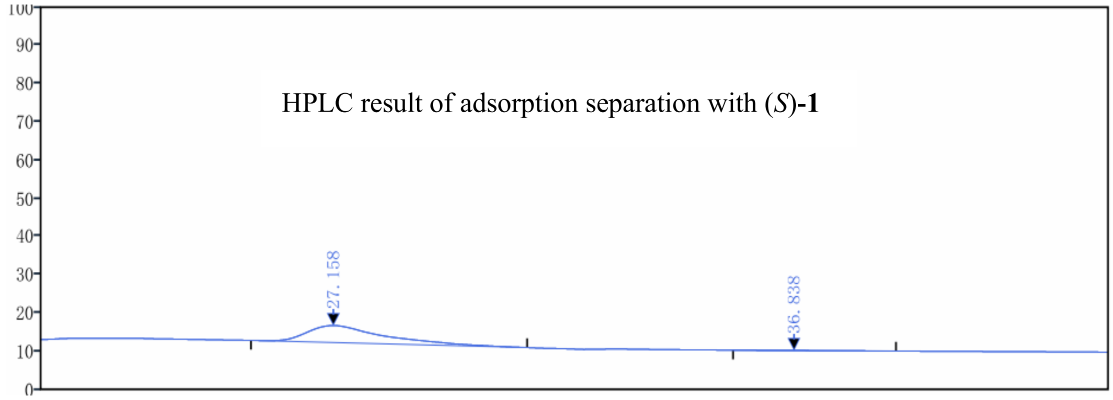

Serial Number	Type	Retention Time [min]	Peak Area	Area %
1	MM	27.158	450.34	99.72
2	MM	36.838	1.26	0.28
The Total			451.60	

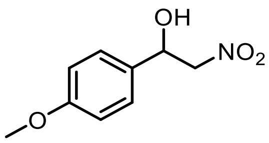

Chiral HPLC analysis: Daicel Chiralce OD; hexane/*i*-PrOH = 90/10, flow rate = 1 mL/min, 220 nm, t_R = 20.384 min, t_R = 26.591 min.

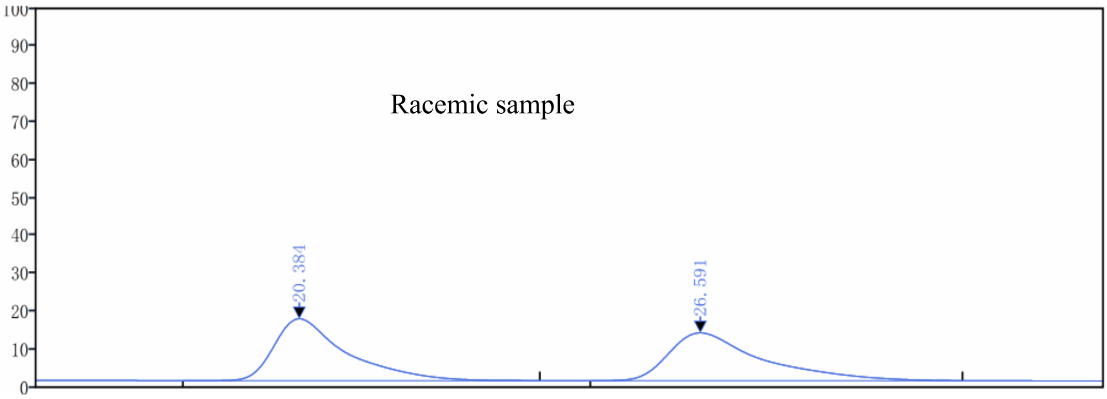

Serial Number	Type	Retention Time [min]	Peak Area	Area %
1	MM	20.384	13985.10	50.15
2	MM	26.591	13902.20	49.85
The Total			27887.30	

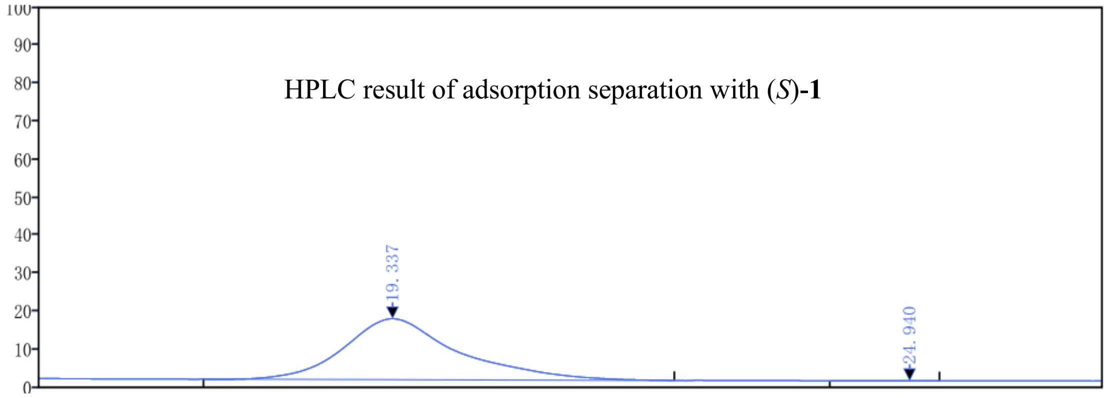

Serial Number	Type	Retention Time [min]	Peak Area	Area %
1	MM	19.337	547.68	99.99
2	MM	24.940	0.02	0.01
The Total			547.70	

18. Figure S15. HPLC spectra of mandelate derivatives (in Figure 4).

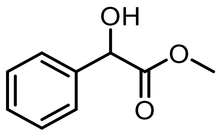

Chiral HPLC analysis: Daicel Chiralce OD; hexane/*i*-PrOH = 90/10, flow rate = 1 mL/min, 220 nm, t_R = 8.912 min, t_R = 14.737 min.

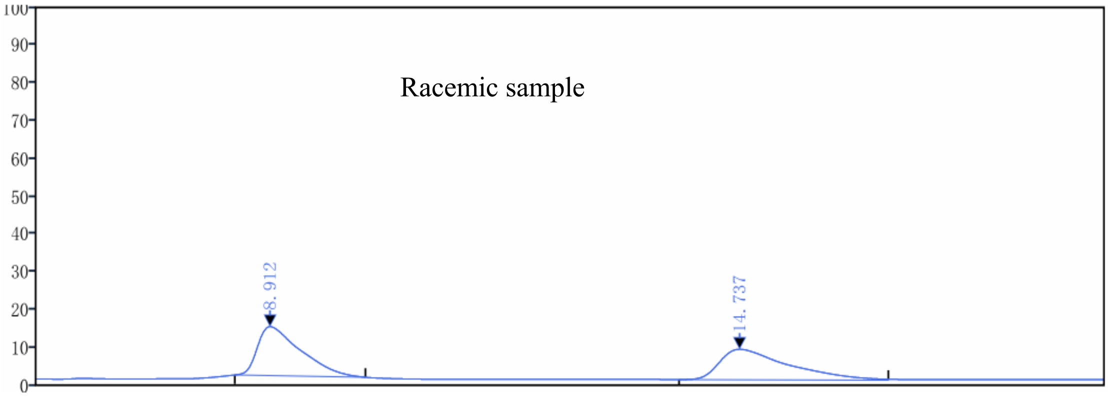

Serial Number	Type	Retention Time [min]	Peak Area	Area %
1	MM	8.912	5765.87	50.39
2	MM	14.737	5677.75	49.61
The Total			11443.62	

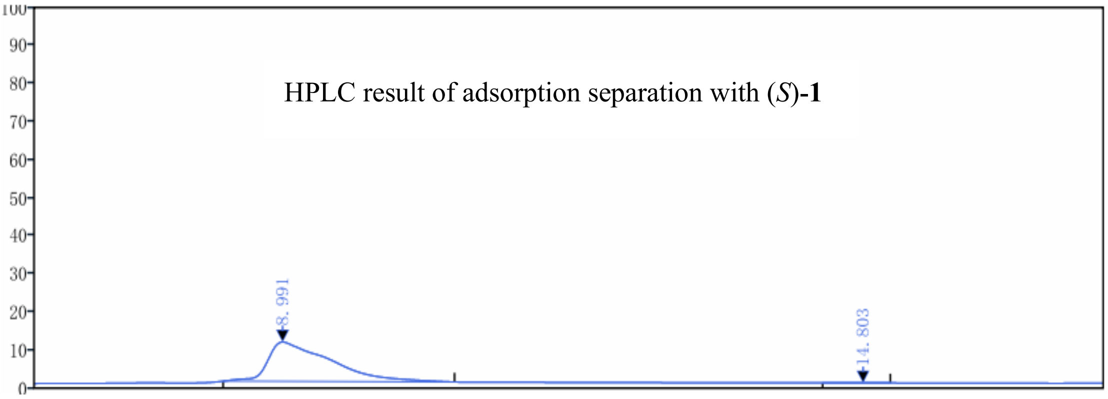

Serial Number	Type	Retention Time [min]	Peak Area	Area %
1	MM	8.991	1184.22	100.00
2	MM	14.803	0.01	0.00
The Total			1184.23	

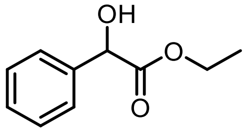

Chiral HPLC analysis: Daicel Chiralce OD; hexane/*i*-PrOH = 97/3, flow rate = 1 mL/min, 220 nm, t_R = 12.789 min, t_R = 22.219 min.

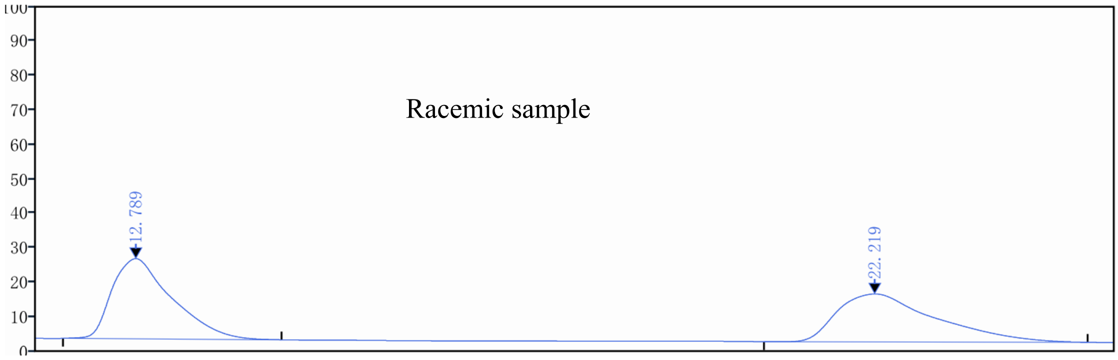

Serial Number	Type	Retention Time [min]	Peak Area	Area %
1	MM	12.789	4464.28	50.53
2	MM	22.219	4370.96	49.47
The Total			8835.24	

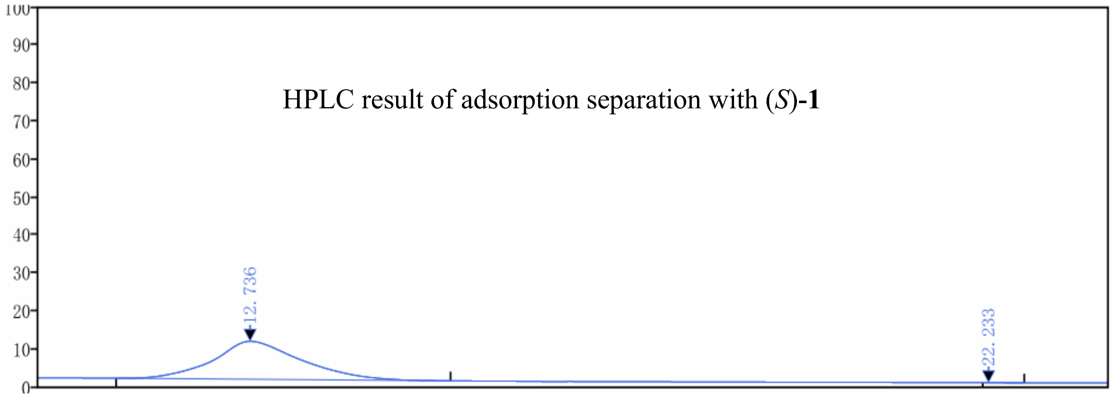

Serial Number	Type	Retention Time [min]	Peak Area	Area %
1	MM	12.736	392.78	99.99
2	MM	22.233	0.01	0.01
The Total			392.79	

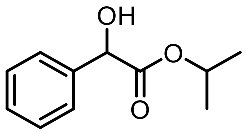

Chiral HPLC analysis: Daicel Chiralce OD; hexane/*i*-PrOH = 90/10, flow rate = 1 mL/min, 220 nm, t_R = 9.711 min, t_R = 16.512 min.

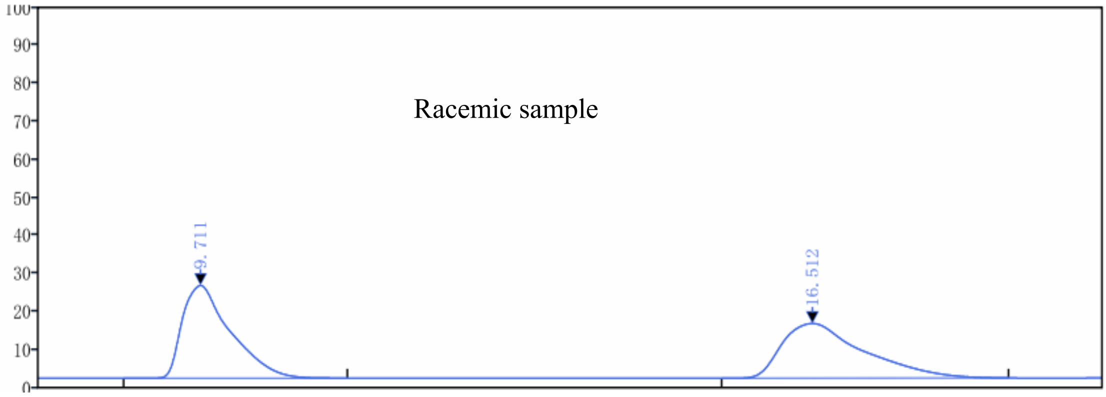

Serial Number	Type	Retention Time [min]	Peak Area	Area %
1	MM	9.711	17492.90	49.91
2	MM	16.512	17556.45	50.09
The Total			35049.35	

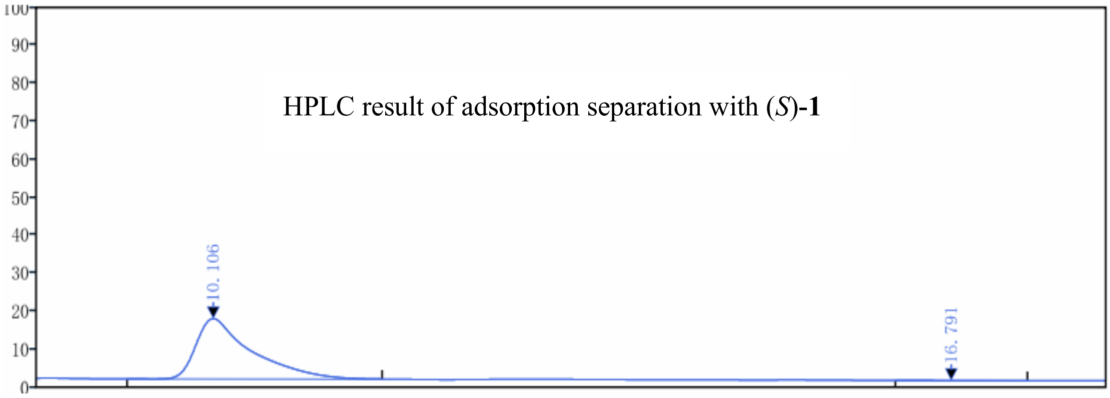

Serial Number	Type	Retention Time [min]	Peak Area	Area %
1	MM	10.106	921.14	99.91
2	MM	16.791	0.78	0.09
The Total			921.92	

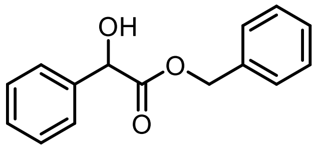

Chiral HPLC analysis: Daicel Chiralce OD; hexane/*i*-PrOH = 90/10, flow rate = 1 mL/min, 220 nm, t_R = 13.040 min, t_R = 15.178 min.

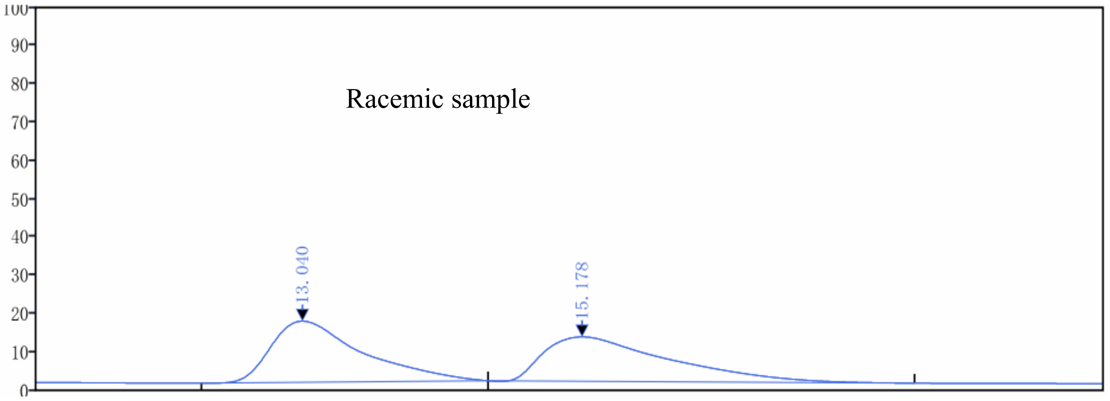

Serial Number	Type	Retention Time [min]	Peak Area	Area %
1	MM	13.040	1771.86	50.13
2	MM	15.178	1762.95	49.87
The Total			3534.81	

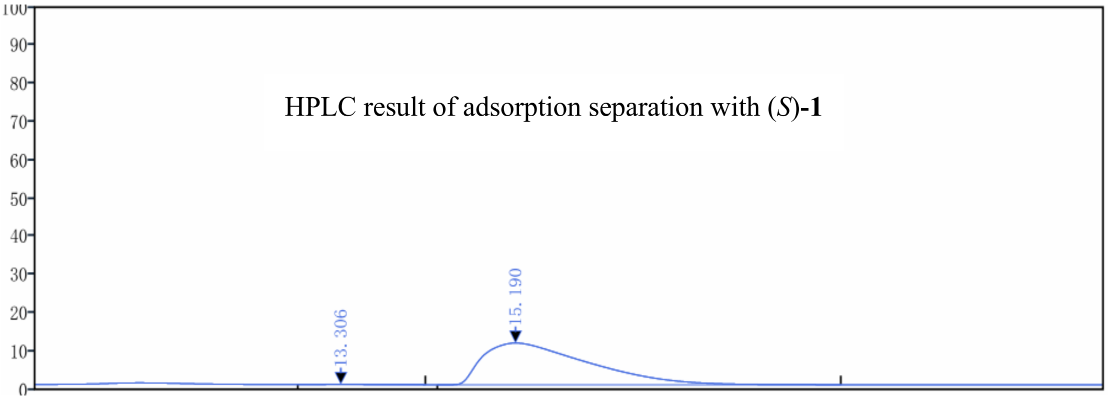

Serial Number	Type	Retention Time [min]	Peak Area	Area %
1	MM	13.306	54.04	0.45
2	MM	15.190	12078.75	99.55
The Total			12132.79	

19. Figure S16. HPLC spectra of secondary alcohols (in Figure 4).

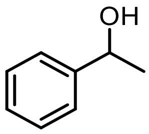

Chiral HPLC analysis: Daicel Chiralce OD: hexane/*i*-PrOH =95/3; flow rate =1 mL/min; 220 nm; t_R = 10.643 min, t_R = 13.113 min.

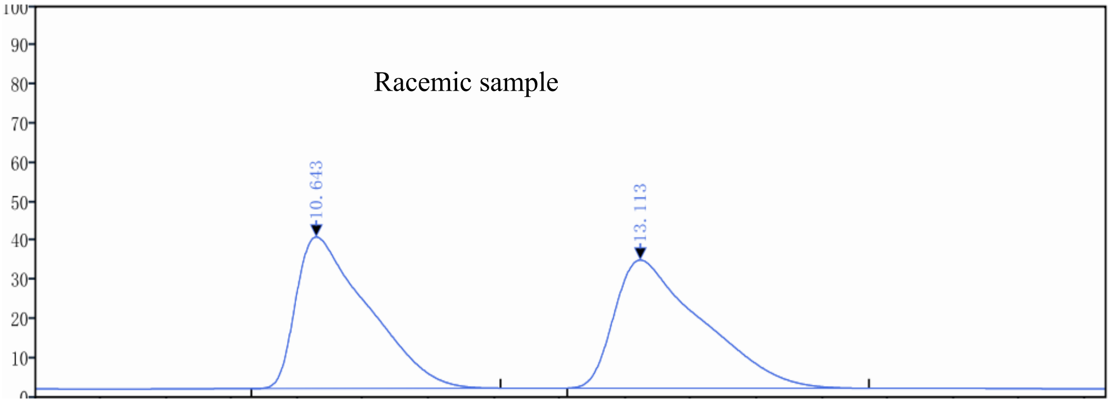

Serial Number	Type	Retention Time [min]	Peak Area	Area %
1	MM	10.643	7563.10	49.26
2	MM	13.113	7789.23	50.74
The Total			15352.33	

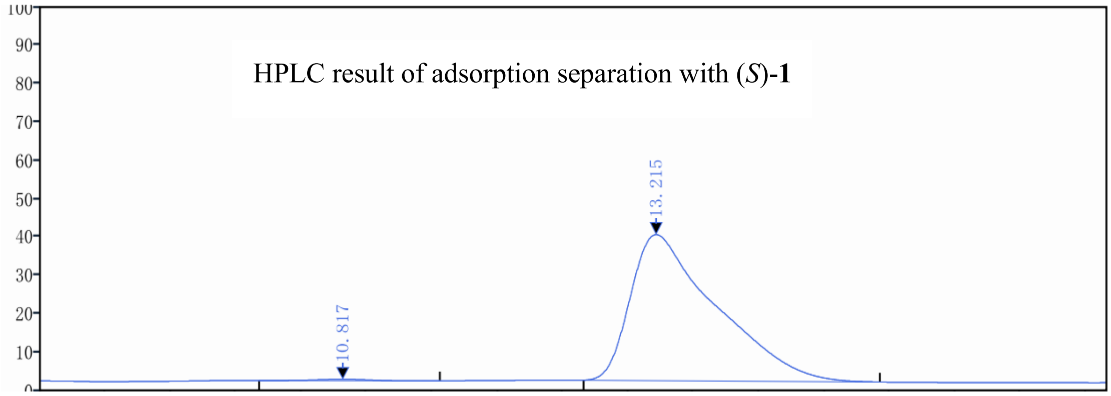

Serial Number	Type	Retention Time [min]	Peak Area	Area %
1	MM	10.817	18.10	0.49
2	MM	13.215	3694.84	99.51
The Total			3712.94	

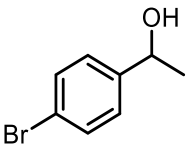

Chiral HPLC analysis: Daicel Chiralce OD: hexane/*i*-PrOH =95/2; flow rate = 0.85 mL/min; 220 nm; t_R = 26.780 min, t_R = 28.907 min.

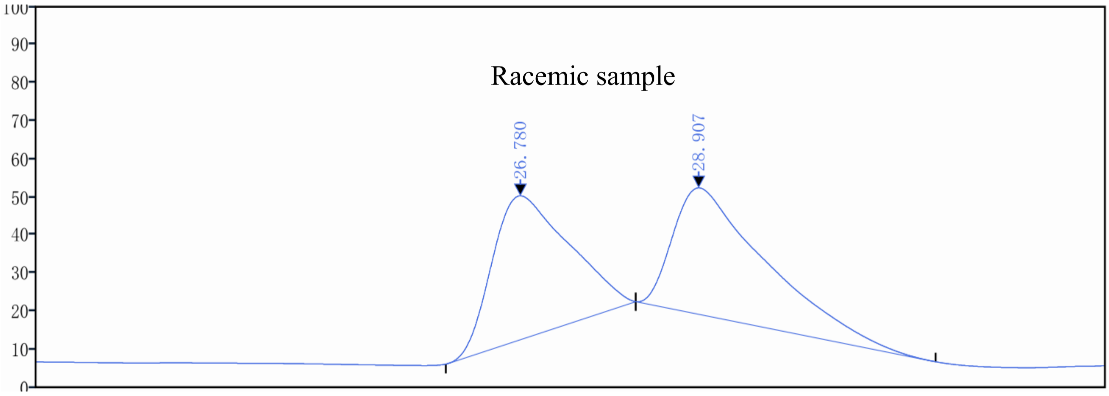

Serial Number	Type	Retention Time [min]	Peak Area	Area %
1	MM	26.780	2629.70	47.78
2	MM	28.907	2874.41	52.22
The Total			5504.11	

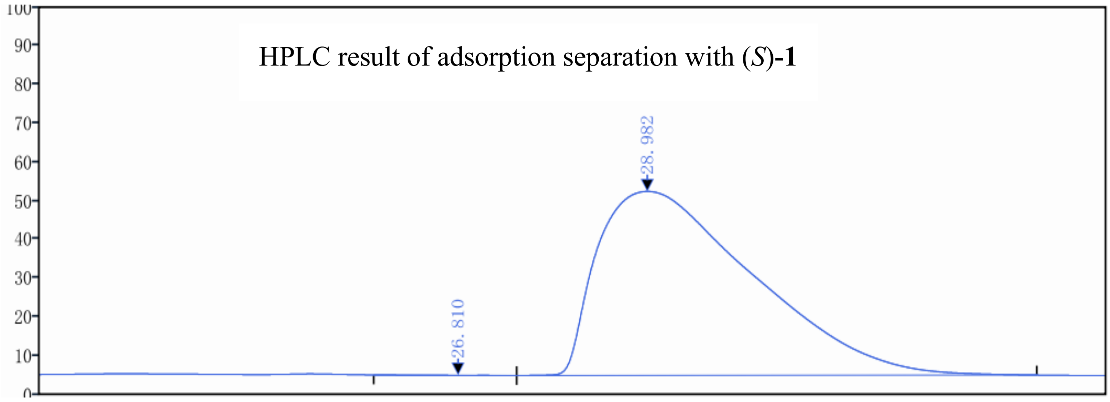

Serial Number	Type	Retention Time [min]	Peak Area	Area %
1	MM	26.810	3.28	0.04
2	MM	28.982	8410.24	99.96
The Total			8413.52	

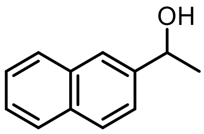

Chiral HPLC analysis: Daicel Chiralce OJ-H: hexane/*i*-PrOH =90/10; flow rate =1 mL/min; 220 nm; t_R = 12.045 min, t_R = 15.434 min.

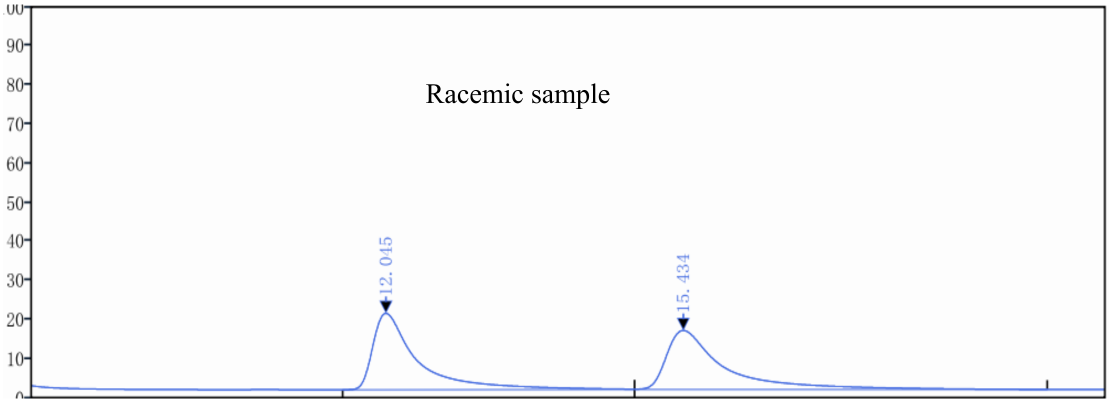

Serial Number	Type	Retention Time [min]	Peak Area	Area %
1	BM	12.045	40496.01	50.04
2	MM	15.434	40432.02	49.96
The Total			80928.03	

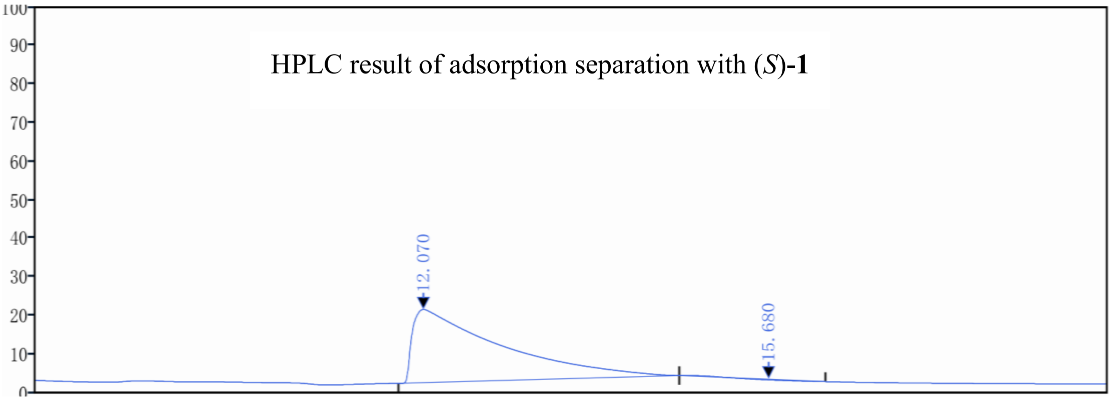

Serial Number	Type	Retention Time [min]	Peak Area	Area %
1	MM	12.070	9825.84	99.63
2	MM	15.680	37.07	0.38
The Total			9862.51	

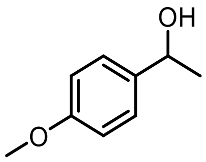

Chiral HPLC analysis: Daicel Chiralce OD: hexane/*i*-PrOH =95/5; flow rate =1 mL/min; 220 nm; t_R = 13.475 min, t_R = 14.500 min.

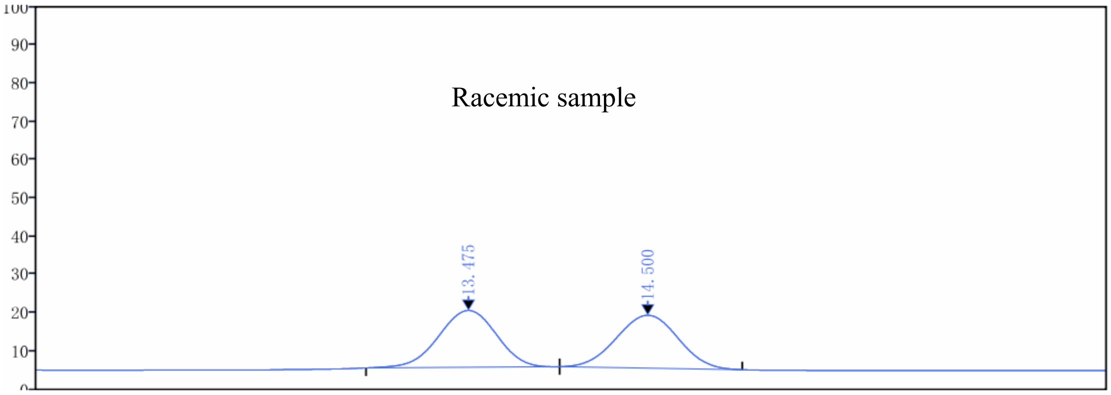

Serial Number	Type	Retention Time [min]	Peak Area	Area %
1	MM	13.475	2018.40	50.05
2	MM	14.500	2014.61	49.95
The Total			4033.01	

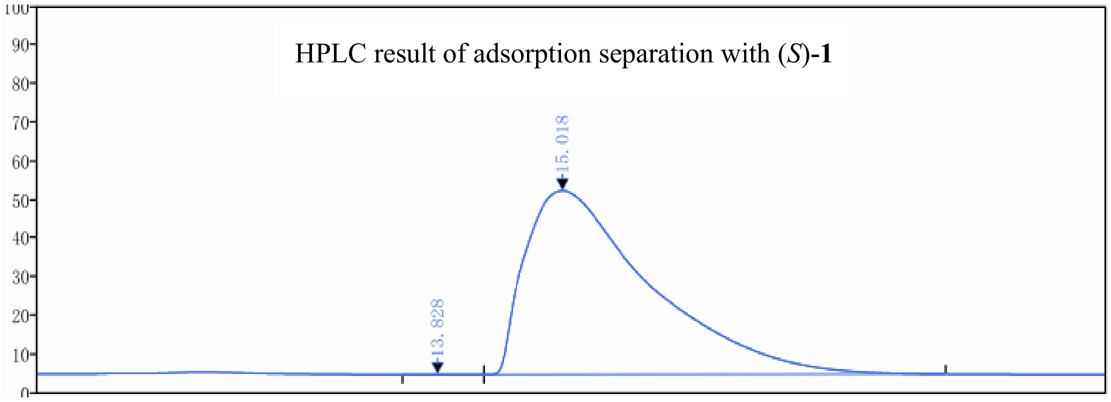

Serial Number	Type	Retention Time [min]	Peak Area	Area %
1	MM	13.828	1.71	0.00
2	MM	15.018	49114.75	100.00
The Total			49116.45	

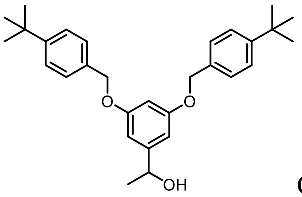

Chiral HPLC analysis: Daicel Chiralce OD: hexane/*i*-PrOH =95/5; flow rate =1 mL/min; 220 nm; t_R = 14.235 min, t_R = 15.459 min.

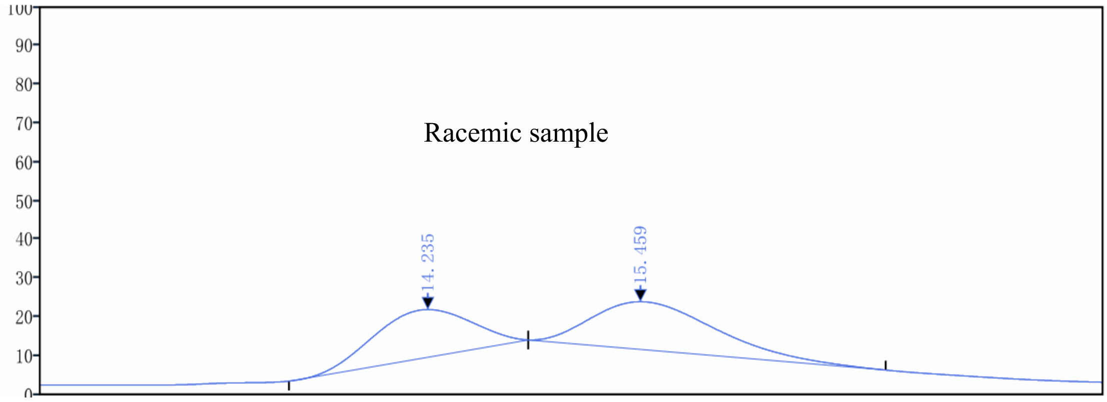

Serial Number	Type	Retention Time [min]	Peak Area	Area %
1	MM	14.235	3023.52	44.05
2	MM	15.459	3840.12	55.95
The Total			6863.64	

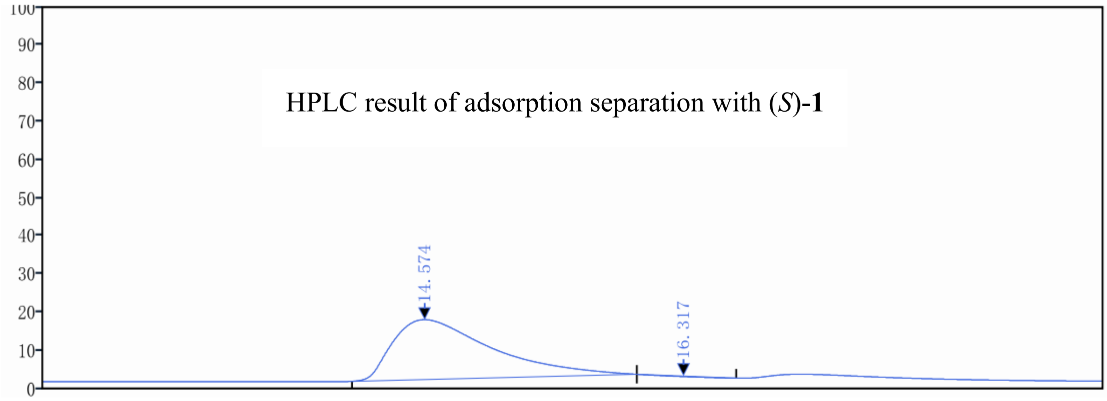

Serial Number	Type	Retention Time [min]	Peak Area	Area %
1	MM	14.574	4814.77	99.54
2	MM	16.317	22.11	0.46
The Total			4836.88	

20. Figure S17. HPLC spectra of indolin-3-ones (in Figure 4).

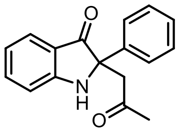

Chiral HPLC analysis: Daicel Chiralce OD; hexane/*i*-PrOH = 85/15, flow rate = 1 mL/min, 220 nm, t_R = 14.956 min, t_R = 17.851 min.

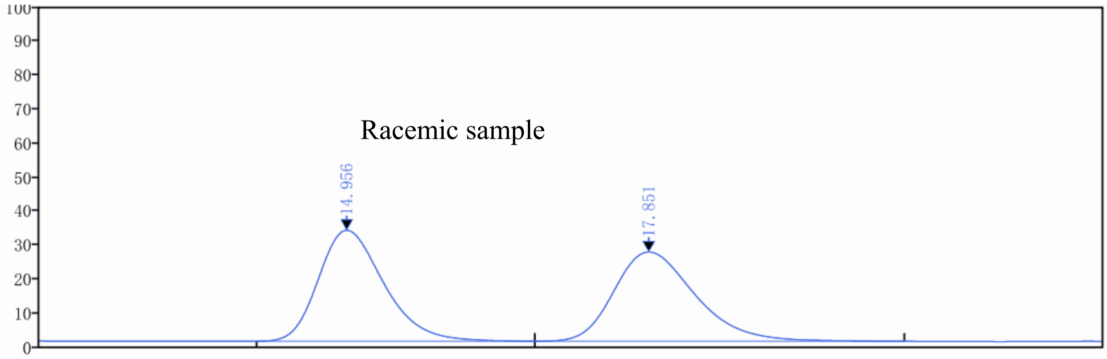

Serial Number	Type	Retention Time [min]	Peak Area	Area %
1	MM	14.956	3547.17	50.00
2	MM	17.851	3547.22	50.00
The Total			7094.39	

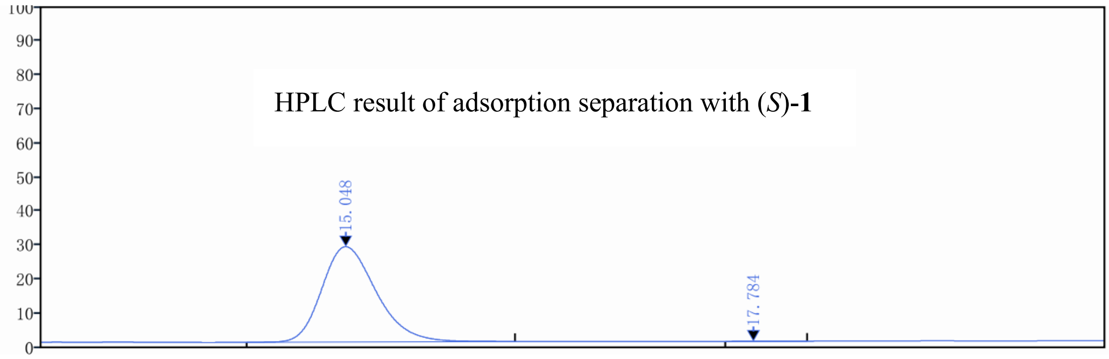

Serial Number	Type	Retention Time [min]	Peak Area	Area %
1	MM	15.048	1260.02	99.95
2	MM	17.784	0.65	0.05
The Total			1260.67	

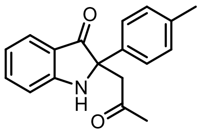

Chiral HPLC analysis: Daicel Chiralce OD; hexane/*i*-PrOH = 80/20, flow rate = 1 mL/min, 220 nm, t_R = 9.822 min, t_R = 12.647 min.

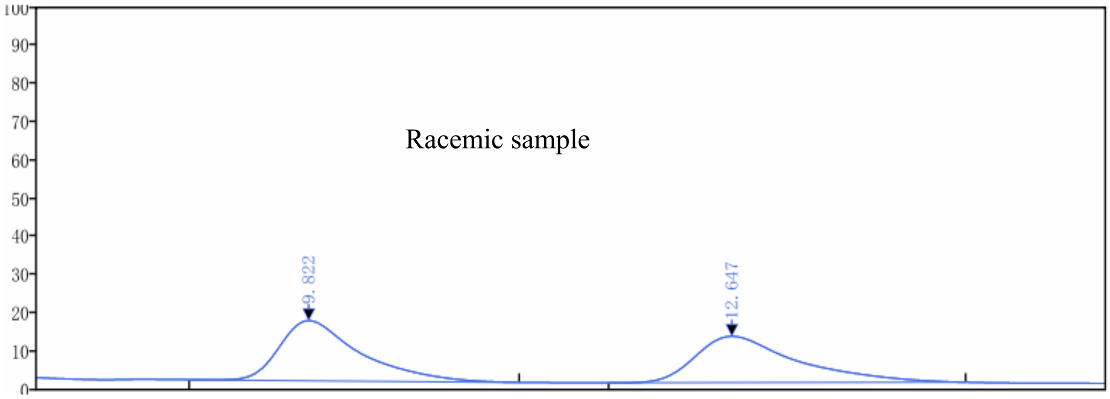

Serial Number	Type	Retention Time [min]	Peak Area	Area %
1	MM	9.822	1321.37	50.55
2	MM	12.647	1292.48	49.45
The Total			2613.85	

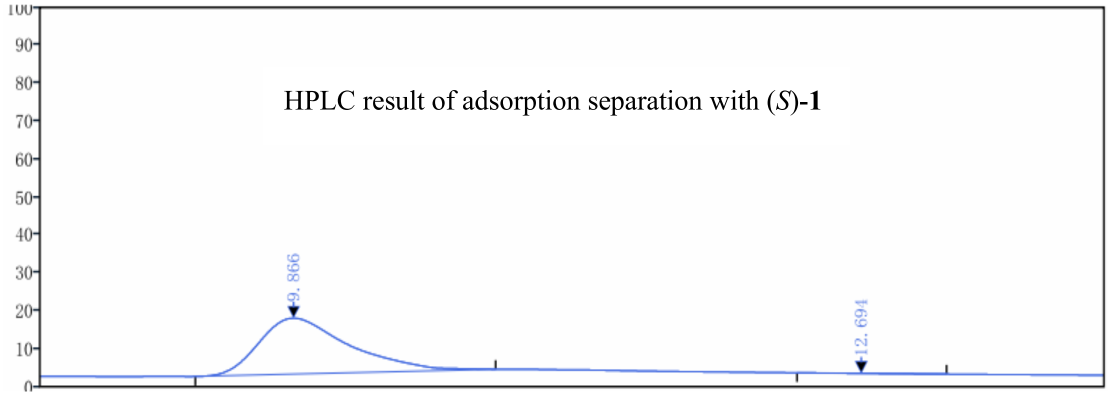

Serial Number	Type	Retention Time [min]	Peak Area	Area %
1	MM	9.866	654.46	99.95
2	MM	12.694	0.35	0.05
The Total			654.81	

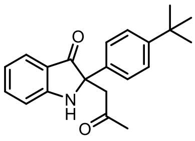

Chiral HPLC analysis: Daicel Chiralce OD; hexane/*i*-PrOH = 80/20, flow rate = 1 mL/min, 220 nm, t_R = 6.439 min, t_R = 11.120 min.

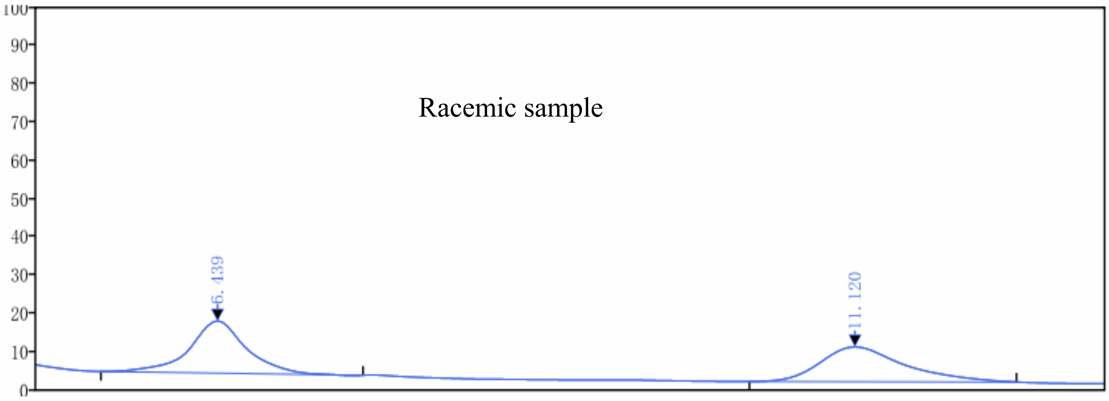

Serial Number	Type	Retention Time [min]	Peak Area	Area %
1	MM	6.439	690.48	50.29
2	BM	11.120	682.64	49.71
The Total			1373.12	

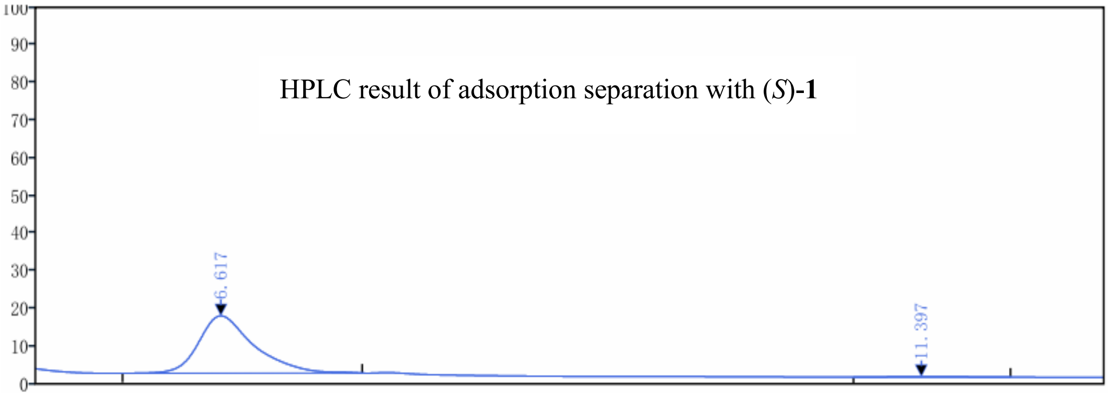

Serial Number	Type	Retention Time [min]	Peak Area	Area %
1	MM	6.617	530.41	99.07
2	MM	11.397	5.01	0.93
The Total			535.42	

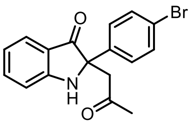

Chiral HPLC analysis: Daicel Chiralce OD; hexane/*i*-PrOH = 80/20, flow rate = 1 mL/min, 220 nm, t_R = 10.160 min, t_R = 15.977 min.

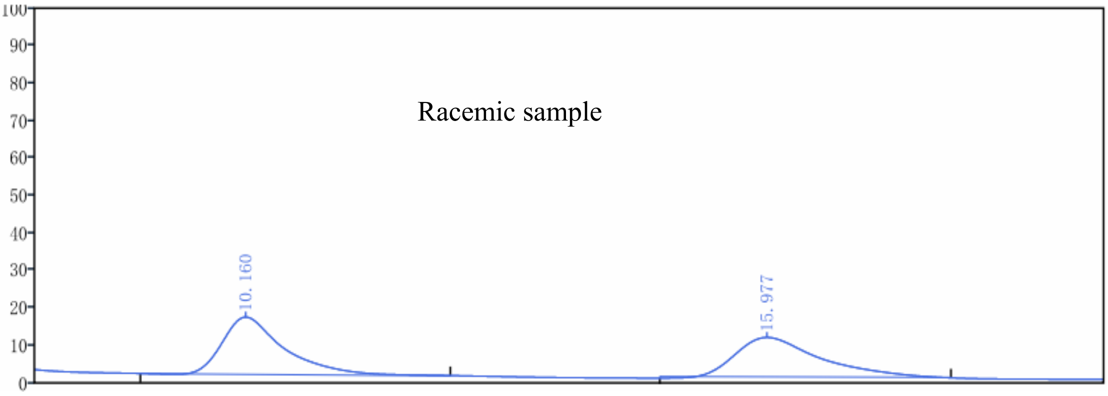

Serial Number	Type	Retention Time [min]	Peak Area	Area %
1	MM	10.160	1723.39	52.08
2	MM	15.977	1585.74	47.92
The Total			3309.13	

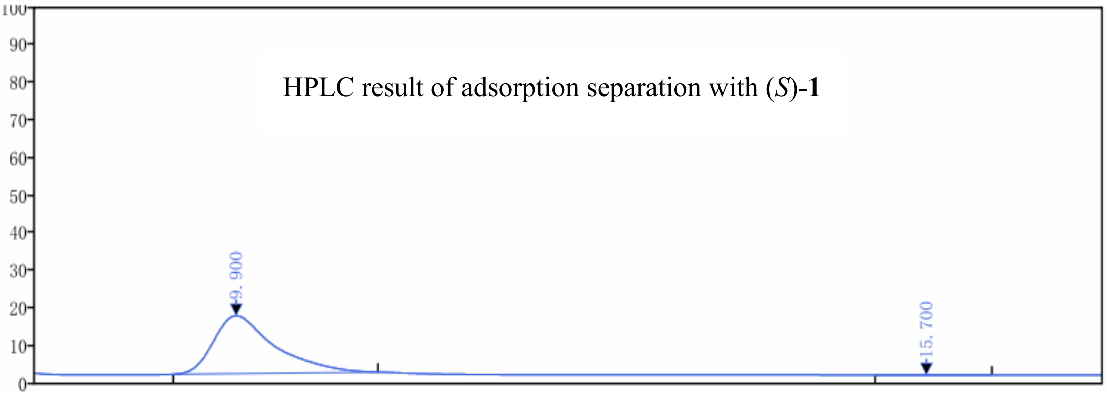

Serial Number	Type	Retention Time [min]	Peak Area	Area %
1	MM	9.900	790.07	99.99
2	MM	15.700	0.08	0.01
The Total			790.15	

21. Figure S18. HPLC spectra of *α*-phenylethylamine and limonene.

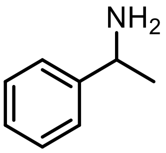

Chiral HPLC analysis: Daicel Chiralce OD-H: hexane/*i*-PrOH = 95:5; flow rate = 1.0 mL/min; 254 nm; t_R = 9.904 min, t_R = 13.259 min.

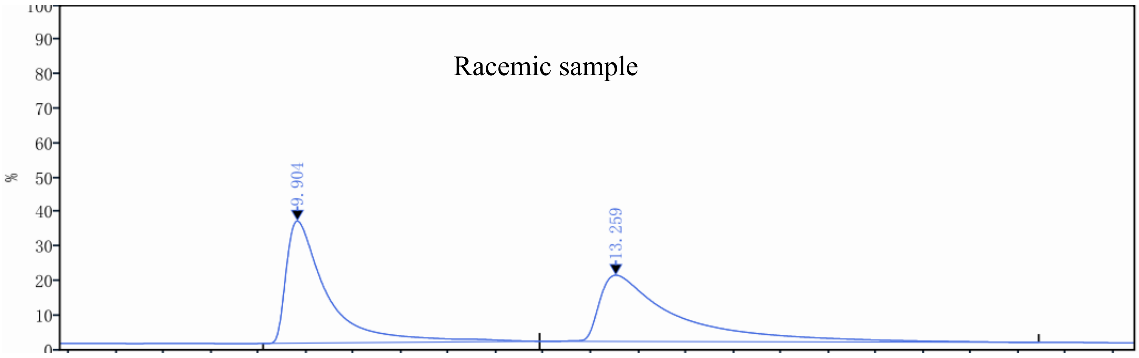

Serial Number	Type	Retention Time [min]	Peak Area	Area %
1	MM	9.904	4427.11	49.94
2	MM	13.259	4438.13	50.06
The Total			304.81	

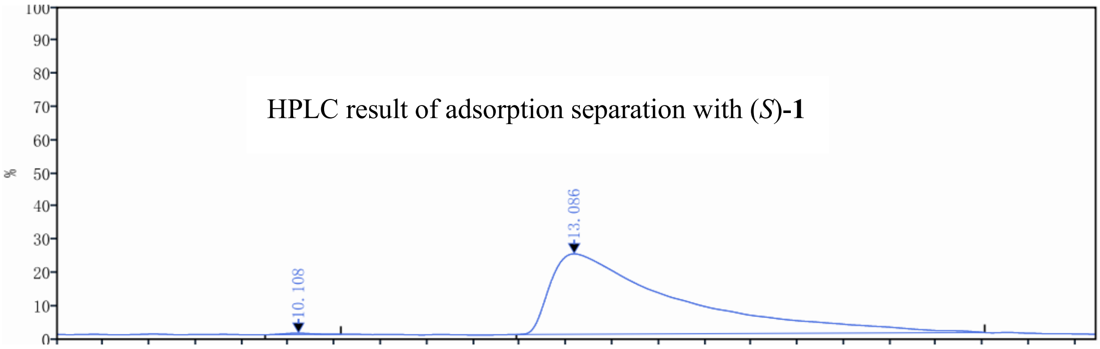

Serial Number	Type	Retention Time [min]	Peak Area	Area %
1	MM	10.108	4.84	0.38
2	MM	13.086	1265.32	99.62
The Total			1270.16	

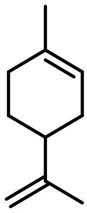 Agilent CP-Chirasil-DEX CB (0.25 mm × 25 m, df = 0.25 μm), Temperature : 85 ℃ continue 30 min, Split, FID:300 °C, t_R = 19.793 min, t_R = 20.440 min.

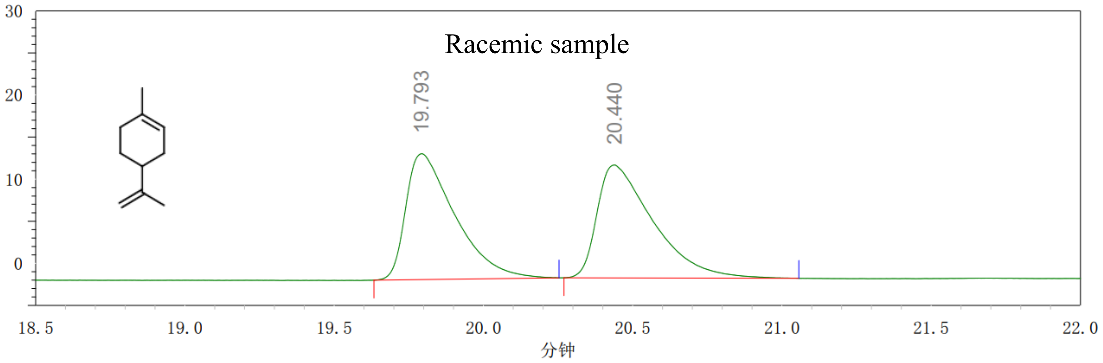

Serial Number	Retention Time [min]	Peak Area	Area %
1	19.793	1312826	49.53
2	20.440	1337944	50.47
The Total		2650770	

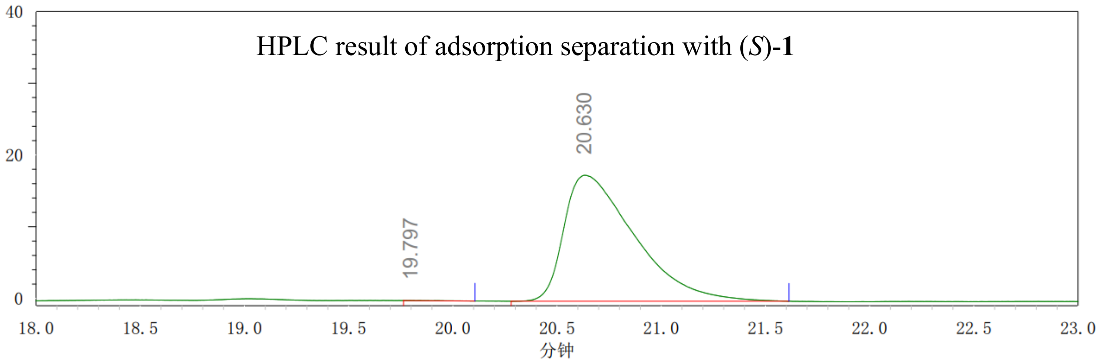

Serial Number	Retention Time [min]	Peak Area	Area %
1	19.797	1586	0.05
2	20.630	3177990	99.95
The Total		3179576	

22. Figure S19. HPLC spectra of chiral drugs (in Figure 4).

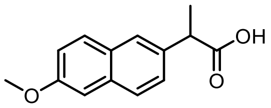

Chiral HPLC analysis: Daicel Chiralce AD-H: hexane/*i*-PrOH = 75/25; flow rate =0.5 mL/min; 272nm; t_R = 14.578 min, t_R = 16.319 min.

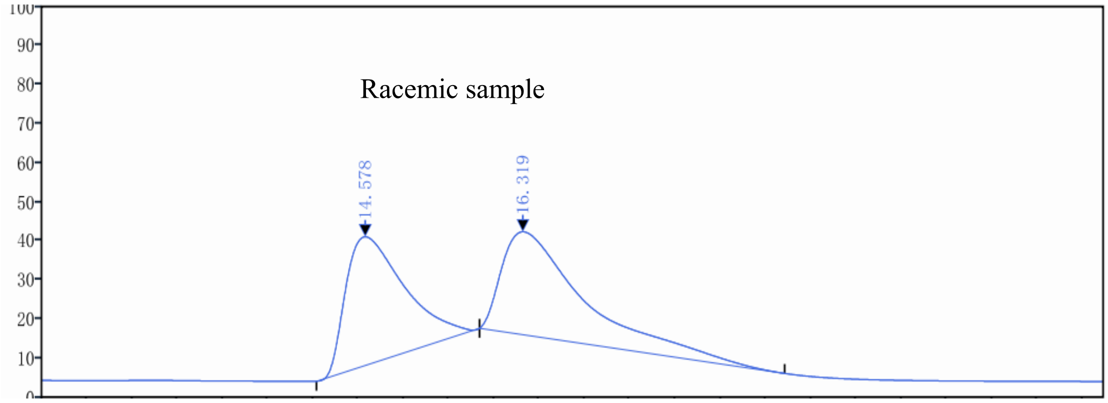

Serial Number	Type	Retention Time [min]	Peak Area	Area %
1	MM	14.578	9664.21	46.63
2	MM	16.319	11061.54	53.37
The Total			20725.75	

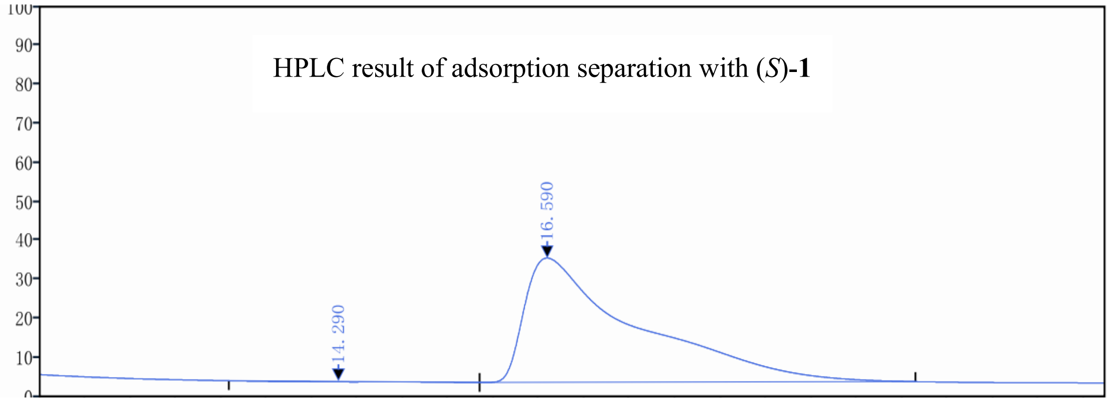

Serial Number	Type	Retention Time [min]	Peak Area	Area %
1	MM	14.290	15.82	0.26
2	MM	16.590	6121.45	99.74
The Total			6137.27	

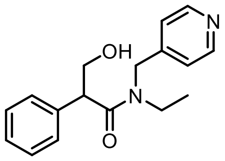

Chiral HPLC analysis: Daicel Chiralce IA: hexane/*i*-PrOH = 70/30; flow rate =0.6 mL/min; 254 nm; t_R = 12.233 min, t_R = 17.052 min.

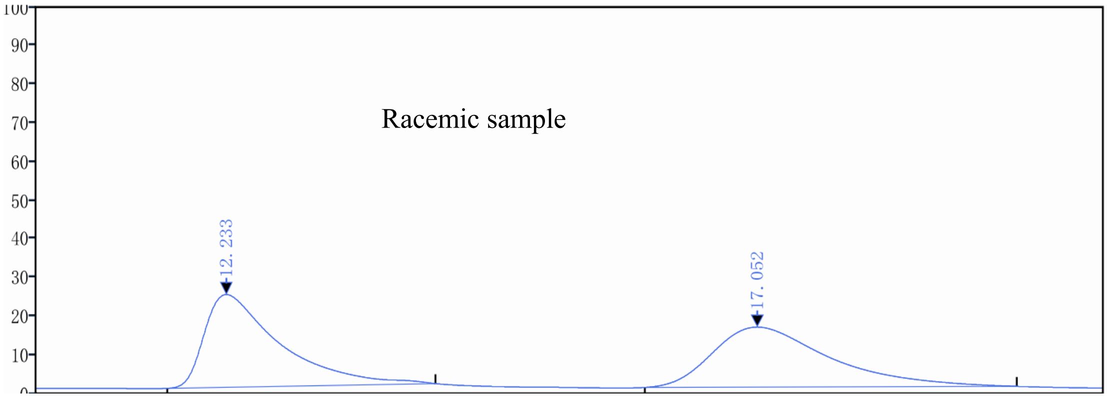

Serial Number	Type	Retention Time [min]	Peak Area	Area %
1	MM	12.233	910.42	49.83
2	MM	17.052	916.81	50.17
The Total			1827.23	

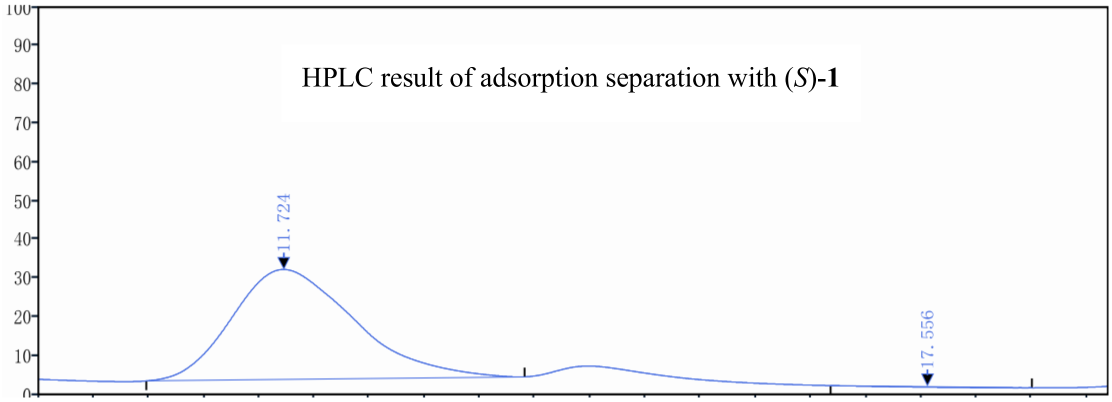

Serial Number	Type	Retention Time [min]	Peak Area	Area %
1	MM	11.724	681.85	99.57
2	MM	17.556	2.94	0.43
The Total			684.80	

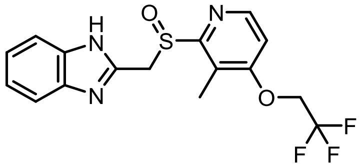

Chiral HPLC analysis: Daicel Chiralce AD-3: hexane/*i*-PrOH = 70/30; flow rate = 1.0 mL/min; 220 nm; t_R = 12.160 min, t_R = 13.014 min.

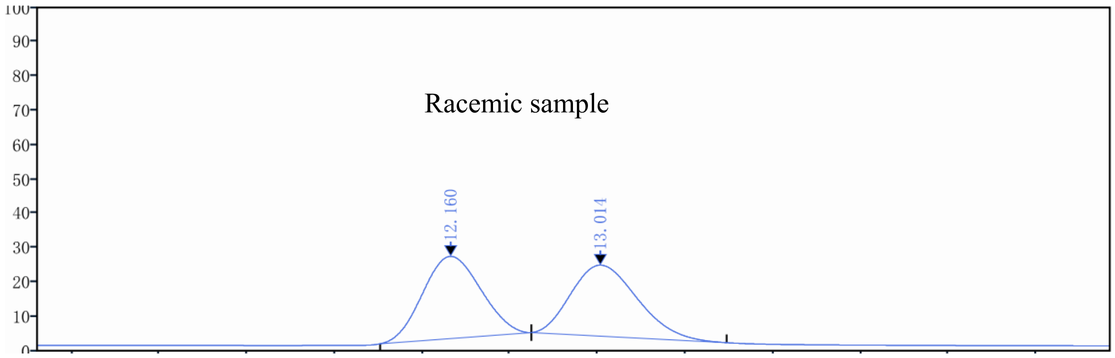

Serial Number	Type	Retention Time [min]	Peak Area	Area %
1	MM	12.160	3354.97	50.23
2	MM	13.014	3324.75	49.77
The Total			6679.72	

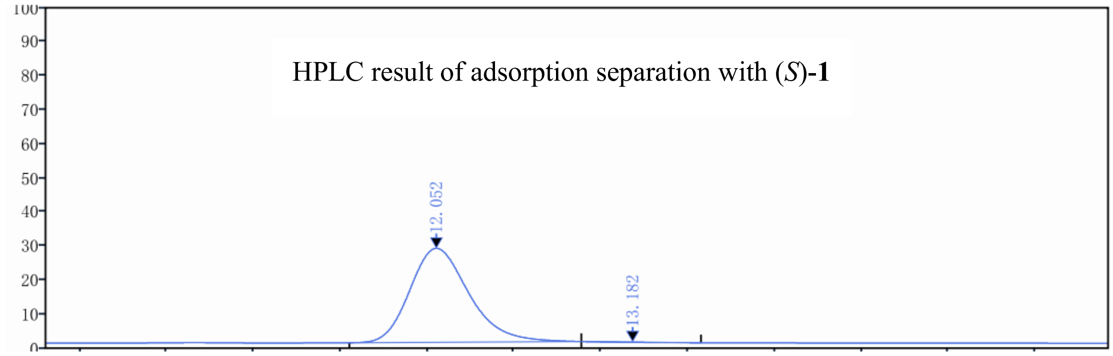

Serial Number	Type	Retention Time [min]	Peak Area	Area %
1	MM	12.052	4122.40	99.67
2	MM	13.182	13.60	0.33
The Total			4135.99	

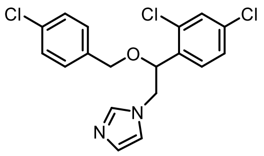

Chiral HPLC analysis: Daicel Chiralce OJ-H: hexane/*i*-PrOH = 76:24; flow rate = 0.6 mL/min; 254 nm; t_R = 25.631 min, t_R = 35.531 min.

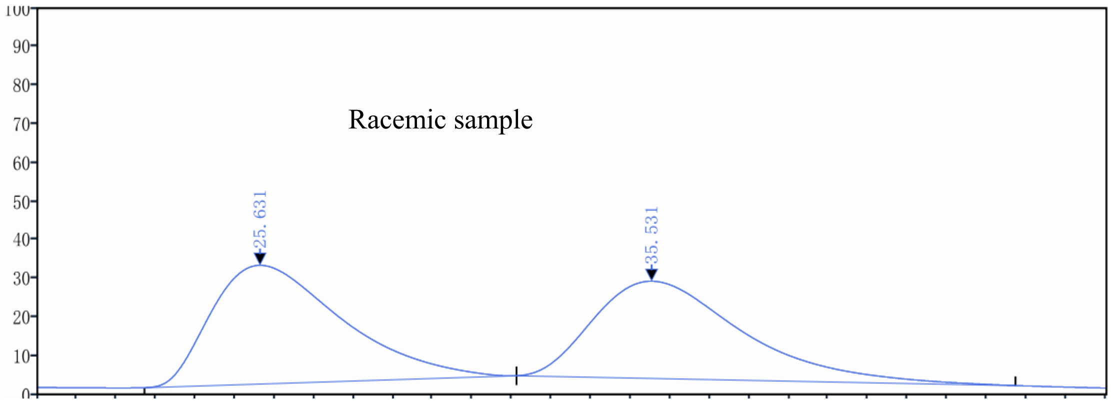

Serial Number	Type	Retention Time [min]	Peak Area	Area %
1	MM	25.631	12967.83	51.74
2	MM	35.531	12095.07	48.26
The Total			25062.91	

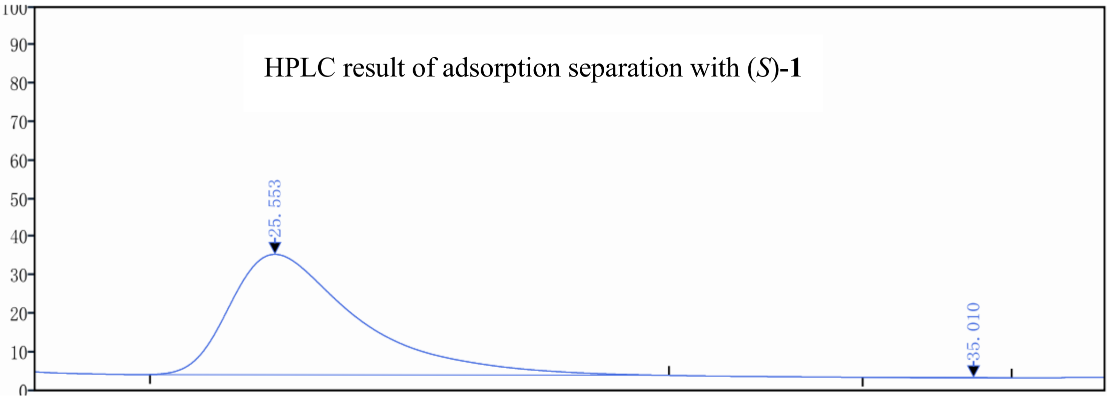

Serial Number	Type	Retention Time [min]	Peak Area	Area %
1	MM	25.553	1472.03	99.99
2	MM	35.010	0.22	0.01
The Total			1472.25	

21. References.

1. Dolomanov, O. V.; Bourhis, L. J.; Gildea, R. J.; Howard, J. A. K.; Puschmann, H., J. Appl. Crystallogr. 2009, 42 (2), 339-341.
2. Spek, A., Acta Crystallographica Section C 2015, 71 (1), 9-18.
3. Materials Studio 8.0. Dassault Systemes BIOVIA: San Diego, 2014.
